# THE CONCISE GUIDE TO PHARMACOLOGY 2017/18: Enzymes

**DOI:** 10.1111/bph.13877

**Published:** 2017-10-21

**Authors:** Stephen PH Alexander, Doriano Fabbro, Eamonn Kelly, Neil V Marrion, John A Peters, Elena Faccenda, Simon D Harding, Adam J Pawson, Joanna L Sharman, Christopher Southan, Jamie A Davies, CGTP Collaborators

**Affiliations:** ^1^ School of Life Sciences University of Nottingham Medical School Nottingham NG7 2UH UK; ^2^ PIQUR Therapeutics Basel 4057 Switzerland; ^3^ School of Physiology, Pharmacology and Neuroscience University of Bristol Bristol BS8 1TD UK; ^4^ Neuroscience Division, Medical Education Institute, Ninewells Hospital and Medical School University of Dundee Dundee DD1 9SY UK; ^5^ Centre for Integrative Physiology University of Edinburgh Edinburgh EH8 9XD UK

## Abstract

The Concise Guide to PHARMACOLOGY 2017/18 provides concise overviews of the key properties of nearly 1800 human drug targets with an emphasis on selective pharmacology (where available), plus links to an open access knowledgebase of drug targets and their ligands (www.guidetopharmacology.org), which provides more detailed views of target and ligand properties. Although the Concise Guide represents approximately 400 pages, the material presented is substantially reduced compared to information and links presented on the website. It provides a permanent, citable, point‐in‐time record that will survive database updates. The full contents of this section can be found at http://onlinelibrary.wiley.com/doi/10.1111/bph.13877/full. Enzymes are one of the eight major pharmacological targets into which the Guide is divided, with the others being: G protein‐coupled receptors, ligand‐gated ion channels, voltage‐gated ion channels, other ion channels, nuclear hormone receptors, catalytic receptors and transporters. These are presented with nomenclature guidance and summary information on the best available pharmacological tools, alongside key references and suggestions for further reading. The landscape format of the Concise Guide is designed to facilitate comparison of related targets from material contemporary to mid‐2017, and supersedes data presented in the 2015/16 and 2013/14 Concise Guides and previous Guides to Receptors and Channels. It is produced in close conjunction with the Nomenclature Committee of the Union of Basic and Clinical Pharmacology (NC‐IUPHAR), therefore, providing official IUPHAR classification and nomenclature for human drug targets, where appropriate.

## Conflict of interest

The authors state that there are no conflicts of interest to declare.

## Overview

Enzymes are protein catalysts facilitating the conversion of substrates into products. The Nomenclature Committee of the International Union of Biochemistry and Molecular Biology (NC‐IUBMB) classifies enzymes into families, using a four number code, on the basis of the reactions they catalyse. There are six main families:

EC 1.‐.‐.‐ Oxidoreductases;

EC 2.‐.‐.‐ Transferases;

EC 3.‐.‐.‐ Hydrolases;

EC 4.‐.‐.‐ Lyases;

EC 5.‐.‐.‐ Isomerases;

EC 6.‐.‐.‐ Ligases.

Although there are many more enzymes than receptors in biology, and many drugs that target prokaryotic enzymes are effective medicines, overall the number of enzyme drug targets is relatively small [392, 430], which is not to say that they are of modest importance.

The majority of drugs which act on enzymes act as inhibitors; one exception is metformin, which appears to stimulate activity of AMP‐activated protein kinase, albeit through an imprecisely‐defined mechanism. Kinetic assays allow discrimination of competitive, non‐competitive, and un‐competitive inhibitors. The majority of inhibitors are competitive (acting at the enzyme's ligand recognition site), non‐competitive (acting at a distinct site; potentially interfering with co‐factor or co‐enzyme binding) or of mixed type. One rare example of an uncompetitive inhibitor is lithium ions, which are effective inhibitors at inositol monophosphatase only in the presence of high substrate concentrations. Some inhibitors are irreversible, including a group known as suicide substrates, which bind to the ligand recognition site and then couple covalently to the enzyme. It is beyond the scope of the Guide to give mechanistic information about the inhibitors described, although generally this information is available from the indicated literature.

Many enzymes require additional entities for functional activity. Some of these are used in the catalytic steps, while others promote a particular conformational change. Co‐factors are tightly bound to the enzyme and include metal ions and heme groups. Co‐enzymes are typically small molecules which accept or donate functional groups to assist in the enzymatic reaction. Examples include ATP, NAD, NADP and S‐adenosylmethionine, as well as a number of vitamins, such as riboflavin (vitamin B1) and thiamine (vitamin B2). Where co‐factors/co‐enzymes have been identified, the Guide indicates their involvement.

## Family structure

S275 Kinases (EC 2.7.x.x)

– AGC: Containing PKA, PKG, PKC families

– DMPK family

– GEK subfamily

– Other DMPK family kinases

S276 Rho kinase

– G protein‐coupled receptor kinases (GRKs)

– Beta‐adrenergic receptor kinases (βARKs)

– Opsin/rhodopsin kinases

– GRK4 subfamily

– MAST family

– NDR family

– PDK1 family

– Protein kinase A

– Akt (Protein kinase B)

S276 Protein kinase C (PKC)

S277 Alpha subfamily

S277 Delta subfamily

S277 Eta subfamily

– Iota subfamily

– Protein kinase G (PKG)

– Protein kinase N (PKN) family

– RSK family

– MSK subfamily

– p70 subfamily

– RSK subfamily

– RSKR subfamily

– RSKL family

– SGK family

– YANK family

– Atypical

– ABC1 family

– ABC1‐A subfamily

– ABC1‐B subfamily

– Alpha kinase family

– ChaK subfamily

– eEF2K subfamily

– Other alpha kinase family kinases

– BCR family

– Bromodomain kinase (BRDK) family

– G11 family

– Phosphatidyl inositol 3' kinase‐related kinases (PIKK) family

– ATR subfamily

S278 FRAP subfamily

– SMG1 subfamily

– TRRAP subfamily

– Other PIKK family kinases

– RIO family

– RIO1 subfamily

– RIO2 subfamily

– RIO3 subfamily

– PDHK family

– Pyruvate dehydrogenase kinase (PDHK) family

– TAF1 family

– TIF1 family

– CAMK: Calcium/calmodulin‐dependent protein kinases

– CAMK1 family

– CAMK2 family

– CAMK‐like (CAMKL) family

– AMPK subfamily

– BRSK subfamily

– CHK1 subfamily

– HUNK subfamily

– LKB subfamily

– MARK subfamily

– MELK subfamily

– NIM1 subfamily

– NuaK subfamily

– PASK subfamily

– QIK subfamily

– SNRK subfamily

– CAMK‐unique family

– CASK family

– DCAMKL family

– Death‐associated kinase (DAPK) family

– MAPK‐Activated Protein Kinase (MAPKAPK) family

– MAPKAPK subfamily

– MKN subfamily

– Myosin Light Chain Kinase (MLCK) family

– Phosphorylase kinase (PHK) family

– PIM family

– Protein kinase D (PKD) family

– PSK family

– RAD53 family

– Testis specific kinase (TSSK) family

– Trbl family

– Trio family

– CK1: Casein kinase 1

– Casein kinase 1 (CK1) family

– Tau tubulin kinase (TTBK) family

– Vaccina related kinase (VRK) family

– CMGC: Containing CDK, MAPK, GSK3, CLK families

– CLK family

S279 Cyclin‐dependent kinase (CDK) family

– CCRK subfamily

– CDK1 subfamily

S279 CDK4 subfamily

– CDK5 subfamily

– CDK7 subfamily

– CDK8 subfamily

– CDK9 subfamily

– CDK10 subfamily

– CRK7 subfamily

– PITSLRE subfamily

– TAIRE subfamily

– Cyclin‐dependent kinase‐like (CDKL) family

– Dual‐specificity tyrosine‐(Y)‐phosphorylation regulated kinase (DYRK) family

– Dyrk1 subfamily

– Dyrk2 subfamily

– HIPK subfamily

– PRP4 subfamily

– Glycogen synthase kinase (GSK) family

S279 GSK subfamily

– Mitogen‐activated protein kinases (MAP kinases)

– ERK subfamily

– Erk7 subfamily

– JNK subfamily

– p38 subfamily

– nmo subfamily

– RCK family

– SRPK family

– Other protein kinases

– CAMKK family

– Meta subfamily

– Aurora kinase (Aur) family

– Bub family

– Bud32 family

– Casein kinase 2 (CK2) family

– CDC7 family

– Haspin family

– IKK family

– IRE family

– MOS family

– NAK family

– NIMA (never in mitosis gene a)

‐ related kinase (NEK) family

– NKF1 family

– NKF2 family

– NKF4 family

– NKF5 family

– NRBP family

– Numb‐associated kinase (NAK) family

– Other‐unique family

S280 Polo‐like kinase (PLK) family

– PEK family

– GCN2 subfamily

– PEK subfamily

– Other PEK family kinases

– SgK493 family

– Slob family

– TBCK family

– TOPK family

– Tousled‐like kinase (TLK) family

– TTK family

– Unc‐51‐like kinase (ULK) family

– VPS15 family

– WEE family

– Wnk family

– Miscellaneous protein kinases

– actin‐binding proteins ADF family

– Twinfilin subfamily

– SCY1 family

– Hexokinases

– STE: Homologs of yeast Sterile 7, Sterile 11, Sterile 20 kinases

S280 STE7 family

– STE11 family

– STE20 family

– FRAY subfamily

– KHS subfamily

– MSN subfamily

– MST subfamily

– NinaC subfamily

– PAKA subfamily

– PAKB subfamily

– SLK subfamily

– STLK subfamily

– TAO subfamily

– YSK subfamily

– STE20 family

– STE‐unique family

– TK: Tyrosine kinase

– Non‐receptor tyrosine kinases (nRTKs)

S281 Abl family

S281 Ack family

– Csk family

– Fak family

– Fer family

S281 Janus kinase (JakA) family

S282 Src family

– Syk family

S282 Tec family

– TKL: Tyrosine kinase‐like

– Interleukin‐1 receptor‐associated kinase (IRAK) family

– Leucine‐rich repeat kinase (LRRK) family

– LIM domain kinase (LISK) family

– LIMK subfamily

– TESK subfamily

– Mixed Lineage Kinase (MLK) family

– HH498 subfamily

– ILK subfamily

– LZK subfamily

– MLK subfamily

– TAK1 subfamily

S283 RAF family

– Receptor interacting protein kinase (RIPK) family

– TKL‐unique family

S284 Peptidases and proteinases

– AA: Aspartic (A) Peptidases

S284 A1: Pepsin

– AD: Aspartic (A) Peptidases

S284 A22: Presenilin

– CA: Cysteine (C) Peptidases

– C1: Papain

– C2: Calpain

– C12: Ubiquitin C‐terminal hydrolase

– C19: Ubiquitin‐specific protease

– C54: Aut2 peptidase

– C101: OTULIN peptidase

– CD: Cysteine (C) Peptidases

– C13: Legumain

S284 C14: Caspase

– CE: Cysteine (C) Peptidases

– C48: Ulp1 endopeptidase

– M‐: Metallo (M) Peptidases

– M79: Prenyl protease 2

– MA: Metallo (M) Peptidases

S285 M1: Aminopeptidase N

S285 M2: Angiotensin‐converting (ACE and ACE2)

S286 M10: Matrix metallopeptidase

S286 M12: Astacin/Adamalysin

– M13: Neprilysin

– M49: Dipeptidyl‐peptidase III

– MC: Metallo (M) Peptidases

– M14: Carboxypeptidase A

– ME: Metallo (M) Peptidases

– M16: Pitrilysin

– MF: Metallo (M) Peptidases

– M17: Leucyl aminopeptidase

– MG: Metallo (M) Peptidases

– M24: Methionyl aminopeptidase

– MH: Metallo (M) Peptidases

– M18: Aminopeptidase I

– M20: Carnosine dipeptidase

S286 M28: Aminopeptidase Y

– MJ: Metallo (M) Peptidases

S287 M19: Membrane dipeptidase

– MP: Metallo (M) Peptidases

– M67: PSMD14 peptidase

– PA: Serine (S) Peptidases

S287 S1: Chymotrypsin

– PB: Threonine (T) Peptidases

– C44: Phosphoribosyl pyrophosphate amidotransferase

S288 T1: Proteasome

– T2: Glycosylasparaginase precursor

– PC: Cysteine (C) Peptidases

– C26: Gamma‐glutamyl hydrolase

– SB: Serine (S) Peptidases

S289 S8: Subtilisin

– SC: Serine (S) Peptidases

S289 S9: Prolyl oligopeptidase

– S10: Carboxypeptidase Y

– S28: Lysosomal Pro‐Xaa carboxypeptidase

– S33: Prolyl aminopeptidase

– AAA ATPases

S290 Acetylcholine turnover

S291 Adenosine turnover

S292 Amino acid hydroxylases

S293 L‐Arginine turnover

S294 2.1.1.‐ Protein arginine N‐methyltransferases

S294 Arginase

S294 Arginine:glycine amidinotransferase

S294 Dimethylarginine dimethylaminohydrolases

S295 Nitric oxide synthases

S296 Carboxylases and decarboxylases

S296 Carboxylases

S298 Decarboxylases

S299 Catecholamine turnover

S302 Ceramide turnover

S302 Serine palmitoyltransferase

– 3‐ketodihydrosphingosine reductase

S303 Ceramide synthase

S304 Sphingolipid Δ^4^‐desaturase

S304 Sphingomyelin synthase

S305 Sphingomyelin phosphodiesterase

S305 Neutral sphingomyelinase coupling factors

S305 Ceramide glucosyltransferase

S306 Acid ceramidase

S306 Neutral ceramidases

S307 Alkaline ceramidases

S307 Ceramide kinase

S308 Chromatin modifying enzymes

– Enzymatic bromodomain‐containing proteins

– Bromodomain kinase (BRDK) family

– TAF1 family

– TIF1 family

– 1.14.11.‐ Histone demethylases

S309 2.1.1.‐ Protein arginine N‐methyltransferases

– 2.1.1.43 Histone methyltransferases (HMTs)

– 2.3.1.48 Histone acetyltransferases (HATs)

S309 3.5.1.‐ Histone deacetylases (HDACs)

– 3.6.1.3 ATPases

S310 Cyclic nucleotide turnover/signalling

S310 Adenylyl cyclases (ACs)

S311 Exchange protein activated by cyclic AMP (EPACs)

S312 Nitric oxide (NO)‐sensitive (soluble) guanylyl cyclase

S313 Phosphodiesterases, 3Š,5Š‐cyclic nucleotide (PDEs)

S317 Cytochrome P450

S317 CYP1 family

S317 CYP2 family

S318 CYP3 family

S319 CYP4 family

S320 CYP5, CYP7 and CYP8 families

S320 CYP11, CYP17, CYP19, CYP20 and CYP21 families

S321 CYP24, CYP26 and CYP27 families

S321 CYP39, CYP46 and CYP51 families

S323 Endocannabinoid turnover

S323 N‐Acylethanolamine turnover

S324 2‐Acylglycerol ester turnover

S325 Eicosanoid turnover

S325 Cyclooxygenase

S326 Prostaglandin synthases

S327 Lipoxygenases

S328 Leukotriene and lipoxin metabolism

S329 GABA turnover

S330 Glycerophospholipid turnover

S331 Phosphoinositide‐specific phospholipase C

S332 Phospholipase A2

S333 Phosphatidylcholine‐specific phospholipase D

S334 Lipid phosphate phosphatases

S335 Phosphatidylinositol kinases

S336 Phosphatidylinositol phosphate kinases

S339 Haem oxygenase

S340 Hydrogen sulphide synthesis

S341 Hydrolases

S342 Inositol phosphate turnover

S342 Inositol 1,4,5‐trisphosphate 3‐kinases

S343 Inositol polyphosphate phosphatases

S343 Inositol monophosphatase

S344 Lanosterol biosynthesis pathway

– LPA synthesis

S346 Nucleoside synthesis and metabolism

S347 Sphingosine 1‐phosphate turnover

S348 Sphingosine kinase

S348 Sphingosine 1‐phosphate phosphatase

S349 Sphingosine 1‐phosphate lyase

S349 Thyroid hormone turnover

– 1.‐.‐.‐ Oxidoreductases

– 1.1.1.42 Isocitrate dehydrogenases

– 1.4.3.13 Lysyl oxidases

– 1.13.11.‐ Dioxygenases

S350 1.14.11.29 2‐oxoglutarate oxygenases

S351 1.14.13.9 kynurenine 3‐monooxygenase

– 1.17.4.1 Ribonucleoside‐diphosphate reductases

– 2.1.1.‐ Methyltransferases

– 2.1.2.‐ Hydroxymethyl‐, formyl‐ and related transferases

– 2.3.‐.‐ Acyltransferases

– 2.4.2.1 Purine‐nucleoside phosphorylase

S351 2.4.2.30 poly(ADP‐ribose)polymerases

S352 2.5.1.58 Protein farnesyltransferase

– 2.6.1.42 Branched‐chain‐amino‐acid transaminase

– 3.1.‐.‐ Ester bond enzymes

– 3.1.1.‐ Carboxylic Ester Hydrolases

– 3.2.1.‐ Glycosidases

– 3.4.21.46 Complement factor D

S353 3.5.1.‐ Histone deacetylases (HDACs)

– 3.5.1.2 Glutaminases

S353 3.5.3.15 Peptidyl arginine deiminases (PADI)

– 3.6.5.2 Small monomeric GTPases

S354 RAS subfamily

– RAB subfamily

S355 4.2.1.1 Carbonate dehydratases

– 5.‐.‐.‐ Isomerases

– 5.2.‐.‐ Cis‐trans‐isomerases

S355 5.99.1.2 DNA Topoisomerases

– 6.3.3.‐ Cyclo‐ligases

## Kinases (EC 2.7.x.x)

### Overview

Protein kinases (E.C. 2.7.11.‐) use the co‐substrate ATP to phosphorylate serine and/or threonine residues on target proteins. Analysis of the human genome suggests the presence of 518 protein kinases in man (divided into 15 subfamilies), with over 100 protein kinase‐like pseudogenes [335]. It is beyond the scope of the Concise Guide to list all these protein kinase activities, but full listings are available on the 'Detailed page' provided for each enzyme.

Most inhibitors of these enzymes have been assessed in cell‐free investigations and so may appear to 'lose' potency and selectivity in intact cell assays. In particular, ambient ATP concentrations may be influential in responses to inhibitors, since the majority are directed at the ATP binding site [110] .

## Rho kinase

### Overview

Rho kinase (also known as P160ROCK, Rho‐activated kinase) is activated by members of the Rho small G protein family, which are activated by GTP exchange factors, such as ARHGEF1(Q92888, p115‐RhoGEF), which in turn may be activated by G*α*_12/13_ subunits [282].

**Table:** 

Nomenclature	Rho associated coiled‐coil containing protein kinase 1	Rho associated coiled‐coil containing protein kinase 2
Systematic nomenclature	ROCK1	ROCK2
HGNC, UniProt	ROCK1, Q13464	ROCK2, O75116
EC number	2.7.11.1	2.7.11.1
Common abreviation	Rho kinase 1	Rho kinase 2
Inhibitors	RKI‐1447 (pIC_50_>9) [414], Y27632 (pIC_50_ 5.9–7.3) [328, 575], fasudil (p*K*_i_ 7) [434], Y27632 (p*K*_i_ 6.8) [540], fasudil (pIC_50_ 5.5–5.6) [328, 434]	RKI‐1447 (pIC_50_>9) [414], compound 11d [DOI: 10.1039/c0md00194e] (pIC_50_>9) [90], GSK269962A (pIC_50_ 8.4) [126], compound 32 (pIC_50_ 8.4) [49], compound 22 (pIC_50_ 7.7) [575], Y27632 (pIC_50_ 6.3–7.2) [328, 575], Y27632 (p*K*_i_ 6.8–6.9) [328, 540], fasudil (pIC_50_ 5.9–5.9) [328, 434]
Selective inhibitors	GSK269962A (pIC_50_ 8.8) [126]	–

### Further reading on Rho kinases

Feng, Y, PV LoGrasso, O Defert and R Li 2016 Rho Kinase (ROCK) Inhibitors and Their Therapeutic Potential J Med Chem 59: 2269‐300 [PMID:26486225]

Nishioka, T, MH Shohag, M Amano and K Kaibuchi 2015 Developing novel methods to search for substrates of protein kinases such as Rho‐kinase Biochim Biophys Acta 1854: 1663‐6 [PMID:25770685]

Shimokawa, H, S Sunamura and K Satoh 2016 RhoA/Rho‐Kinase in the Cardiovascular System Circ Res 118: 352‐66 [PMID:26838319]

## Protein kinase C (PKC)

### Overview

Protein kinase C is the target for the tumour‐promoting phorbol esters, such as tetradecanoyl‐*β*‐phorbol acetate (TPA, also known as phorbol 12‐myristate 13‐acetate).

Classical protein kinase C isoforms: PKC*α*, PKC*β*, and PKC*γ* are activated by Ca^2+^ and diacylglycerol, and may be inhibited by GF109203X, calphostin C, Gö 6983, chelerythrine and Ro31‐8220.

Novel protein kinase C isoforms: PKC*δ*, PKC*ϵ*, PKC*η*, PKC*θ* and PKC*μ* are activated by diacylglycerol and may be inhibited by calphostin C, Gö 6983 and chelerythrine.

Atypical protein kinase C isoforms:PKC*ι*, PKC*ζ*.

## Alpha subfamily

**Table:** 

Nomenclature	protein kinase C beta	protein kinase C gamma
HGNC, UniProt	PRKCB, P05771	PRKCG, P05129
EC number	2.7.11.13	2.7.11.13
Common abreviation	PKC*β*	PKC*γ*
Inhibitors	sotrastaurin (pIC_50_ 8.7) [548], Gö 6983 (pIC_50_ 8.1) [195], GF109203X (pIC_50_ 7.8) [533] – Bovine, 7‐hydroxystaurosporine (pIC_50_ 7.5) [468]	Gö 6983 (pIC_50_ 8.2) [195],7‐hydroxystaurosporine (pIC_50_ 7.5) [469]
Selective inhibitors	ruboxistaurin (pIC_50_ 8.2) [250], enzastaurin (pIC_50_ 7.5) [140], CGP53353 (pIC_50_ 6.4) [75]	–

## Delta subfamily

**Table:** 

Nomenclature	protein kinase C alpha	protein kinase C delta
HGNC, UniProt	PRKCA, P17252	PRKCD, Q05655
EC number	2.7.11.13	2.7.11.13
Common abreviation	PKC*α*	PKC*δ*
Activators	–	ingenol mebutate (p*K*_i_ 9.4) [263]
Inhibitors	sotrastaurin (pIC_50_ 8.7) [548], Gö 6983 (pIC_50_ 8.1) [195], 7‐hydroxystaurosporine (pIC_50_ 7.5) [468]	sotrastaurin (pIC_50_ 8.9) [548], Gö 6983 (pIC_50_ 8) [195]

## Eta subfamily

**Table:** 

Nomenclature	protein kinase C epsilon
HGNC, UniProt	PRKCE, Q02156
EC number	2.7.11.13
Common abreviation	PKC*ϵ*
Inhibitors	sotrastaurin (pIC_50_ 8.2) [548]

### Further reading on Protein kinase C

Igumenova TI. (2015) Dynamics and Membrane Interactions of Protein Kinase C. Biochemistry 54: 4953‐68 [PMID:26214365]

Newton AC et al. (2017) Reversing the Paradigm: Protein Kinase C as a Tumor Suppressor. Trends Pharmacol Sci 38: 438‐447 [PMID:28283201]

Salzer E et al. (2016) Protein Kinase C delta: a Gatekeeper of Immune Homeostasis. J Clin Immunol 36: 631‐40 [PMID:27541826]

## FRAP subfamily

**Table:** 

Nomenclature	mechanistic target of rapamycin
HGNC, UniProt	MTOR, P42345
EC number	2.7.11.1
Common abreviation	mTOR
Inhibitors	ridaforolimus (pIC_50_ 9.7) [441], torin 1 (pIC_50_ 9.5) [310], INK‐128 (pIC_50_ 9) [231], INK‐128 (p*K*_i_ 8.9) [231], gedatolisib (pIC_50_ 8.8) [544], dactolisib (pIC_50_ 8.2) [332], PP‐242 (pIC_50_ 8.1) [15], PP121 (pIC_50_ 8) [15], XL388 (pIC_50_ 8) [511], PF‐04691502 (p*K*_i_ 7.8) [309], apitolisib (p*K*_i_ 7.8) [506]
Selective inhibitors	everolimus (pIC_50_ 8.7) [464], temsirolimus (pIC_50_ 5.8) [278]

### Further reading on FRAP subfamily

Hukelmann JL et al. (2016) The cytotoxic T cell proteome and its shaping by the kinase mTOR. Nat. Immunol. 17: 104‐12 [PMID:26551880]

Saxton RA et al. (2017) mTOR Signaling in Growth, Metabolism, and Disease. Cell 169: 361‐371 [PMID:28388417]

## Cyclin‐dependent kinase (CDK) family

### Overview

The development of CDK inhibitors as anticancer drugs is reviewed in [508], with detailed content covering CDK4 and CDK6 inhibitors under clinical evaluation.

## CDK4 subfamily

**Table:** 

Nomenclature	cyclin dependent kinase 4	cyclin dependent kinase 6
HGNC, UniProt	CDK4, P11802	CDK6, Q00534
EC number	2.7.11.22	2.7.11.22
Common abreviation	CDK4	CDK6
Inhibitors	R547 (p*K*_i_ 9) [117], palbociclib (pIC_50_ 8) [160], Ro‐0505124 (pIC_50_ 7.7) [129], riviciclib (pIC_50_ 7.2) [258], alvocidib (p*K*_i_ 7.2) [70]	palbociclib (pIC_50_ 7.8) [160]

## GSK subfamily

**Table:** 

Nomenclature	glycogen synthase kinase 3 beta
HGNC, UniProt	GSK3B, P49841
EC number	2.7.11.26
Common abreviation	GSK3B
Inhibitors	CHIR‐98014 (pIC_50_ 9.2) [440], LY2090314 (pIC_50_ 9) [133], CHIR‐99021 (pIC_50_ 8.2) [440], SB 216763 (pIC_50_∼8.1) [95], 1‐azakenpaullone (pIC_50_ 7.7) [285], SB‐415286 (pIC_50_∼7.4) [95], IM‐12 (pIC_50_ 7.3) [460]
Selective inhibitors	AZD2858 (p*K*_i_ 8.3) [31]
Comments	Due to its Tau phosphorylating activity, small molecule inhibitors of GSK‐3*β* are being investigated as potential treatments for Alzheimer's disease (AD) [31]. GSK‐3*β* also plays a role in canonical Wnt pathway signalling, the normal activity of which is crucial for the maintenance of normal bone mass. It is hypothesised that small molecule inhibitors of GSK‐3*β* may provide effective therapeutics for the treatment of diseases characterised by low bone mass [320].

### Further reading on GSK subfamily

Beurel E et al. (2015) Glycogen synthase kinase‐3 (GSK3): regulation, actions, and diseases. Pharmacol Ther 148: 114‐31 [PMID:25435019]

Domoto T et al. (2016) Glycogen synthase kinase‐3beta is a pivotal mediator of cancer invasion and resistance to therapy. Cancer Sci 107: 1363‐1372 [PMID:27486911]

Khan I et al. (2017) Natural and synthetic bioactive inhibitors of glycogen synthase kinase. Eur J Med Chem 125: 464‐477 [PMID:27689729]

Maqbool M et al. (2016) Pivotal role of glycogen synthase kinase‐3: A therapeutic target for Alzheimer's disease. Eur J Med Chem 107: 63‐81 [PMID:26562543]

## Polo‐like kinase (PLK) family

**Table:** 

Nomenclature	polo like kinase 4
HGNC, UniProt	PLK4, O00444
EC number	2.7.11.21
Common abreviation	PLK4
Inhibitors	CFI‐400945 (pIC_50_ 8.6) [343]

## STE7 family

**Table:** 

Nomenclature	mitogen‐activated protein kinase kinase 1	mitogen‐activated protein kinase kinase 2
HGNC, UniProt	MAP2K1, Q02750	MAP2K2, P36507
EC number	2.7.12.2	2.7.12.2
Common abreviation	MEK1	MEK2
Inhibitors	trametinib (pIC_50_ 9–9.1) [183, 589], PD 0325901 (pIC_50_ 8.1) [208]	trametinib (pIC_50_ 8.7) [589]
Allosteric modulators	binimetinib (Negative) (pIC_50_ 7.9) [428], refametinib (Negative) (pIC_50_ 7.7) [242], CI‐1040 (Negative) (p*K*_d_ 6.9) [112]	binimetinib (Negative) (pIC_50_ 7.9) [428], refametinib (Negative) (pIC_50_ 7.3) [242]
Selective allosteric modulators	cobimetinib (Negative) (pIC_50_ 9.1) [457]	–

## Abl family

**Table:** 

Nomenclature	ABL proto‐oncogene 1, non‐receptor tyrosine kinase
HGNC, UniProt	ABL1, P00519
EC number	2.7.10.2
Common abreviation	Abl
Inhibitors	compound 8h (pIC_50_ 9.7) [529], dasatinib (pIC_50_ 9.6) [270], compound 24 (pIC_50_ 9.3) [118], PD‐173955 (p*K*_d_ 9.2) [112], bosutinib (pIC_50_ 9) [186], PD‐173955 (pIC_50_∼8.3) [362], bafetinib (pIC_50_ 7.6–8.2) [228, 269], ponatinib (pIC_50_ 8.1) [232], nilotinib (pIC_50_ 7.8) [372], PP121 (pIC_50_ 7.7) [15], imatinib (pIC_50_ 6.7) [228], GNF‐5 (pIC_50_ 6.7) [597]

## Ack family

**Table:** 

Nomenclature	tyrosine kinase non receptor 2
HGNC, UniProt	TNK2, Q07912
EC number	2.7.10.2
Common abreviation	Ack
Inhibitors	compound 30 (pIC_50_ 9) [122]

## Janus kinase (JakA) family

**Table:** 

Nomenclature	Janus kinase 1	Janus kinase 2	Janus kinase 3	tyrosine kinase 2
HGNC, UniProt	JAK1, P23458	JAK2, O60674	JAK3, P52333	TYK2, P29597
EC number	2.7.10.2	2.7.10.2	2.7.10.2	2.7.10.2
Common abreviation	JAK1	JAK2	JAK3	Tyk2
Inhibitors	ruxolitinib (pIC_50_ 8.5–10.1) [203, 423], filgotinib (pIC_50_ 8) [541]	NS‐018 (pIC_50_ 9.1) [374], BMS‐911543 (pIC_50_ 9) [420], AT‐9283 (pIC_50_ 8.9) [230], XL019 (pIC_50_ 8.7) [152], fedratinib (pIC_50_ 8.5) [333, 566], gandotinib (pIC_50_ 8.4) [330]	AT‐9283 (pIC_50_ 9) [230]	–
Selective inhibitors	–	compound 1d (pIC_50_>9) [554]	–	–
Comments	–	The JAK2^V617F^ mutation, which causes constitutive activation, plays an oncogenic role in the pathogenesis of the myeloproliferative disorders, polycythemia vera, essential thrombocythemia, and idiopathic myelofibrosis [64, 115]. Small molecule compounds which inhibit aberrant JAK2 activity are being developed as novel anti‐cancer pharmaceuticals.	–	–

## Src family

**Table:** 

Nomenclature	BLK proto‐oncogene, Src family tyrosine kinase	fyn related Src family tyrosine kinase	FYN proto‐oncogene, Src family tyrosine kinase	LYN proto‐oncogene, Src family tyrosine kinase	SRC proto‐oncogene, non‐receptor tyrosine kinase
HGNC, UniProt	BLK, P51451	FRK, P42685	FYN, P06241	LYN, P07948	SRC, P12931
EC number	2.7.10.2	2.7.10.2	2.7.10.2	2.7.10.2	2.7.10.2
Common abreviation	Blk	FRK	Fyn	Lyn	Src
Inhibitors	–	–	PP1 (pIC_50_ 8.2) [205]	bafetinib (pIC_50_ 8) [228]	WH‐4‐023 (pIC_50_ 8.2) [340], PD166285 (p*K*_i_ 8.1) [396], PP121 (pIC_50_ 7.8) [15], ENMD‐2076 (pIC_50_ 7.7) [416]

## Tec family

**Table:** 

Nomenclature	BMX non‐receptor tyrosine kinase	Bruton tyrosine kinase	TXK tyrosine kinase
HGNC, UniProt	BMX, P51813	BTK, Q06187	TXK, P42681
EC number	2.7.10.2	2.7.10.2	2.7.10.2
Common abreviation	Etk	Btk	TXK
Inhibitors	compound 38 (pIC_50_ 9.1) [300], ibrutinib (pIC_50_ 9.1) [318], compound 31 (pIC_50_ 8.7) [300]	ibrutinib (pIC_50_ 9.3) [395], compound 31 (pIC_50_ 8.4) [300], compound 38 (pIC_50_>8.4) [300]	–
Selective inhibitors	BMX‐IN‐1 (pIC_50_ 8.1) [307]	CGI1746 (pIC_50_ 8.7) [120], CHMFL‐BTK‐11 (Irreversible inhibition) (pIC_50_ 7.6) [576]	–

## RAF family

**Table:** 

Nomenclature	B‐Raf proto‐oncogene, serine/threonine kinase	Raf‐1 proto‐oncogene, serine/threonine kinase
HGNC, UniProt	BRAF, P15056	RAF1, P04049
EC number	2.7.11.1	2.7.11.1
Common abreviation	B‐Raf	c‐Raf
Inhibitors	GDC‐0879 (pIC_50_ 9.7–9.9) [112, 206], dabrafenib (pIC_50_ 8.5) [305], regorafenib (pIC_50_ 7.6) [594], vemurafenib (pIC_50_ 7) [555], PLX‐4720 (p*K*_d_ 6.5) [112], compound 2 (p*K*_d_ 6.3) [227], CHIR‐265 (p*K*_d_ 5.9) [112]	–
Selective inhibitors	–	GW5074 (pIC_50_ 8.1) [88]

### Further reading on Kinases (EC 2.7.x.x)

Eglen R et al. (2011) Drug discovery and the human kinome: recent trends. Pharmacol. Ther. 130: 144‐56 [PMID:21256157]

Graves LM et al. (2013) The dynamic nature of the kinome. Biochem. J. 450: 1‐8 [PMID:23343193]

Liu Q et al. (2013) Developing irreversible inhibitors of the protein kinase cysteinome. Chem. Biol. 20: 146‐59 [PMID:23438744]

Martin KJ et al. (2012) Selective kinase inhibitors as tools for neuroscience research. Neuropharmacology 63: 1227‐37 [PMID:22846224]

Tarrant MK et al. (2009) The chemical biology of protein phosphorylation. Annu. Rev. Biochem. 78: 797‐825 [PMID:19489734]

Wu‐Zhang AX et al. (2013) Protein kinase C pharmacology: refining the toolbox. Biochem. J. 452: 195‐209 [PMID:23662807]

## Peptidases and proteinases

### Overview

Peptidases and proteinases hydrolyse peptide bonds, and can be simply divided on the basis of whether terminal peptide bonds are cleaved (exopeptidases and exoproteinases) at the amino terminus (aminopeptidases) or carboxy terminus (carboxypeptidases). Non‐terminal peptide bonds are cleaved by endopeptidases and endoproteinases, which are divided into serine endopeptidases (EC 3.4.21.‐), cysteine endopeptidases (EC 3.4.22.‐), aspartate endopeptidases (EC 3.4.23.‐), metalloendopeptidases (EC 3.4.24.‐) and threonine endopeptidases (EC 3.4.25.‐).

Since it is beyond the scope of the Guide to list all peptidase and proteinase activities, this summary focuses on selected enzymes of significant pharmacological interest that have ligands (mostly small‐molecules) directed against them. For those interested in detailed background we recommend the MEROPS database [450] (with whom we collaborate) as an information resource [432].

## A1: Pepsin

**Table:** 

Nomenclature	renin
HGNC, UniProt	REN, P00797
EC number	3.4.23.15
Inhibitors	aliskiren (pIC_50_ 9.2) [580]

## A22: Presenilin

### Overview

Presenilin (PS)‐1 or ‐2 act as the catalytic component/essential co‐factor of the *γ*‐secretase complex responsible for the final carboxy‐terminal cleavage of amyloid precursor protein (APP) [260] in the generation of amyloid beta (A*β*) [7, 510]. Given that the accumulation and aggregation of A*β* in the brain is pivotal in the development of Alzheimer's disease (AD), inhibition of PS activity is one mechanism being investigated as a therapeutic option for AD [187]. Several small molecule inhibitors of PS‐1 have been investigated, with some reaching early clinical trials, but none have been formally approved. Dewji et al. (2015) have reported that small peptide fragments of human PS‐1 can significantly inhibit A*β* production (total A*β*, A*β*40 and A*β*42) both in vitro and when infused in to the brains of APP transgenic mice [119]. The most active small peptides in this report were P4 and P8, from the amino‐terminal domain of PS‐1.

Information on members of this family may be found in the online database.

## C14: Caspase

### Overview

Caspases, (E.C. 3.4.22.‐) which derive their name from Cysteine ASPartate‐specific proteASES, include at least two families; initiator caspases (caspases 2, 8, 9 and 10), which are able to hydrolyse and activate a second family of effector caspases (caspases 3, 6 and 7), which themselves are able to hydrolyse further cellular proteins to bring about programmed cell death. Caspases are heterotetrameric, being made up of two pairs of subunits, generated by a single gene product, which is proteolysed to form the mature protein. Members of the mammalian inhibitors of apoptosis proteins (IAP) are able to bind the procaspases, thereby preventing maturation to active proteinases.

Information on members of this family may be found in the online database.

### Comments

CARD16 (Caspase recruitment domain‐containing protein 16, caspase‐1 inhibitor COP, CARD only domain‐containing protein 1, pseudo interleukin‐1*β* converting enzyme, pseudo‐ICE, ENSG00000204397) shares sequence similarity with some of the caspases.

## M1: Aminopeptidase N

### Overview

Aminopeptidases catalyze the cleavage of amino acids from the amino (N) terminus of protein or peptide substrates, and are involved in many essential cellular functions. Members of this enzyme family may be monomeric or multi‐subunit complexes, and many are zinc metalloenzymes [522].

Information on members of this family may be found in the online database.

## M2: Angiotensin‐converting (ACE and ACE2)

**Table:** 

Nomenclature	Angiotensin‐converting enzyme
HGNC, UniProt	ACE, P12821
EC number	3.4.15.1
Common abreviation	ACE
Endogenous substrates	angiotensin I (AGT, P01019) >angiotensin II (AGT, P01019)
Inhibitors	zofenoprilat (p*K*_i_ 9.4) [283] – Rabbit, captopril (p*K*_i_ 8.4) [354], zofenopril
Selective inhibitors	perindoprilat (pIC_50_ 9) [73], cilazaprilat (pIC_50_ 8.7) [559] – Rabbit, imidaprilat (pIC_50_ 8.7) [443], lisinopril‐tryptophan (C‐domain assay) (pIC_50_ 8.2) [560], RXP‐407 (N‐domain selective inhibition) (pIC_50_ 8.1) [472], fosinoprilat (pIC_50_ 8) [113] – Rabbit, enalaprilat (pIC_50_ 7.5) [87], benazeprilat (pIC_50_ 6.6) [296]
Comments	Reports of ACE GPI hydrolase activity [277] have been refuted [298]

## M10: Matrix metallopeptidase

### Overview

Matrix metalloproteinases (MMP) are calcium‐ and zinc‐dependent proteinases regulating the extracellular matrix and are often divided (e.g. [545]) on functional and structural bases into gelatinases, collagenases, stromyelinases and matrilysins, as well as membrane type‐MMP (MT‐MMP).

**Table:** 

Nomenclature	MMP2	MMP8
HGNC, UniProt	MMP2, P08253	MMP8, P22894
EC number	3.4.24.24	3.4.24.34
Selective inhibitors	ARP100 [537]	–
Comments	MMP2 is categorised as a gelatinase with substrate specificity for gelatinase A.	MMP8 is categorised as a collagenase.

### Comments

A number of small molecule ‘broad spectrum’ inhibitors of MMP have been described, including marimastat and batimastat.

Tissue inhibitors of metalloproteinase (TIMP) proteins are endogenous inhibitors acting to chelate MMP proteins: TIMP1(TIMP1, P01033), TIMP2(TIMP2, P16035), TIMP3(TIMP3, P35625), TIMP4(TIMP4, Q99727)

## M12: Astacin/Adamalysin

### Overview

ADAM (A Disintegrin And Metalloproteinase domain containing proteins) metalloproteinases cleave cell‐surface or transmembrane proteins to generate soluble and membrane‐limited products.

ADAMTS (with thrombospondin motifs) metalloproteinases cleave cell‐surface or transmembrane proteins to generate soluble and membrane‐limited products.

Information on members of this family may be found in the online database.

### Comments

Additional ADAM family members include AC123767.2 (cDNA FLJ58962, moderately similar to mouse ADAM3, ENSG00000231168), AL160191.3 (ADAM21‐like protein, ENSG00000235812), AC136428.3‐2 (ENSG00000185520) and ADAMDEC1 (decysin 1, ENSG00000134028).

Other ADAMTS family members include AC104758.12‐5 (FLJ00317 protein Fragment ENSG00000231463), AC139425.3‐1 (ENSG00000225577), and AC126339.6‐1 (ENSG00000225734).

## M28: Aminopeptidase Y

**Table:** 

Nomenclature	Folate hydrolase (prostate‐specific membrane antigen) 1
HGNC, UniProt	FOLH1, Q04609
EC number	3.4.17.21
Antibodies	capromab (Binding)
Comments	Folate hydrolase is also known as NAALADase as it is responsible for the hydrolysis of N‐acetaspartylglutamate to form N‐acetylaspartate and L‐glutamate (L‐glutamic acid). In the gut, the enzyme assists in the assimilation of folate by hydrolysing dietary poly‐gamma‐glutamylfolate. The enzyme is highly expressed in the prostate, and its expression is up‐regulated in cancerous tissue. A tagged version of the antibody capromab has been used for imaging purposes.

### Comments

Folate hydrolase is also known as NAALADase as it is responsible for the hydrolysis of N‐acetaspartylglutamate to form N‐acetylaspartate and L‐glutamate. In the gut, the enzyme assists in the assimilation of folate by hydrolysing dietary poly‐gamma‐glutamylfolate. The enzyme is highly expressed in the prostate, and its expression is up‐regulated in cancerous tissue. A tagged version of the antibody capromab has been used for imaging purposes.

## M19: Membrane dipeptidase

**Table:** 

Nomenclature	Dipeptidase 1
HGNC, UniProt	DPEP1, P16444
EC number	3.4.13.19: LTD_4_ + H_2_O = LTE_4_ + glycine
Inhibitors	cilastatin (p*K*_i_ 6) [189]

## S1: Chymotrypsin

**Table:** 

Nomenclature	complement C1r	coagulation factor II, thrombin	coagulation factor X
HGNC, UniProt	C1R, P00736	F2, P00734	F10, P00742
EC number	3.4.21.41	3.4.21.5	3.4.21.6
Inhibitors	nafamostat (pIC_50_ 4.9) [216]	lepirudin (p*K*_i_ 13) [506], desirudin (p*K*_i_ 12.7) [254], AZ12971554 (p*K*_i_ 9.5) [19], melagatran (p*K*_i_ 8.7) [198], bivalirudin (p*K*_i_ 8.6) [573], dabigatran (p*K*_i_ 8.3) [211], argatroban (p*K*_i_ 7.7) [238]	rivaroxaban (p*K*_i_ 9.4) [407], edoxaban (p*K*_i_ 9.2) [412], apixaban (p*K*_i_ 9.1) [574]
Selective inhibitors	–	Dup‐714 (p*K*_i_ 10.4) [175], AR‐H067637 (pIC_50_ 8.4) [114]	–

**Table:** 

Nomenclature	elastase, neutrophil expressed	plasminogen	plasminogen activator, tissue type	protease, serine 1	tryptase alpha/beta 1
HGNC, UniProt	ELANE, P08246	PLG, P00747	PLAT, P00750	PRSS1, P07477	TPSAB1, Q15661
EC number	3.4.21.37	3.4.21.7	3.4.21.68	3.4.21.4	3.4.21.59
Inhibitors	alvelestat (p*K*_i_ 8) [502], sivelestat (pIC_50_ 7.4) [103]	aprotinin {Bovine} (Binding) (pIC_50_ 6.8) [492], tranexamic acid (Binding) (pIC_50_ 3.6) [492]	–	nafamostat (pIC_50_ 7.8) [216]	nafamostat (pIC_50_ 10) [365]
Selective inhibitors	–	6‐aminocaproic acid (Binding) (pIC_50_ 4.4) [86]	–	–	gabexate (pIC_50_ 8.5) [135]

## T1: Proteasome

### Overview

The T1 macropain beta subunits form the catalytic proteinase core of the 20S proteasome complex [93]. This catalytic core enables the degradation of peptides with Arg, Phe, Tyr, Leu, and Glu adjacent to the cleavage site. The *β*5 subunit is the principal target of the approved drug proteasome inhibitor bortezomib.

**Table:** 

Nomenclature	proteasome subunit beta 5
HGNC, UniProt	PSMB5, P28074
EC number	3.4.25.1
Inhibitors	bortezomib (pIC_50_ 7.7) [371]
Selective inhibitors	ixazomib (p*K*_i_ 9) [286]

## S8: Subtilisin

### Overview

One member of this family has garnered intense interest as a clinical drug target. As liver PCSK9 acts to maintain cholesterol homeostasis, it has become a target of intense interest for clinical drug development. Inhibition of PCSK9 can lower low‐density cholesterol (LDL‐C) by clearing LDLR‐bound LDL particles, thereby lowering circulating cholesterol levels. It is hypothesised that this action may improve outcomes in patients with atherosclerotic cardiovascular disease [315, 452, 501]. Therapeutics which inhibit PCSK9 are viewed as potentially lucrative replacements for statins, upon statin patent expiry. Several monoclonal antibodies including alirocumab, evolocumab, bococizumab, RG‐7652 and LY3015014 are under development. One RNAi therapeutic, code named ALN‐PCS02, is also in development [106, 147, 155].

Information on members of this family may be found in the online database.

## S9: Prolyl oligopeptidase

**Table:** 

Nomenclature	dipeptidyl peptidase 4
HGNC, UniProt	DPP4, P27487
EC number	3.4.14.5
Endogenous substrates	glucagon‐like peptide 1 (GCG, P01275)
Inhibitors	saxagliptin (p*K*_i_ 9.2) [196], linagliptin (p*K*_i_ 9) [130], sitagliptin (pIC_50_ 8.1) [111], vildagliptin (p*K*_i_ 7.8) [196]

## Acetylcholine turnover

### Overview

Acetylcholine is familiar as a neurotransmitter in the central nervous system and in the periphery. In the somatic nervous system, it activates nicotinic acetylcholine receptors at the skeletal neuromuscular junction. It is also employed in the autonomic nervous system, in both parasympathetic and sympathetic branches; in the former, at the smooth muscle neuromuscular junction, activating muscarinic acetylcholine receptors. In the latter, acetylcholine is involved as a neurotransmitter at the ganglion, activating nicotinic acetylcholine receptors. Acetylcholine is synthesised in neurones through the action of choline O‐acetyltransferase and metabolised after release through the extracellular action of acetylcholinesterase and cholinesterase. Choline is accumulated from the extracellular medium by selective transporters (see SLC5A7 and the SLC44 family). Acetylcholine is accumulated in synaptic vesicles through the action of the vesicular acetylcholine transporter SLC18A3.

**Table:** 

Nomenclature	choline O‐acetyltransferase	acetylcholinesterase (Cartwright blood group)	butyrylcholinesterase
HGNC, UniProt	CHAT, P28329	ACHE, P22303	BCHE, P06276
EC number	2.3.1.6: acetyl CoA + choline = acetylcholine + coenzyme A	3.1.1.7: acetylcholine + H_2_O = acetic acid + choline + H^+^	3.1.1.7: acetylcholine + H_2_O = acetic acid + choline + H^+^
Common abreviation	ChAT	AChE	BChE
Inhibitors	compound 2 (pIC_50_ 6.5) [190] – Mouse	tacrine (p*K*_i_ 7.5) [58], galantamine (pIC_50_ 6.3) [94], rivastigmine (pIC_50_ 5.4) [325]	rivastigmine (pIC_50_ 7.4) [325], tacrine (p*K*_i_ 7.2) [58]
Sub/family‐selective inhibitors	–	physostigmine (pIC_50_ 7.6–7.8) [325]	physostigmine (pIC_50_ 7.6–7.8) [325]
Selective inhibitors	–	donepezil (pIC_50_ 7.7–8.3) [68, 170, 325], BW284C51 (pIC_50_ 7.7) [182]	bambuterol (pIC_50_ 8.5) [182]
Comments	Splice variants of choline O‐acetyltransferase are suggested to be differentially distributed in the periphery and CNS (see [30]).	–	–

### Comments

A number of organophosphorus compounds inhibit acetylcholinesterase and cholinesterase irreversibly, including pesticides such as chlorpyrifos‐oxon, and nerve agents such as tabun, soman and sarin. AChE is unusual in its exceptionally high turnover rate which has been calculated at 740 000/min/molecule [570].

### Further reading on Acetylcholine turnover

Li Q et al. (2017) Recent progress in the identification of selective butyrylcholinesterase inhibitors for Alzheimer's disease. Eur J Med Chem 132: 294‐309 [PMID:28371641]

Lockridge O. (2015) Review of human butyrylcholinesterase structure, function, genetic variants, history of use in the clinic, and potential therapeutic uses. Pharmacol Ther 148: 34‐46 [PMID:25448037]

Masson P et al. (2016) Slow‐binding inhibition of cholinesterases, pharmacological and toxicological relevance. Arch Biochem Biophys 593: 60‐8 [PMID:26874196]

Rotundo RL. (2017) Biogenesis, assembly and trafficking of acetylcholinesterase. J Neurochem [PMID:28326552]

Silman I et al. (2017) Recent developments in structural studies on acetylcholinesterase. J Neurochem [PMID:28503857]

## Adenosine turnover

### Overview

A multifunctional, ubiquitous molecule, adenosine acts at cell‐surface G protein‐coupled receptors, as well as numerous enzymes, including protein kinases and adenylyl cyclase. Extracellular adenosine is thought to be produced either by export or by metabolism, predominantly through ecto‐5'‐nucleotidase activity (also producing inorganic phosphate). It is inactivated either by extracellular metabolism via adenosine deaminase (also producing ammonia) or, following uptake by nucleoside transporters, via adenosine deaminase or adenosine kinase (requiring ATP as co‐substrate). Intracellular adenosine may be produced by cytosolic 5'‐nucleotidases or through S‐adenosylhomocysteine hydrolase (also producing L‐homocysteine).

**Table:** 

Nomenclature	Adenosine deaminase	Adenosine kinase	Ecto‐5'‐Nucleotidase	S‐Adenosylhomocysteine hydrolase
Systematic nomenclature	–	–	CD73	–
HGNC, UniProt	ADA, P00813	ADK, P55263	NT5E, P21589	AHCY, P23526
EC number	3.5.4.4: adenosine + H_2_O = inosine + NH_3_	2.7.1.20	3.1.3.5	3.3.1.1
Common abreviation	ADA	ADK	NT5E	SAHH
Rank order of affinity	2'‐deoxyadenosine>adenosine	adenosine	adenosine 5'‐monophosphate, 5'‐GMP, 5'‐inosine monophosphate, 5'‐UMP>5'‐dAMP, 5'‐dGMP	–
Endogenous substrates	–	–	–	S‐adenosylhomocysteine
Products	2'‐deoxyinosine, inosine	adenosine 5'‐monophosphate	uridine, inosine, guanine, adenosine	adenosine
Inhibitors	–	–	–	DZNep (p*K*_i_ 12.3) [184] – Hamster
Selective inhibitors	pentostatin (pIC_50_ 10.8) [4], EHNA (p*K*_i_ 8.8) [4]	A134974 (pIC_50_ 10.2) [348], ABT702 (pIC_50_ 8.8) [248]	*αβ*‐methyleneADP (pIC_50_ 8.7) [56]	3‐deazaadenosine (pIC_50_ 8.5) [197]
Comments	–	The enzyme exists in two isoforms derived from alternative splicing of a single gene product: a short isoform, ADK‐S, located in the cytoplasm is responsible for the regulation of intra‐ and extracellular levels of adenosine and hence adenosine receptor activation; a long isoform, ADK‐L, located in the nucleus contributes to the regulation of DNA methylation [48, 569].	Pharmacological inhibition of CD73 is being investigated as a novel cancer immunotherapy strategy [552].	–

### Comments

An extracellular adenosine deaminase activity, termed ADA2 or adenosine deaminase growth factor (ADGF, CECR1, Q9NZK5) has been identified [101, 331], which is insensitive to EHNA[595]. Other forms of adenosine deaminase act on ribonucleic acids and may be divided into two families: ADAT1(Q9BUB4) deaminates transfer RNA; ADAR(EC 3.5.4.37, also known as 136 kDa double‐stranded RNA‐binding protein, P136, K88DSRBP, Interferon‐inducible protein 4); ADARB1 (EC 3.5.‐.‐, , also known as dsRNA adenosine deaminase) and ADARB2 (EC 3.5.‐.‐, also known as dsRNA adenosine deaminase B2, RNA‐dependent adenosine deaminase 3) act on double‐stranded RNA. Particular polymorphisms of the ADA gene result in loss‐of‐function and severe combined immunodeficiency syndrome. Adenosine deaminase is able to complex with dipeptidyl peptidase IV (EC 3.4.14.5, DPP4, also known as T‐cell activation antigen CD26, TP103, adenosine deaminase complexing protein 2) to form a cell‐surface activity [259].

### Further reading on Adenosine turnover

Boison D. (2013) Adenosine kinase: exploitation for therapeutic gain. Pharmacol. Rev. 65: 906‐43 [PMID:23592612]

Cortés A et al. (2015) Moonlighting adenosine deaminase: a target protein for drug development. Med Res Rev 35: 85‐125 [PMID:24933472]

Nishikura K (2016) A‐to‐I editing of coding and non‐coding RNAs by ADARs. Nat Rev Mol Cell Biol 17: 83‐96 [PMID:26648264]

Sawynok J (2016) Adenosine receptor targets for pain. Neuroscience 338: 1‐18 [PMID:26500181]

Xiao Y et al. (2015) Role of S‐adenosylhomocysteine in cardiovascular disease and its potential epigenetic mechanism. Int J Biochem Cell Biol 67: 158‐66 [PMID:26117455]

## Amino acid hydroxylases

### Overview

The amino acid hydroxylases (monooxygenases), EC.1.14.16.‐, are iron‐containing enzymes which utilise molecular oxygen and sapropterin as co‐substrate and co‐factor, respectively. In humans, as well as in other mammals, there are two distinct L‐Tryptophan hydroxylase 2 genes. In humans, these genes are located on chromosomes 11 and 12 and encode two different homologous enzymes, TPH1 and TPH2.

**Table:** 

Nomenclature	L‐Phenylalanine hydroxylase	L‐Tyrosine hydroxylase	L‐Tryptophan hydroxylase 1	L‐Tryptophan hydroxylase 2
HGNC, UniProt	PAH, P00439	TH, P07101	TPH1, P17752	TPH2, Q8IWU9
EC number	1.14.16.1: L‐phenylalanine + O_2_ ‐>L‐tyrosine	1.14.16.2: L‐tyrosine + O_2_ ‐>levodopa	1.14.16.4	1.14.16.4
Endogenous substrates	L‐phenylalanine	L‐tyrosine	L‐tryptophan	L‐tryptophan
Products	L‐tyrosine	levodopa	5‐hydroxy‐L‐tryptophan	5‐hydroxy‐L‐tryptophan
Cofactors	sapropterin	sapropterin, Fe^2+^	–	–
Endogenous activators	Protein kinase A‐mediated phosphorylation (Rat) [2]	Protein kinase A‐mediated phosphorylation [251]	Protein kinase A‐mediated phosphorylation [252]	Protein kinase A‐mediated phosphorylation [252]
Inhibitors	–	methyltyrosine	telotristat ethyl [267]	–
Selective inhibitors	*α*‐methylphenylalanine [191] – Rat, fenclonine	*α*‐propyldopacetamide, 3‐chlorotyrosine, 3‐iodotyrosine, alpha‐methyltyrosine	*α*‐propyldopacetamide, 6‐fluorotryptophan [377], fenclonine, fenfluramine	*α*‐propyldopacetamide, 6‐fluorotryptophan [377], fenclonine, fenfluramine
Comments	PAH is an iron bound homodimer or ‐tetramer from the same structural family as tyrosine 3‐monooxygenase and the tryptophan hydroxylases. Deficiency or loss‐of‐function of PAH is associated with phenylketonuria	TH is a homotetramer, which is inhibited by dopamine and other catecholamines in a physiological negative feedback pathway [109].	–	–

### Further reading on Amino acid hydroxylases

Bauer IE et al. (2015) Serotonergic gene variation in substance use pharmacotherapy: a systematic review. Pharmacogenomics 16: 1307‐14 [PMID:26265436]

Daubner SC et al. (2011) Tyrosine hydroxylase and regulation of dopamine synthesis. Arch. Biochem. Biophys. 508: 1‐12 [PMID:21176768]

Flydal MI et al. (2013) Phenylalanine hydroxylase: function, structure, and regulation. IUBMB Life 65: 341‐9 [PMID:23457044]

Roberts KM et al. (2013) Mechanisms of tryptophan and tyrosine hydroxylase. IUBMB Life 65: 350‐7 [PMID:23441081]

Tekin I et al. (2014) Complex molecular regulation of tyrosine hydroxylase. J Neural Transm 121: 1451‐81 [PMID:24866693]

Waloen K et al. (2017) Tyrosine and tryptophan hydroxylases as therapeutic targets in human disease. Expert Opin Ther Targets 21: 167‐180 [PMID:27973928]

## L‐Arginine turnover

### Overview

L‐arginine is a basic amino acid with a guanidino sidechain. As an amino acid, metabolism of L‐arginine to form L‐ornithine, catalysed by arginase, forms the last step of the urea production cycle. L‐Ornithine may be utilised as a precursor of polyamines (see Carboxylases and Decarboxylases) or recycled via L‐argininosuccinic acid to L‐arginine. L‐Arginine may itself be decarboxylated to form agmatine, although the prominence of this pathway in human tissues is uncertain. L‐Arginine may be used as a precursor for guanidoacetic acid formation in the creatine synthesis pathway under the influence of arginine:glycine amidinotransferase with L‐ornithine as a byproduct. Nitric oxide synthase uses L‐arginine to generate nitric oxide, with L‐citrulline also as a byproduct.

L‐Arginine in proteins may be subject to post‐translational modification through methylation, catalysed by protein arginine methyltransferases. Subsequent proteolysis can liberate asymmetric N^G^,N^G^‐dimethyl‐L‐arginine(ADMA), which is an endogenous inhibitor of nitric oxide synthase activities. ADMA is hydrolysed by dimethylarginine dimethylhydrolase activities to generate L‐citrulline and dimethylamine.

### Further reading on L‐Arginine turnover

Lai L et al. (2016) Modulating DDAH/NOS Pathway to Discover Vasoprotective Insulin Sensitizers. J Diabetes Res 2016: 1982096 [PMID:26770984]

Pekarova M et al. (2015) The crucial role of l‐arginine in macrophage activation: What you need to know about it. Life Sci 137: 44‐8 [PMID:26188591]

Pudlo M et al. (2017) Arginase Inhibitors: A Rational Approach Over One Century. Med Res Rev 37: 475‐513 [PMID:27862081]

Sudar‐Milovanovic E et al. (2016) Benefits of L‐Arginine on Cardiovascular. System Mini Rev Med Chem 16: 94‐103 [PMID:26471966]

## 2.1.1.‐ Protein arginine N‐methyltransferases

### Overview

Protein arginine N‐methyltransferases (PRMT, EC 2.1.1.‐) encompass histone arginine N‐methyltransferases (PRMT4, PRMT7, EC 2.1.1.125) and myelin basic protein N‐methyltransferases (PRMT7, EC 2.1.1.126). They are dimeric or tetrameric enzymes which use S‐adenosyl methionine as a methyl donor, generating S‐adenosylhomocysteine as a by‐product. They generate both mono‐methylated and di‐methylated products; these may be symmetric (SDMA) or asymmetric (N^G^,N^G^‐dimethyl‐L‐arginine) versions, where both guanidine nitrogens are monomethylated or one of the two is dimethylated, respectively.

Information on members of this family may be found in the online database.

## Arginase

### Overview

Arginase (EC 3.5.3.1) are manganese‐containing isoforms, which appear to show differential distribution, where the ARG1 isoform predominates in the liver and erythrocytes, while ARG2 is associated more with the kidney.

Information on members of this family may be found in the online database.

### Comments

N^*ω*^‐hydroxyarginine, an intermediate in NOS metabolism of L‐arginine acts as a weak inhibitor and may function as a physiological regulator of arginase activity. Although isoform‐selective inhibitors of arginase are not available, examples of inhibitors selective for arginase compared to NOS are N^*ω*^‐hydroxy‐nor‐L‐arginine[525], S‐(2‐boronoethyl)‐L‐cysteine[97, 268] and 2(S)‐amino‐6‐boronohexanoic acid [24, 97].

## Arginine:glycine amidinotransferase

**Table:** 

Nomenclature	Arginine:glycine amidinotransferase
HGNC, UniProt	GATM, P50440
EC number	2.1.4.1
Common abreviation	AGAT

## Dimethylarginine dimethylaminohydrolases

### Overview

Dimethylarginine dimethylaminohydrolases (DDAH, EC 3.5.3.18) are cytoplasmic enzymes which hydrolyse N^G^,N^G^‐dimethyl‐L‐arginine to form dimethylamine and L‐citrulline.

**Table:** 

Nomenclature	N^G^,N^G^‐Dimethylarginine dimethylaminohydrolase 1	N^G^,N^G^‐Dimethylarginine dimethylaminohydrolase 2
HGNC, UniProt	DDAH1, O94760	DDAH2, O95865
EC number	3.5.3.18	3.5.3.18
Common abreviation	DDAH1	DDAH2
Cofactors	Zn^2+^	–
Inhibitors	compound 2e (p*K*_i_ 5.7) [279]	–

## Nitric oxide synthases

### Overview

Nitric oxide synthases (NOS, E.C. 1.14.13.39) are a family of oxidoreductases that synthesize nitric oxide (NO.) via the NADPH and oxygen‐dependent consumption of L‐arginine with the resultant by‐product, L‐citrulline. There are 3 NOS isoforms and they are related by their capacity to produce NO, highly conserved organization of functional domains and significant homology at the amino acid level. NOS isoforms are functionally distinguished by the cell type where they are expressed, intracellular targeting and transcriptional and post‐translation mechanisms regulating enzyme activity. The nomenclature suggested by NC‐IUPHAR of NOS I, II and III [363] has not gained wide acceptance, and the 3 isoforms are more commonly referred to as neuronal NOS (nNOS), inducible NOS (iNOS) and endothelial NOS (eNOS) which reflect the location of expression (nNOS and eNOS) and inducible expression (iNOS). All are dimeric enzymes that shuttle electrons from NADPH, which binds to a C‐terminal reductase domain, through the flavins FAD and FMN to the oxygenase domain of the other monomer to enable the BH4‐dependent reduction of heme bound oxygen for insertion into the substrate, L‐arginine. Electron flow from reductase to oxygenase domain is controlled by calmodulin binding to canonical calmodulin binding motif located between these domains. eNOS and nNOS isoforms are activated at concentrations of calcium greater than 100 nM, while iNOS shows higher affinity for Ca^2+^/calmodulin(CALM1 CALM2 CALM3, P62158) with great avidity and is essentially calcium‐independent and constitutively active. Efficient stimulus‐dependent coupling of nNOS and eNOS is achieved via subcellular targeting through respective N‐terminal PDZ and fatty acid acylation domains whereas iNOS is largely cytosolic and function is independent of intracellular location. nNOS is primarily expressed in the brain and neuronal tissue, iNOS in immune cells such as macrophages and eNOS in the endothelial layer of the vasculature although exceptions in other cells have been documented. L‐NAME and related modified arginine analogues are inhibitors of all three isoforms, with IC_50_ values in the micromolar range.

**Table:** 

Nomenclature	Endothelial NOS	Inducible NOS	Neuronal NOS
HGNC, UniProt	NOS3, P29474	NOS2, P35228	NOS1, P29475
EC number	1.14.13.39	1.14.13.39	1.14.13.39
Common abreviation	eNOS	iNOS	nNOS
Endogenous Substrate	L‐arginine	L‐arginine	L‐arginine
Products	NO, L‐citrulline	NO, L‐citrulline	L‐citrulline, NO
Cofactors	oxygen, BH4, Zn^2+^, flavin mononucleotide, NADPH, heme, flavin adenine dinucleotide	heme, flavin mononucleotide, flavin adenine dinucleotide, oxygen, NADPH, Zn^2+^, BH4	flavin adenine dinucleotide, heme, oxygen, BH4, flavin mononucleotide, NADPH, Zn^2+^
Selective inhibitors	–	1400W (pIC_50_ 8.2) [178], 2‐amino‐4‐methylpyridine (pIC_50_ 7.4) [139], PIBTU (pIC_50_ 7.3) [179], NIL (pIC_50_ 5.5) [364], aminoguanidine [99]	3‐bromo‐7NI (pIC_50_ 6.1–6.5) [43], 7NI (pIC_50_ 5.3) [20]

### Comments

The reductase domain of NOS catalyses the reduction of cytochrome c and other redox‐active dyes [345]. NADPH:O_2_ oxidoreductase catalyses the formation of superoxide anion/H_2_O_2_ in the absence of L‐arginine and sapropterin.

### Further reading on Nitric oxide synthases

Bogdan, C. (2015) Nitric oxide synthase in innate and adaptive immunity: An update. Trends Immunol 36: 161‐78 [PMID:25687683]

Lundberg JO et al. (2015) Strategies to increase nitric oxide signalling in cardiovascular disease. Nat Rev Drug Discov 14: 623‐41 [PMID:26265312]

Oliveira‐Paula GH et al. (2016) Endothelial nitric oxide synthase: From biochemistry and gene structure to clinical implications of NOS3 polymorphisms. Gene 575: 584‐99 [PMID:26428312]

Shu X et al. (2015) Endothelial nitric oxide synthase in the microcirculation. Cell Mol Life Sci 72: 4561‐75 [PMID:26390975]

Zhao Y et al. (2015) Vascular nitric oxide: Beyond eNOS. J Pharmacol Sci 129: 83‐94 [PMID:26499181]

## Carboxylases and decarboxylases

## Carboxylases

### Overview

The carboxylases allow the production of new carbon‐carbon bonds by introducing HCO_3_^‐^ or CO_2_ into target molecules. Two groups of carboxylase activities, some of which are bidirectional, can be defined on the basis of the cofactor requirement, making use of biotin (EC 6.4.1.‐) or vitamin K hydroquinone (EC 4.1.1.‐).

**Table:** 

Nomenclature	Pyruvate carboxylase	Acetyl‐CoA carboxylase 1	Acetyl‐CoA carboxylase 2	Propionyl‐CoA carboxylase	*γ*‐Glutamyl carboxylase
HGNC, UniProt	PC, P11498	ACACA, Q13085	ACACB, O00763	–	GGCX, P38435
Subunits	–	–	–	Propionyl‐CoA carboxylase*β* subunit, Propionyl‐CoA carboxylase *α* subunit	–
EC number	6.4.1.1	6.4.1.2	6.4.1.2	6.4.1.3	4.1.1.90
Common abreviation	PC	ACC1	ACC2	PCCA,PCCB	GGCX
Endogenous substrates	ATP, pyruvic acid	ATP, acetyl CoA	acetyl CoA, ATP	propionyl‐CoA, ATP	glutamyl peptides
Products	P_i_, ADP, oxalacetic acid	P_i_, ADP, malonyl‐CoA	P_i_, ADP, malonyl‐CoA	ADP, methylmalonyl‐CoA, P_i_	carboxyglutamyl peptides
Cofactors	biotin	biotin	biotin	biotin	vitamin K hydroquinone, NADPH
Inhibitors	–	–	–	–	anisindione
Selective inhibitors	–	TOFA (pIC_50_ 4.9) [599]	TOFA (pIC_50_ 4.9) [599]	–	–
Comments	–	Citrate and other dicarboxylic acids are allosteric activators of acetyl‐CoA carboxylase.		Propionyl‐CoA carboxylase is able to function in both forward and reverse activity modes, as a ligase (carboxylase) or lyase (decarboxylase), respectively.	Loss‐of‐function mutations in *γ*‐glutamyl carboxylase are associated with clotting disorders.

### Comments

Dicarboxylic acids including citric acid are able to activate ACC1/ACC2 activity allosterically. PCC is able to function in forward and reverse modes as a ligase (carboxylase) or lyase (decarboxylase) activity, respectively. Loss‐of‐function mutations in GGCX are associated with clotting disorders.

## Decarboxylases

### Overview

The decarboxylases generate CO_2_ and the indicated products from acidic substrates, requiring pyridoxal phosphate or pyruvic acid as a co‐factor.

**Table:** 

Nomenclature	Glutamic acid decarboxylase 1	Glutamic acid decarboxylase 2	Histidine decarboxylase
HGNC, UniProt	GAD1, Q99259	GAD2, Q05329	HDC, P19113
EC number	4.1.1.15: L‐glutamic acid + H^+^ ‐>GABA + CO_2_	4.1.1.15: L‐glutamic acid + H^+^ ‐>GABA + CO_2_	4.1.1.22
Common abreviation	GAD1	GAD2	HDC
Endogenous substrates	L‐glutamic acid, L‐aspartic acid	L‐glutamic acid, L‐aspartic acid	L‐histidine
Products	GABA	GABA	histamine
Cofactors	pyridoxal phosphate	pyridoxal phosphate	pyridoxal phosphate
Selective inhibitors	s‐allylglycine	s‐allylglycine	AMA, FMH [174]
Comments	L‐aspartic acid is a less rapidly metabolised substrate of mouse brain glutamic acid decarboxylase generating *β*‐alanine [577]. Autoantibodies against GAD1 and GAD2 are elevated in type 1 diabetes mellitus and neurological disorders (see Further reading).		–

**Table:** 

Nomenclature	L‐Arginine decarboxylase	L‐Aromatic amino‐acid decarboxylase	Malonyl‐CoA decarboxylase	Ornithine decarboxylase	Phosphatidylserine decarboxylase	S‐Adenosylmethionine decarboxylase
HGNC, UniProt	AZIN2, Q96A70	DDC, P20711	MLYCD, O95822	ODC1, P11926	PISD, Q9UG56	AMD1, P17707
EC number	4.1.1.19	4.1.1.28: levodopa ‐>dopamine + CO_2_ 5‐hydroxy‐L‐tryptophan ‐>5‐hydroxytryptamine + CO_2_ This enzyme also catalyses the following reaction:: L‐tryptophan ‐>tryptamine + CO_2_	4.1.1.9	4.1.1.17	4.1.1.65	4.1.1.50
Common abreviation	ADC	AADC	MLYCD	ODC	PSDC	SAMDC
Endogenous substrates	L‐arginine	levodopa, 5‐hydroxy‐L‐tryptophan, L‐tryptophan	malonyl‐CoA	L‐ornithine	phosphatidylserine	S‐adenosyl methionine
Products	agmatine [601]	5‐hydroxytryptamine, dopamine	acetyl CoA	putrescine	phosphatidylethanolamine	S‐adenosyl‐L‐ methioninamine
Cofactors	pyridoxal phosphate	pyridoxal phosphate	pyridoxal phosphate	pyridoxal phosphate	pyruvic acid	pyruvic acid
Selective inhibitors	–	3‐hydroxybenzylhydrazine, L‐*α*‐methyldopa, benserazide [108], carbidopa	–	APA (pIC_50_ 7.5) [494], eflornithine (p*K*_d_ 4.9) [422]	–	sardomozide (pIC_50_ 8) [493]
Comments	The presence of a functional ADC activity in human tissues has been questioned [96].	AADC is a homodimer.	Inhibited by AMP‐activated protein kinase‐evoked phosphorylation [451]	The activity of ODC is regulated by the presence of an antizyme (ENSG00000104904) and an ODC antizyme inhibitor (ENSG00000155096).	S‐allylglycine is also an inhibitor of SAMDC [393].	s‐allylglycine is also an inhibitor of SAMDC [393].

### Further reading on Carboxylases and decarboxylases

Bale S et al. (2010) Structural biology of S‐adenosylmethionine decarboxylase. Amino Acids 38: 451‐60 [PMID:19997761]

Jitrapakdee S et al. (2008) Structure, mechanism and regulation of pyruvate carboxylase. Biochem. J. 413: 369‐87 [PMID:18613815]

Lietzan AD et al. (2014) Functionally diverse biotin‐dependent enzymes with oxaloacetate decarboxylase activity. Arch. Biochem. Biophys. 544: 75‐86 [PMID:24184447]

Moya‐García AA et al. (2009) Structural features of mammalian histidine decarboxylase reveal the basis for specific inhibition. Br. J. Pharmacol. 157: 4‐13 [PMID:19413567]

Tong L. (2013) Structure and function of biotin‐dependent carboxylases. Cell. Mol. Life Sci. 70: 863‐91 [PMID:22869039]

Vance JE et al. (2013) Formation and function of phosphatidylserine and phosphatidylethanolamine in mammalian cells. Biochim. Biophys. Acta 1831: 543‐54 [PMID:22960354]

## Catecholamine turnover

### Overview

Catecholamines are defined by the presence of two adjacent hydroxyls on a benzene ring with a sidechain containing an amine. The predominant catacholamines in mammalian biology are the neurotransmitter/hormones dopamine, (‐)‐noradrenaline (norepinephrine) and (‐)‐adrenaline(epinephrine). These hormone/transmitters are synthesized by sequential metabolism from L‐phenylalanine via L‐tyrosine. Hydroxylation of L‐tyrosine generates levodopa, which is decarboxylated to form dopamine. Hydroxylation of the ethylamine sidechain generates (‐)‐noradrenaline (norepinephrine), which can be methylated to form (‐)‐adrenaline(epinephrine). In particular neuronal and adrenal chromaffin cells, the catecholamines dopamine, (‐)‐noradrenaline and (‐)‐adrenaline are accumulated into vesicles under the influence of the vesicular monoamine transporters (VMAT1/SLC18A1 and VMAT2/SLC18A2). After release into the synapse or the bloodstream, catecholamines are accumulated through the action cell‐surface transporters, primarily the dopamine (DAT/SLC6A3) and norepinephrine transporter (NET/SLC6A2). The primary routes of metabolism of these catecholamines are oxidation via monoamine oxidase activities of methylation via catechol O‐methyltransferase.

**Table:** 

Nomenclature	L‐Phenylalanine hydroxylase	Tyrosine aminotransferase	L‐Tyrosine hydroxylase	Dopamine beta‐hydroxylase (dopamine beta‐monooxygenase)	L‐Aromatic amino‐acid decarboxylase
HGNC, UniProt	PAH, P00439	TAT, P17735	TH, P07101	DBH, P09172	DDC, P20711
EC number	1.14.16.1: L‐phenylalanine + O_2_ ‐>L‐tyrosine	2.6.1.5: L‐tyrosine + *α*‐ketoglutaric acid ‐>4‐hydroxyphenylpyruvic acid + L‐glutamic acid	1.14.16.2: L‐tyrosine + O_2_ ‐>levodopa	1.14.17.1: dopamine + O_2_ = (‐)‐noradrenaline + H_2_O	4.1.1.28: levodopa ‐>dopamine + CO_2_ 5‐hydroxy‐L‐tryptophan ‐>5‐hydroxytryptamine + CO_2_ This enzyme also catalyses the following reaction:: L‐tryptophan ‐>tryptamine + CO_2_
Common abreviation	–	TAT	–	DBH	AADC
Endogenous substrates	L‐phenylalanine	–	L‐tyrosine	–	levodopa, 5‐hydroxy‐L‐tryptophan, L‐tryptophan
Products	L‐tyrosine	–	levodopa	–	5‐hydroxytryptamine, dopamine
Cofactors	sapropterin	pyridoxal phosphate	sapropterin, Fe^2+^	Cu^2+^, L‐ascorbic acid	pyridoxal phosphate
Endogenous activators	Protein kinase A‐mediated phosphorylation (Rat) [2]	–	Protein kinase A‐mediated phosphorylation [251]	–	–
Selective inhibitors	*α*‐methylphenylalanine [191] – Rat, fenclonine	–	*α*‐propyldopacetamide, 3‐chlorotyrosine, 3‐iodotyrosine, alpha‐methyltyrosine	nepicastat (pIC_50_ 8) [496]	3‐hydroxybenzylhydrazine, L‐*α*‐methyldopa, benserazide [108], carbidopa
Comments	PAH is an iron bound homodimer or ‐tetramer from the same structural family as tyrosine 3‐monooxygenase and the tryptophan hydroxylases. Deficiency or loss‐of‐function of PAH is associated with phenylketonuria	Tyrosine may also be metabolized in the liver by tyrosine transaminase to generate 4‐hydroxyphenylpyruvic acid, which can be further metabolized to homogentisic acid. TAT is a homodimer, where loss‐of‐function mutations are associated with type II tyrosinemia.	TH is a homotetramer, which is inhibited by dopamine and other catecholamines in a physiological negative feedback pathway [109].	DBH is a homotetramer. A protein structurally‐related to DBH (MOXD1, Q6UVY6) has been described and for which a function has yet to be identified [76].	AADC is a homodimer.

**Table:** 

Nomenclature	Phenylethanolamine N‐methyltransferase	Monoamine oxidase A	Monoamine oxidase B	Catechol‐O‐methyltransferase
HGNC, UniProt	PNMT, P11086	MAOA, P21397	MAOB, P27338	COMT, P21964
EC number	2.1.1.28: (‐)‐noradrenaline ‐>(‐)‐adrenaline	1.4.3.4 (‐)‐adrenaline ‐>3,4‐dihydroxymandelic acid + NH_3_ (‐)‐noradrenaline ‐>3,4‐dihydroxymandelic acid + NH_3_ tyramine ‐>4‐hydroxyphenyl acetaldehyde + NH_3_ dopamine ‐>3,4‐dihydroxyphenylacetaldehyde + NH_3_ 5‐hydroxytryptamine ‐>5‐hydroxyindole acetaldehyde + NH_3_	1.4.3.4	2.1.1.6: S‐adenosyl‐L‐methionine + a catechol = S‐adenosyl‐L‐homocysteine + a guaiacol (‐)‐noradrenaline ‐>normetanephrine dopamine ‐>3‐methoxytyramine 3,4‐dihydroxymandelic acid ‐>vanillylmandelic acid (‐)‐adrenaline ‐>metanephrine
Common abreviation	PNMT	MAO‐A	MAO‐B	COMT
Cofactors	S‐adenosyl methionine	flavin adenine dinucleotide	flavin adenine dinucleotide	S‐adenosyl methionine
Inhibitors	LY134046 (p*K*_i_ 7.6) [163]	moclobemide (p*K*_i_ 8.3) [247], phenelzine (Irreversible inhibition) (p*K*_i_ 7.3) [39], tranylcypromine (pIC_50_ 4.7) [587], selegiline (p*K*_i_ 4.2) [357], befloxatone [107], clorgiline, pirlindole [350]	rasagiline (pIC_50_ 7.8) [591], phenelzine (Irreversible inhibition) (p*K*_i_ 7.8) [39], lazabemide (p*K*_i_ 7.1) [200, 532], selegiline (p*K*_i_ 5.7–6) [121, 357], tranylcypromine (pIC_50_ 4.7) [587]	tolcapone (soluble enzyme) (p*K*_i_ 9.6) [317], tolcapone (membrane‐bound enzyme) (p*K*_i_ 9.5) [317], entacapone (soluble enzyme) (p*K*_i_ 9.5) [317], entacapone (membrane‐bound enzyme) (p*K*_i_ 8.7) [317]
Selective inhibitors	–	–	safinamide (p*K*_i_ 6.3) [38]	–
Comments	–	–	–	COMT appears to exist in both membrane‐bound and soluble forms. COMT has also been described to methylate steroids, particularly hydroxyestradiols

### Further reading on Catecholamine turnover

Dauvilliers Y et al. (2015) Catechol‐O‐methyltransferase, dopamine, and sleep‐wake regulation. Sleep Med Rev 22: 47‐53 [PMID:25466290]

Deshwal S et al. (2017) Emerging role of monoamine oxidase as a therapeutic target for cardiovascular disease. Curr Opin Pharmacol 33: 64‐69 [PMID:28528298]

Fisar Z. (2016) Drugs related to monoamine oxidase activity. Prog Neuropsychopharmacol Biol Psychiatry 69: 112‐24 [PMID:26944656]

Ramsay RR. (2016). Molecular aspects of monoamine oxidase B. Prog Neuropsychopharmacol Biol Psychiatry 69: 81‐9 [PMID:26891670]

Waloen K et al. (2017). Tyrosine and tryptophan hydroxylases as therapeutic targets in human disease. Expert Opin Ther Targets 21: 167‐180 [PMID:27973928]

## Ceramide turnover

### Overview

Ceramides are a family of sphingophospholipids synthesized in the endoplasmic reticulum, which mediate cell stress responses, including apoptosis, autophagy and senescence, Serine palmitoyltransferase generates 3‐ketosphinganine, which is reduced to sphinganine(dihydrosphingosine). N‐Acylation allows the formation of dihydroceramides, which are subsequently reduced to form ceramides. Once synthesized, ceramides are trafficked from the ER to the Golgi bound to the ceramide transfer protein, CERT (COL4A3BP, Q9Y5P4). Ceramide can be metabolized via multiple routes, ensuring tight regulation of its cellular levels. Addition of phosphocholine generates sphingomyelin while carbohydrate is added to form glucosyl‐ or galactosylceramides. Ceramidase re‐forms sphingosine or sphinganine from ceramide or dihydroceramide. Phosphorylation of ceramide generates ceramide phosphate. The determination of accurate kinetic parameters for many of the enzymes in the sphingolipid metabolic pathway is complicated by the lipophilic nature of the substrates.

## Serine palmitoyltransferase

### Overview

The functional enzyme is a heterodimer of SPT1 (LCB1) with either SPT2 (LCB2) or SPT3 (LCB2B); the small subunits of SPT (ssSPTa or ssSPTb) bind to the heterodimer to enhance enzymatic activity. The complexes of SPT1/SPT2/ssSPTa and SPT1/SPT2/ssSPTb were most active with palmitoylCoA as substrate, with the latter complex also showing some activity with stearoylCoA [202]. Complexes involving SPT3 appeared more broad in substrate selectivity, with incorporation of myristoylCoA prominent for SPT1/SPT3/ssSPTa complexes, while SP1/SPT3/ssSPTb complexes had similar activity with C16, C18 and C20 acylCoAs [202].

**Table:** 

Nomenclature	serine palmitoyltransferase long chain base subunit 1	serine palmitoyltransferase long chain base subunit 2	serine palmitoyltransferase long chain base subunit 3	serine palmitoyltransferase small subunit A	serine palmitoyltransferase small subunit B
HGNC, UniProt	SPTLC1, O15269	SPTLC2, O15270	SPTLC3, Q9NUV7	SPTSSA, Q969W0	SPTSSB, Q8NFR3
EC number	2.3.1.50: L‐serine + palmitoyl‐CoA ‐>3‐ketosphinganine + coenzyme A + CO_2_	2.3.1.50: L‐serine + palmitoyl‐CoA ‐>3‐ketosphinganine + coenzyme A + CO_2_	2.3.1.50: L‐serine + palmitoyl‐CoA ‐>3‐ketosphinganine + coenzyme A + CO_2_	–	–
Common abreviation	SPT1	SPT2	SPT3	SPTSSA	SPTSSB
Cofactors	pyridoxal phosphate	pyridoxal phosphate	pyridoxal phosphate	–	–
Selective inhibitors	myriocin (p*K*_i_ 9.6) [358] – Mouse	myriocin [358]	myriocin [358]	–	–

## Ceramide synthase

### Overview

This family of enzymes, also known as sphingosine N‐acyltransferase, is located in the ER facing the cytosol with an as‐yet undefined topology and stoichiometry. Ceramide synthase in vitro is sensitive to inhibition by the fungal derived toxin, fumonisin B1.

**Table:** 

Nomenclature	ceramide synthase 1	ceramide synthase 2	ceramide synthase 3	ceramide synthase 4	ceramide synthase 5	ceramide synthase 6
HGNC, UniProt	CERS1, P27544	CERS2, Q96G23	CERS3, Q8IU89	CERS4, Q9HA82	CERS5, Q8N5B7	CERS6, Q6ZMG9
EC number	2.3.1.24: acylCoA + sphinganine ‐> dihydroceramide + coenzyme A sphingosine + acylCoA ‐> ceramide + coenzyme A					
Common abreviation	CERS1	CERS2	CERS3	CERS4	CERS5	CERS6
Substrates	C18‐CoA [543]	C24‐ and C26‐CoA [292]	C26‐CoA and longer [361, 424]	C18‐, C20‐ and C22‐CoA [438]	C16‐CoA [288, 438]	C14‐ and C16‐CoA [360]

## Sphingolipid Δ^4^‐desaturase

### Overview

DEGS1 and DEGS2 are 4TM proteins.

**Table:** 

Nomenclature	delta 4‐desaturase, sphingolipid 1	delta 4‐desaturase, sphingolipid 2
HGNC, UniProt	DEGS1, O15121	DEGS2, Q6QHC5
EC number	1.14.‐.‐	1.14.‐.‐
Cofactors	NAD	NAD
Inhibitors	RBM2‐1B (pIC_50_ 4.7) [63]	–
Comments	Myristoylation of DEGS1 enhances its activity and targets it to the mitochondria [28].	–

### Comments

DEGS1 activity is inhibited by a number of natural products, including curcumin and Δ^9^‐tetrahydrocannabinol[138].

## Sphingomyelin synthase

### Overview

Following translocation from the ER to the Golgi under the influence of the ceramide transfer protein, sphingomyelin synthases allow the formation of sphingomyelin by the transfer of phosphocholine from the phospholipid phosphatidylcholine.

Sphingomyelin synthase‐related protein 1 is structurally related but lacks sphingomyelin synthase activity.

**Table:** 

Nomenclature	sphingomyelin synthase 1	sphingomyelin synthase 2	sterile alpha motif domain containing 8
HGNC, UniProt	SGMS1, Q86VZ5	SGMS2, Q8NHU3	SAMD8, Q96LT4
EC number	2.7.8.27: ceramide + phosphatidylcholine ‐> sphingomyelin + diacylglycerol	2.7.8.27: ceramide + phosphatidylcholine ‐> sphingomyelin + diacylglycerol	2.7.8.‐: ceramide + phosphatidylethanolamine ‐> ceramide phosphoethanolamine
Inhibitors	compound 1j (pIC_50_ 5.7) [301]	compound D24 (pIC_50_ 4.9) [116]	–
Comments	–	Palmitoylation of sphingomyelin synthase 2 may allow targeting to the plasma membrane [517].	–

## Sphingomyelin phosphodiesterase

### Overview

Also known as sphingomyelinase.

**Table:** 

Nomenclature	sphingomyelin phosphodiesterase 1	sphingomyelin phosphodiesterase 2	sphingomyelin phosphodiesterase 3	sphingomyelin phosphodiesterase 4	sphingomyelin phosphodiesterase acid‐like 3A	sphingomyelin phosphodiesterase acid‐like 3B
HGNC, UniProt	SMPD1, P17405	SMPD2, O60906	SMPD3, Q9NY59	SMPD4, Q9NXE4	SMPDL3A, Q92484	SMPDL3B, Q92485
EC number	3.1.4.12: sphingomyelin ‐> ceramide + phosphocholine				3.1.4.‐: sphingomyelin ‐> ceramide + phosphocholine	
Inhibitors	–	inhibitor A (p*K*_i_ 5.8) [586] – Bovine	–	–	–	–

## Neutral sphingomyelinase coupling factors

### Overview

Protein FAN [3] and polycomb protein EED [410] allow coupling between TNF receptors and neutral sphingomyelinase phosphodiesterases.

**Table:** 

Nomenclature	embryonic ectoderm development	neutral sphingomyelinase activation associated factor
HGNC, UniProt	EED, O75530	NSMAF, Q92636
Selective inhibitors	A‐395 (Binding) (p*K*_i_ 9.4) [217]	–

## Ceramide glucosyltransferase

**Table:** 

Nomenclature	UDP‐glucose ceramide glucosyltransferase
HGNC, UniProt	UGCG, Q16739
EC number	2.4.1.80: UDP‐glucose + ceramide = uridine diphosphate + glucosylceramide
Inhibitors	miglustat (p*K*_i_ 5.1) [63]
Comments	Glycoceramides are an extended family of sphingolipids, differing in the content and organization of the sugar moieties, as well as the acyl sidechains.

## Acid ceramidase

### Overview

The six human ceramidases may be divided on the basis of pH optimae into acid, neutral and alkaline ceramidases, which also differ in their subcellular location.

**Table:** 

Nomenclature	N‐acylsphingosine amidohydrolase 1
HGNC, UniProt	ASAH1, Q13510
EC number	3.5.1.23: ceramide ‐>sphingosine + a fatty acid
Comments	This lysosomal enzyme is proteolysed to form the mature protein made up of two chains from the same gene product [274].

## Neutral ceramidases

### Overview

The six human ceramidases may be divided on the basis of pH optimae into acid, neutral and alkaline ceramidases, which also differ in their subcellular location.

**Table:** 

Nomenclature	N‐acylsphingosine amidohydrolase 2	N‐acylsphingosine amidohydrolase 2B
HGNC, UniProt	ASAH2, Q9NR71	ASAH2B, P0C7U1
EC number	3.5.1.23: ceramide ‐>sphingosine + a fatty acid	–
Comments	The enzyme is associated with the plasma membrane [516].	–

### Comments

ASAH2B appears to be an enzymatically inactive protein, which may result from gene duplication and truncation.

## Alkaline ceramidases

### Overview

The six human ceramidases may be divided on the basis of pH optimae into acid, neutral and alkaline ceramidases, which also differ in their subcellular location.

**Table:** 

Nomenclature	alkaline ceramidase 1	alkaline ceramidase 2	alkaline ceramidase 3
HGNC, UniProt	ACER1, Q8TDN7	ACER2, Q5QJU3	ACER3, Q9NUN7
EC number	3.5.1.23: ceramide ‐>sphingosine + a fatty acid	3.5.1.23: ceramide ‐>sphingosine + a fatty acid	3.5.1.‐
Comments	ACER1 is associated with the ER [505].	ACER2 is associated with the Golgi apparatus [582].	ACER3 is associated with the ER and Golgi apparatus [336].

## Ceramide kinase

**Table:** 

Nomenclature	ceramide kinase
HGNC, UniProt	CERK, Q8TCT0
EC number	2.7.1.138: ceramide + ATP ‐> ceramide 1‐phosphate + ADP
Inhibitors	NVP 231 (pIC_50_ 7.9) [188]

### Comments

A ceramide kinase‐like protein has been identified in the human genome (CERKL, Q49MI3).

### Further reading on Ceramide turnover

Aburasayn H et al. (2016) Targeting ceramide metabolism in obesity. Am J Physiol Endocrinol Metab 311: E423‐35 [PMID:27382035]

Adada M et al. (2016) Inhibitors of the sphingomyelin cycle: Sphingomyelin synthases and sphingomyelinases. Chem Phys Lipids 197: 45‐59 [PMID:26200918]

Casals N et al. (2016) Carnitine palmitoyltransferase 1C: From cognition to cancer. Prog Lipid Res 61: 134‐48 [PMID:26708865]

Casasampere M et al. (2016) Inhibitors of dihydroceramide desaturase 1: Therapeutic agents and pharmacological tools to decipher the role of dihydroceramides in cell biology. Chem Phys Lipids 197: 33‐44 [PMID:26248324]

Fucho R et al. (2017) Ceramides and mitochondrial fatty acid oxidation in obesity. FASEB J 31: 1263‐1272 [PMID:28003342]

Hernandez‐Corbacho MJ et al. (2017) Sphingolipids in mitochondria. Biochim Biophys Acta 1862: 56‐68 [PMID:27697478]

Ilan Y. (2016) Compounds of the sphingomyelin‐ceramide‐glycosphingolipid pathways as secondary messenger molecules: new targets for novel therapies for fatty liver disease and insulin resistance. Am J Physiol Gastrointest Liver Physiol 310: G1102‐17 [PMID:27173510]

Iqbal J et al. (2017) Sphingolipids and Lipoproteins in Health and Metabolic Disorders. Trends Endocrinol Metab 28: 506‐518 [PMID:28462811]

Kihara A. (2016) Synthesis and degradation pathways, functions, and pathology of ceramides and epidermal acylceramides. Prog Lipid Res 63: 50‐69 [PMID:27107674]

Petrache I et al. (2016) Ceramide Signaling and Metabolism in Pathophysiological States of the Lung. Annu Rev Physiol 78: 463‐80 [PMID:26667073]

Rodriguez‐Cuenca S et al. (2017) Sphingolipids and glycerophospholipids ‐ The “ying and yang” of lipotoxicity in metabolic diseases. Prog Lipid Res 66: 14‐29 [PMID:28104532]

Sasset L et al. (2016) Sphingolipid De Novo Biosynthesis: A Rheostat of Cardiovascular Homeostasis. Trends Endocrinol Metab 27: 807‐819 [PMID:27562337]

Vogt D et al. (2017) Therapeutic Strategies and Pharmacological Tools Influencing S1P Signaling and Metabolism. Med Res Rev 37: 3‐51 [PMID:27480072]

Wegner MS et al. (2016) The enigma of ceramide synthase regulation in mammalian cells. Prog Lipid Res 63: 93‐119 [PMID:27180613]

## Chromatin modifying enzymes

### Overview

Chromatin modifying enzymes, and other chromatin‐modifying proteins, fall into three broad categories: writers, readers and erasers. The function of these proteins is to dynamically maintain cell identity and regulate processes such as differentiation, development, proliferation and genome integrity via recognition of specific 'marks' (covalent post‐translational modifications) on histone proteins and DNA [280]. In normal cells, tissues and organs, precise co‐ordination of these proteins ensures expression of only those genes required to specify phenotype or which are required at specific times, for specific functions. Chromatin modifications allow DNA modifications not coded by the DNA sequence to be passed on through the genome and underlies heritable phenomena such as X chromosome inactivation, aging, heterochromatin formation, reprogramming, and gene silencing (epigenetic control).

To date at least eight distinct types of modifications are found on histones. These include small covalent modifications such as acetylation, methylation, and phosphorylation, the attachment of larger modifiers such as ubiquitination or sumoylation, and ADP ribosylation, proline isomerization and deimination. Chromatin modifications and the functions they regulate in cells are reviewed by Kouzarides (2007) [280].

Writer proteins include the histone methyltransferases, histone acetyltransferases, some kinases and ubiquitin ligases.

Readers include proteins which contain methyl‐lysine‐recognition motifs such as bromodomains, chromodomains, tudor domains, PHD zinc fingers, PWWP domains and MBT domains.

Erasers include the histone demethylases and histone deacetylases (HDACs and sirtuins).

Dysregulated epigenetic control can be associated with human diseases such as cancer [137], where a wide variety of cellular and protein abberations are known to perturb chromatin structure, gene transcription and ultimately cellular pathways [27, 477]. Due to the reversible nature of epigenetic modifications, chromatin regulators are very tractable targets for drug discovery and the development of novel therapeutics. Indeed, small molecule inhibitors of writers (e.g. azacitidine and decitabine target the DNA methyltransferases DNMT1 and DNMT3 for the treatment of myelodysplastic syndromes [175, 565]) and erasers (e.g. the HDAC inhibitors vorinostat, romidepsin and belinostat for the treatment of T‐cell lymphomas [153, 265]) are already being used in the clinic. The search for the next generation of compounds with improved specificity against chromatin‐associated proteins is an area of intense basic and clinical research [61]. Current progress in this field is reviewed by Simó‐Riudalbas and Esteller (2015) [478].

## 2.1.1.‐ Protein arginine N‐methyltransferases

### Overview

Protein arginine N‐methyltransferases (PRMT, EC 2.1.1.‐) encompass histone arginine N‐methyltransferases (PRMT4, PRMT7, EC 2.1.1.125) and myelin basic protein N‐methyltransferases (PRMT7, EC 2.1.1.126). They are dimeric or tetrameric enzymes which use S‐adenosyl methionine as a methyl donor, generating S‐adenosylhomocysteine as a by‐product. They generate both mono‐methylated and di‐methylated products; these may be symmetric (SDMA) or asymmetric (N^G^,N^G^‐dimethyl‐L‐arginine) versions, where both guanidine nitrogens are monomethylated or one of the two is dimethylated, respectively.

Information on members of this family may be found in the online database.

## 3.5.1.‐ Histone deacetylases (HDACs)

### Overview

Histone deacetylases act as erasers of epigenetic acetylation marks on lysine residues in histones. Removal of the acetyl groups facilitates tighter packing of chromatin (heterochromatin formation) leading to transcriptional repression.

The histone deacetylase family has been classified in to five subfamilies based on phylogenetic comparison with yeast homologues:

Class I contains HDACs 1, 2, 3 and 8

Class IIa contains HDACs 4, 5, 7 and 9

Class IIb contains HDACs 6 and 10

Class III contains the sirtuins (SIRT1‐7)

Class IV contains only HDAC11.

Classes I, II and IV use Zn^+^ as a co‐factor, whereas catalysis by Class III enzymes requires NAD^+^ as a co‐factor, and members of this subfamily have ADP‐ribosylase activity in addition to protein deacetylase function [456].

HDACs have more general protein deacetylase activity, being able to deacetylate lysine residues in non‐histone proteins [90] such as microtubules [233], the hsp90 chaperone [281] and the tumour suppressor p53 [322].

Dysregulated HDAC activity has been identified in cancer cells and tumour tissues [305, 444], making HDACs attractive molecular targets in the search for novel mechanisms to treat cancer [567]. Several small molecule HDAC inhibitors are already approved for clinical use: romidepsin, belinostat, vorinostat, panobinostat, belinostat, valproic acid and tucidinostat. HDACs and HDAC inhibitors currently in development as potential anti‐cancer therapeutics are reviewed by Simó‐Riudalbas and Esteller (2015) [478].

Information on members of this family may be found in the online database.

## Cyclic nucleotide turnover/signalling

### Overview

Cyclic nucleotides are second messengers generated by cyclase enzymes from precursor triphosphates and hydrolysed by phosphodiesterases. The cellular actions of these cyclic nucleotides are mediated through activation of protein kinases (cAMP‐ and cGMP‐dependent protein kinases), ion channels (cyclic nucleotide‐gated, CNG, and hyperpolarization and cyclic nucleotide‐gated, HCN) and guanine nucleotide exchange factors (GEFs, Epac).

## Adenylyl cyclases (ACs)

### Overview

Adenylyl cyclase, E.C. 4.6.1.1, converts ATP to cyclic AMP and pyrophosphate. Mammalian membrane‐bound adenylyl cyclases are typically made up of two clusters of six TM domains separating two intracellular, overlapping catalytic domains that are the target for the nonselective activators forskolin, NKH477(except AC9, [419]) and G*α*_s_(the stimulatory G protein *α* subunit). Adenosine and its derivatives (e.g. 2',5'‐dideoxyadenosine), acting through the P‐site, appear to be physiological inhibitors of adenylyl cyclase activity [527]. Three families of adenylyl cyclase are distinguishable: calmodulin(CALM1 CALM2 CALM3, P62158)‐stimulated (AC1, AC3 and AC8), Ca^2+^‐inhibitable (AC5, AC6 and AC9) and Ca^2+^‐insensitive (AC2, AC4 and AC7) forms.

**Table:** 

Nomenclature	adenylyl cyclase 1	adenylyl cyclase 2 (brain)	adenylyl cyclase 3	adenylyl cyclase 4
HGNC, UniProt	ADCY1, Q08828	ADCY2, Q08462	ADCY3, O60266	ADCY4, Q8NFM4
Common abreviation	AC1	AC2	AC3	AC4
Endogenous activators	calmodulin (CALM1 CALM2 CALM3, P62158), PKC‐evoked phosphorylation [246, 515]	G*β* *γ*, PKC‐evoked phosphorylation [80, 326, 520]	calmodulin (CALM1 CALM2 CALM3, P62158), PKC‐evoked phosphorylation [89, 246]	G*β* *γ* [173]
Endogenous inhibitors	G*α*_i_, G*α*_o_, G*β* *γ* [520, 521]	–	G*α*_i_, RGS2, CaM kinase II‐evoked phosphorylation [479, 521, 562]	PKC‐evoked phophorylation [603]

**Table:** 

Nomenclature	adenylyl cyclase 5	adenylyl cyclase 6	adenylyl cyclase 7	adenylyl cyclase 8	adenylyl cyclase 9
HGNC, UniProt	ADCY5, O95622	ADCY6, O43306	ADCY7, P51828	ADCY8, P40145	ADCY9, O60503
Common abreviation	AC5	AC6	AC7	AC8	AC9
Endogenous activators	PKC‐evoked phophorylation [262]	–	PKC‐evoked phosphorylation [561]	Ca^2+^ [62]	–
Endogenous inhibitors	G*α*_i_, Ca^2+^, PKA‐evoked phosphorylation [240, 243, 521]	G*α*_i_, Ca^2+^, PKA‐evoked phosphorylation, PKC‐evoked phosphorylation [83, 289, 521, 590]	–	–	Ca^2+^/calcineurin [402]
Inhibitors	NKY80 (pIC_50_ 5.2) [52, 390]	NKY80 (pIC_50_ 4.8) [52]	–	–	–

### Comments

Nitric oxide has been proposed to inhibit AC5 and AC6 selectively [223], although it is unclear whether this phenomenon is of physiological significance. A soluble adenylyl cyclase has been described (ADCY10, Q96PN6 [54]), unaffected by either G*α* or G*β* *γ* subunits, which has been suggested to be a cytoplasmic bicarbonate (pH‐insensitive) sensor [82]. It can be inhibited selectively by KH7 (pIC_50_ 5.0‐5.5) [221].

### Further reading on Adenylyl cyclases

Dessauer CW et al. (2017) International Union of Basic and Clinical Pharmacology. CI. Structures and Small Molecule Modulators of Mammalian Adenylyl Cyclases. Pharmacol Rev 69: 93‐139 [PMID:28255005]

Halls ML et al. (2017) Adenylyl cyclase signalling complexes ‐ Pharmacological challenges and opportunities. Pharmacol Ther 172: 171‐180 [PMID:28132906]

Wu L et al. (2016) Adenylate cyclase 3: a new target for anti‐obesity drug development. Obes Rev 17: 907‐14 [PMID:27256589]

## Exchange protein activated by cyclic AMP (EPACs)

### Overview

Epacs are members of a family of guanine nucleotide exchange factors (ENSFM00250000000899), which also includes RapGEF5(GFR, KIAA0277, MR‐GEF, Q92565) and RapGEFL1 (Link‐GEFII, Q9UHV5). They are activated endogenously by cyclic AMP and with some pharmacological selectivity by 8‐pCPT‐2'‐O‐Me‐cAMP[134]. Once activated, Epacs induce an enhanced activity of the monomeric G proteins, Rap1 and Rap2 by facilitating binding of guanosine‐5'‐triphosphate in place of guanosine 5'‐diphosphate, leading to activation of phospholipase C [459].

**Table:** 

Nomenclature	Rap guanine nucleotide exchange factor 3	Rap guanine nucleotide exchange factor 4
HGNC, UniProt	RAPGEF3, O95398	RAPGEF4, Q8WZA2
Common abreviation	Epac1	Epac2
Inhibitors	ESI‐09 (pIC_50_ 5.5) [12]	HJC 0350 (pIC_50_ 6.5) [78], ESI‐09 (pIC_50_ 4.4–5.2) [12, 79]

### Further reading on Exchange protein activated by cyclic AMP (EPAC)

Fujita T et al. (2017) The role of Epac in the heart. Cell Mol Life Sci 74: 591‐606 [PMID:27549789]

Parnell E et al. (2015) The future of EPAC‐targeted therapies: agonism versus antagonism. Trends Pharmacol Sci 36: 203‐14 [PMID:25744542]

Wang P et al. (2017) Exchange proteins directly activated by cAMP (EPACs): Emerging therapeutic targets. Bioorg Med Chem Lett 27: 1633‐1639 [PMID:28283242]

## Nitric oxide (NO)‐sensitive (soluble) guanylyl cyclase

### Overview

Nitric oxide (NO)‐sensitive (soluble) guanylyl cyclase (GTP diphosphate‐lyase (cyclising)), E.C. 4.6.1.2, is a heterodimer comprising a *β*_1_ subunit and one of two alpha subunits (*α*_1_, *α*_2_) giving rise to two functionally indistinguishable isoforms, GC‐1 (*α*_1_ *β*_1_) and GC‐2 (*α*_2_ *β*_1_) [449, 593]. A haem group is associated with the *β* subunit and is the target for the endogenous ligand NO, and, potentially, carbon monoxide [159]. The enzyme converts guanosine‐5'‐triphosphate to the intracellular second messenger cyclic guanosine‐3',5'‐monophosphate (cyclic GMP).

**Table:** 

Nomenclature	Guanylyl cyclase, *α*_1_*β*_1_	Guanylyl cyclase, *α*_2_*β*_1_
Subunits	Guanylyl cyclase *β*_1_ subunit, Guanylyl cyclase *α*_1_ subunit	Guanylyl cyclase *β*_1_ subunit, Guanylyl cyclase *α*_2_ subunit
Common abreviation	GC‐1	GC‐2
Endogenous ligands	NO, CO	NO, CO
Selective activators	YC‐1 [159, 272, 449], cinaciguat [apo‐GC‐1] [500], riociguat [498, 499]	YC‐1 [272, 449], cinaciguat [apo‐GC‐2] [500], riociguat [500, 499]
Selective inhibitors	NS 2028 (pIC_50_ 8.1) [389] – Bovine, ODQ (pIC_50_ 7.5) [177]	ODQ

**Table:** 

Nomenclature	Guanylyl cyclase*α*_1_subunit	Guanylyl cyclase*α*_2_subunit	Guanylyl cyclase *β*_1_ subunit	Guanylyl cyclase *β*_2_ subunit
HGNC, UniProt	GUCY1A3, Q02108	GUCY1A2, P33402	GUCY1B3, Q02153	GUCY1B2, O75343

### Comments

ODQ also shows activity at other haem‐containing proteins [142], while YC‐1 may also inhibit cGMP‐hydrolysing phosphodiesterases [158, 169].

### Further reading on Nitric oxide (NO)‐sensitive (soluble) guanylyl cyclase

Papapetropoulos A et al. (2015) Extending the translational potential of targeting NO/cGMP‐regulated pathways in the CVS. Br J Pharmacol 172: 1397‐414 [PMID:25302549]

Pechanova O et al. (2015) Cardiac NO signalling in the metabolic syndrome. Br J Pharmacol 172: 1415‐33 [PMID:25297560]

Vanhoutte PM et al. (2016) Thirty Years of Saying NO: Sources, Fate, Actions, and Misfortunes of the Endothelium‐Derived Vasodilator Mediator. Circ Res 119: 375‐96 [PMID:27390338]

Yetik‐Anacak G et al. (2015) Gas what: NO is not the only answer to sexual function. Br J Pharmacol 172: 1434‐54 [PMID:24661203]

## Phosphodiesterases, 3',5'‐cyclic nucleotide (PDEs)

### Overview

3',5'‐Cyclic nucleotide phosphodiesterases (PDEs, 3',5'‐cyclic‐nucleotide 5'‐nucleotidohydrolase), E.C. 3.1.4.17, catalyse the hydrolysis of a 3',5'‐cyclic nucleotide (usually cyclic AMP or cyclic GMP). Isobutylmethylxanthine is a nonselective inhibitor with an IC_50_ value in the millimolar range for all isoforms except PDE 8A, 8B and 9A. A 2',3'‐cyclic nucleotide 3'‐phosphodiesterase (E.C. 3.1.4.37 CNPase) activity is associated with myelin formation in the development of the CNS.

**Table:** 

Nomenclature	phosphodiesterase 1A	phosphodiesterase 1B	phosphodiesterase 1C
HGNC, UniProt	PDE1A, P54750	PDE1B, Q01064	PDE1C, Q14123
Common abreviation	PDE1A	PDE1B	PDE1C
Rank order of affinity	cyclic GMP>cyclic AMP	cyclic GMP>cyclic AMP	cyclic GMP = cyclic AMP
Endogenous activators	calmodulin (CALM1 CALM2 CALM3, P62158)	calmodulin (CALM1 CALM2 CALM3, P62158)	calmodulin (CALM1 CALM2 CALM3, P62158)
Inhibitors	crisaborole (pIC_50_ 5.2) [8]	–	–
Selective inhibitors	SCH51866 (pIC_50_ 7.2) [542], vinpocetine (pIC_50_ 5.1) [319]	SCH51866 (pIC_50_ 7.2) [542]	SCH51866 (pIC_50_ 7.2) [542], vinpocetine (pIC_50_ 4.3) [319]

**Table:** 

Nomenclature	phosphodiesterase 2A	phosphodiesterase 3A	phosphodiesterase 3B
HGNC, UniProt	PDE2A, O00408	PDE3A, Q14432	PDE3B, Q13370
Common abreviation	PDE2A	PDE3A	PDE3B
Rank order of affinity	cyclic AMP≫cyclic GMP	–	–
Endogenous activators	cyclic GMP	–	–
Endogenous inhibitors	–	cyclic GMP	cyclic GMP
Inhibitors	milrinone (pIC_50_<6.5) [504]	cilostazol (pIC_50_ 6.7) [504], inamrinone (pIC_50_ 4.8) [480]	–
Selective inhibitors	BAY607550 (pIC_50_ 8.3–8.8) [47], EHNA (pIC_50_ 5.3) [355]	cilostamide (pIC_50_ 7.5) [504], anagrelide (pIC_50_ 7.1–7.3) [257, 341, 349], milrinone (pIC_50_ 6.3–6.4) [131, 504]	cilostamide (pIC_50_ 7.3) [504], cilostazol (pIC_50_ 6.4) [504], milrinone (pIC_50_ 6) [504], inamrinone (pIC_50_ 4.5) [504]
Comments	EHNA is also an inhibitor of adenosine deaminase (E.C. 3.5.4.4).	–	–

**Table:** 

Nomenclature	phosphodiesterase 4A	phosphodiesterase 4B	phosphodiesterase 4C	phosphodiesterase 4D	phosphodiesterase 5A
HGNC, UniProt	PDE4A, P27815	PDE4B, Q07343	PDE4C, Q08493	PDE4D, Q08499	PDE5A, O76074
Common abreviation	PDE4A	PDE4B	PDE4C	PDE4D	PDE5A
Rank order of affinity	cyclic AMP≫cyclic GMP	cyclic AMP≫cyclic GMP	cyclic AMP≫cyclic GMP	cyclic AMP≫cyclic GMP	cyclic GMP>cyclic AMP
Endogenous activators	–	–	–	PKA‐mediated phosphorylation [229]	Protein kinase A, protein kinase G [100]
Inhibitors	ibudilast (pIC_50_ 7.3) [275], RS‐25344 (pIC_50_ 7.2) [453]	roflumilast (pIC_50_ 9.4) [321], ibudilast (pIC_50_ 7.2) [275], RS‐25344 (pIC_50_ 6.5) [453]	RS‐25344 (pIC_50_ 8.1) [453], ibudilast (pIC_50_ 6.6) [275]	RS‐25344 (pIC_50_ 8.4) [453]	gisadenafil (pIC_50_ 8.9) [433], milrinone (pIC_50_ 7.3)
Sub/family‐selective inhibitors	rolipram (pIC_50_ 9) [553], CDP840 (p*K*_i_ 8) [406], Ro20‐1724 (pIC_50_ 6.5) [553]	rolipram (pIC_50_ 9) [553], Ro20‐1724 (pIC_50_ 6.4) [553]	CDP840 (p*K*_i_ 7.7) [406], rolipram (pIC_50_ 6.5) [553], Ro20‐1724 (pIC_50_ 5.4) [553]	CDP840 (p*K*_i_ 8.1) [406], rolipram (pIC_50_ 7.2) [553], Ro20‐1724 (pIC_50_ 6.2) [553]	–
Selective inhibitors	YM976 (pIC_50_ 8.3) [14], apremilast (pIC_50_ 7.8) [457]	–	apremilast (pIC_50_ 6.9) [457]	apremilast (pIC_50_ 7.5) [457]	vardenafil (pIC_50_ 9.7) [51], T0156 (pIC_50_ 9.5) [362], sildenafil (pIC_50_ 8.4–9) [538, 551], tadalafil (pIC_50_ 8.5) [379], SCH51866 (pIC_50_ 7.2) [542], zaprinast (pIC_50_ 6.8) [538]

**Table:** 

Nomenclature	phosphodiesterase 6A	phosphodiesterase 6B	phosphodiesterase 6C	phosphodiesterase 6D	phosphodiesterase 6G	phosphodiesterase 6H
HGNC, UniProt	PDE6A, P16499	PDE6B, P35913	PDE6C, P51160	PDE6D, O43924	PDE6G, P18545	PDE6H, Q13956
Common abreviation	PDE6A	PDE6B	PDE6C	PDE6D	PDE6G	PDE6H
Inhibitors	–	–	sildenafil (pIC_50_ 7.4) [551]	–	–	–

**Table:** 

Nomenclature	phosphodiesterase 7A	phosphodiesterase 7B	phosphodiesterase 8A	phosphodiesterase 8B
HGNC, UniProt	PDE7A, Q13946	PDE7B, Q9NP56	PDE8A, O60658	PDE8B, O95263
Common abreviation	PDE7A	PDE7B	PDE8A	PDE8B
Rank order of affinity	cyclic AMP≫cyclic GMP [353]	cyclic AMP≫cyclic GMP [176]	cyclic AMP≫cyclic GMP [146]	cyclic AMP≫cyclic GMP [214]
Inhibitors	crisaborole (pIC_50_ 6.1) [8]	BRL50481 (pIC_50_ 4.9) [9]	–	–
Selective inhibitors	BRL50481 (pIC_50_ 6.7–6.8) [9, 486]	dipyridamole (pIC_50_ 5.7–6) [179, 455], SCH51866 (pIC_50_ 5.8) [455]	dipyridamole (pIC_50_ 5.1) [146]	dipyridamole (pIC_50_ 4.3) [214]
Comments	PDE7A appears to be membrane‐bound or soluble for PDE7A1 and 7A2 splice variants, respectively	–	–	–

**Table:** 

Nomenclature	phosphodiesterase 9A	phosphodiesterase 10A	phosphodiesterase 11A
HGNC, UniProt	PDE9A, O76083	PDE10A, Q9Y233	PDE11A, Q9HCR9
Common abreviation	PDE9A	PDE10A	PDE11A
Rank order of affinity	cyclic GMP≫cyclic AMP [145]	cyclic AMP, cyclic GMP [161]	cyclic AMP, cyclic GMP [141]
Inhibitors	SCH51866 (pIC_50_ 5.8) [145], zaprinast (pIC_50_ 4.5) [145]	–	tadalafil (pIC_50_ 6.5) [379], BC11‐38 (pIC_50_ 6.5) [79]

### Comments

PDE1A, 1B and 1C appear to act as soluble homodimers, while PDE2A is a membrane‐bound homodimer. PDE3A and PDE3B are membrane‐bound.

PDE4 isoforms are essentially cyclic AMP specific. The potency of YM976 at other members of the PDE4 family has not been reported. PDE4B–D long forms are inhibited by extracellular signal‐regulated kinase (ERK)‐mediated phosphorylation [224, 225]. PDE4A–D splice variants can be membrane‐bound or cytosolic [229]. PDE4 isoforms may be labelled with [^3^H]rolipram.

PDE6 is a membrane‐bound tetramer composed of two catalytic chains (PDE6A or PDE6C and PDE6B), an inhibitory chain (PDE6G or PDE6H) and the PDE6D chain. The enzyme is essentially cyclic GMP specific and is activated by the *α*‐subunit of transducin (G*α*_t_) and inhibited by sildenafil, zaprinast and dipyridamole with potencies lower than those observed for PDE5A. Defects in PDE6B are a cause of retinitis pigmentosa and congenital stationary night blindness.

### Further reading on Phosphodiesterases, 3',5'‐cyclic nucleotide (PDEs)

Das A et al. (2015) PDE5 inhibitors as therapeutics for heart disease, diabetes and cancer. Pharmacol Ther 147: 12‐21 [PMID:25444755]

Jorgensen C et al. (2015) Phosphodiesterase4D (PDE4D)–A risk factor for atrial fibrillation and stroke? J Neurol Sci 359: 266‐74 [PMID:26671126]

Klussmann E. (2016) Protein‐protein interactions of PDE4 family members ‐ Functions, interactions and therapeutic value. Cell Signal 28: 713‐8 [PMID:26498857]

Korkmaz‐Icoz S et al. (2017) Targeting phosphodiesterase 5 as a therapeutic option against myocardial ischaemia/reperfusion injury and for treating heart failure. Br J Pharmacol [PMID:28213937]

Leal LF et al. (2015) Phosphodiesterase 8B and cyclic AMP signaling in the adrenal cortex. Endocrine 50: 27‐31 [PMID:25971952]

Movsesian M. (2016) Novel approaches to targeting PDE3 in cardiovascular disease. Pharmacol Ther 163: 74‐81 [PMID:27108947]

Ricciarelli R et al. (2015) Phosphodiesterase 4D: an enzyme to remember. Br J Pharmacol 172: 4785‐9 [PMID:26211680]

Wu C et al. (2016) Phosphodiesterase‐4 inhibition as a therapeutic strategy for metabolic disorders. Obes Rev 17: 429‐41 [PMID:26997580]

## Cytochrome P450

### Overview

The cytochrome P450 enzyme family (CYP450), E.C. 1.14.‐.‐, were originally defined by their strong absorbance at 450 nm due to the reduced carbon monoxide‐complexed haem component of the cytochromes. They are an extensive family of haem‐containing monooxygenases with a huge range of both endogenous and exogenous substrates. Listed below are the human enzymes; their relationship with rodent CYP450 enzyme activities is obscure in that the species orthologue may not mediate metabolism of the same substrates. Although the majority of CYP450 enzyme activities are concentrated in the liver, the extrahepatic enzyme activities also contribute to patho/physiological processes. Genetic variation of CYP450 isoforms is widespread and likely underlies a significant proportion of the individual variation to drug administration.

## CYP1 family

**Table:** 

Nomenclature	CYP1A1	CYP1A2	CYP1B1
HGNC, UniProt	CYP1A1, P04798	CYP1A2, P05177	CYP1B1, Q16678
EC number	1.14.1.1	1.14.1.1	1.14.1.1
Comments	–	–	Mutations have been associated with primary congenitial glucoma [503]

## CYP2 family

**Table:** 

Nomenclature	CYP2A6	CYP2A7	CYP2C8	CYP2J2	CYP2R1
HGNC, UniProt	CYP2A6, P11509	CYP2A7, P20853	CYP2C8, P10632	CYP2J2, P51589	CYP2R1, Q6VVX0
EC number	1.14.14.1	1.14.14.1	1.14.14.1	1.14.14.1	1.14.13.15
Inhibitors	–	–	phenelzine (p*K*_i_ 5.1) [150]	terfenadine (pIC_50_ 5.1) [287]	–
Comments	Metabolises nicotine.	CYP2A7 does not incorporate haem and is functionally inactive [162]	Converts arachidonic acid to 11(R)‐12(S)‐epoxyeicosatrienoic acid or 14(R)‐15(S)‐epoxyeicosatrienoic acid [596].	Converts arachidonic acid to 14(R)‐15(S)‐epoxyeicosatrienoic acid [579].	Converts vitamin D3 to calcifediol [85].

## CYP3 family

**Table:** 

Nomenclature	CYP3A4
HGNC, UniProt	CYP3A4, P08684
EC number	1.14.13.32: Albendazole + NADPH + O_2_ = albendazole S‐oxide + NADP^+^ + H_2_ 1.14.13.157: 1,8‐cineole + NADPH + O_2_ = 2‐exo‐hydroxy‐1,8‐cineole + NADP^+^ + H_2_O 1.14.13.97: Taurochenodeoxycholate + NADPH + O_2_ = taurohyocholate + NADP^+^ + H_2_O Lithocholate + NADPH + O_2_ = hyodeoxycholate + NADP^+^ + H_2_O 1.14.13.67: quinine + NADPH + O_2_ = 3‐hydroxyquinine + NADP^+^ + H_2_O_2_
Substrates	atorvastatin [155], codeine [155], diazepam [155], tamoxifen [155], erlotinib [90]
Products	4‐hydroxy‐tamoxifen quinone methide [469], 4‐hydroxy‐tamoxifen [469]
Inhibitors	ritonavir (p*K*_i_>7) [266]
Comments	Metabolises a vast range of xenobiotics, including antidepressants, benzodiazepines, calcium channel blockers, and chemotherapeutic agents. CYP3A4 catalyses the 25‐hydroxylation of trihydroxycholestane in liver microsomes [166].

## CYP4 family

**Table:** 

Nomenclature	CYP4A11	CYP4F2	CYP4F3	CYP4F8
HGNC, UniProt	CYP4A11, Q02928	CYP4F2, P78329	CYP4F3, Q08477	CYP4F8, P98187
EC number	1.14.15.3	1.14.13.30	1.14.13.30	1.14.14.1
Inhibitors	–	17‐octadecynoic acid (p*K*_i_ 5.9) [470]	–	–
Comments	Converts lauric acid to 12‐hydroxylauric acid.	Responsible for *ω*‐hydroxylation of LTB_4_, LXB_4_ [359], and tocopherols, including vitamin E [491]	Responsible for *ω*‐hydroxylation of LTB_4_, LXB_4_ [359], and polyunsaturated fatty acids [143, 207]	Converts PGH_2_ to 19‐hydroxyPGH_2_ [60] and 8,9‐EET or 11,12‐EET to 18‐hydroxy‐8,9‐EET or 18‐hydroxy‐11,12‐EET [378].

**Table:** 

Nomenclature	CYP4F12	CYP4F22	CYP4V2	CYP4X1	CYP4Z1
HGNC, UniProt	CYP4F12, Q9HCS2	CYP4F22, Q6NT55	CYP4V2, Q6ZWL3	CYP4X1, Q8N118	CYP4Z1, Q86W10
EC number	1.14.14.1	1.14.14.‐	1.14.‐.‐	1.14.14.1	1.14.14.1
Comments	AC004597.1 (ENSG00000225607) is described as being highly similar to CYP4F12	Converts arachidonic acid to 16‐HETE and 18‐HETE [378].	Converts myristic acid to 14‐hydroxymyristic acid [372].	Converts anandamide to 14,15‐epoxyeicosatrienoic ethanolamide [497].	Converts lauric acid to 12‐hydroxylauric acid.

### Comments

Converts lauric acid to 12‐hydroxylauric acid.

## CYP5, CYP7 and CYP8 families

**Table:** 

Nomenclature	CYP5A1	CYP7A1	CYP7B1	CYP8A1	CYP8B1
HGNC, UniProt	TBXAS1, P24557	CYP7A1, P22680	CYP7B1, O75881	PTGIS, Q16647	CYP8B1, Q9UNU6
EC number	5.3.99.5: PGH_2_ = thromboxane A_2_	1.14.13.17	1.14.13.100	5.3.99.4	1.14.13.95
Common name	Thromboxane synthase	–	–	Prostacyclin synthase	–
Comments	Inhibited by dazoxiben [427] and camonagrel [194].	Converts cholesterol to 7*α*‐hydroxycholesterol [379].	Converts dehydroepiandrosterone to 7*α*‐DHEA [445].	Converts PGH_2_ to PGI_2_ [209]. Inhibited by tranylcypromine [193]	Converts 7*α*‐hydroxycholest‐4‐en‐3‐one to 7‐alpha,12*α*‐dihydroxycholest‐4‐en‐3‐one (in rabbit) [239] in the biosynthesis of bile acids.

## CYP11, CYP17, CYP19, CYP20 and CYP21 families

**Table:** 

Nomenclature	CYP11A1	CYP11B1	CYP11B2
HGNC, UniProt	CYP11A1, P05108	CYP11B1, P15538	CYP11B2, P19099
EC number	1.14.15.6	1.14.15.4	1.14.15.4 1.14.15.5
Common name	–	–	Aldosterone synthase
Inhibitors	mitotane [297, 303]	metyrapone (pIC_50_ 7.8) [602], mitotane	osilodrostat (pIC_50_ 9.7) [585]
Comments	Converts cholesterol to pregnenolone plus 4‐methylpentanal.	Converts deoxycortisone and 11‐deoxycortisol to cortisone and cortisol, respectively. Loss‐of‐function mutations are associated with familial adrenal hyperplasia and hypertension. Inhibited by metyrapone [558]	Converts corticosterone to aldosterone

**Table:** 

Nomenclature	CYP17A1	CYP19A1	CYP20A1	CYP21A2
HGNC, UniProt	CYP17A1, P05093	CYP19A1, P11511	CYP20A1, Q6UW02	CYP21A2, P08686
EC number	1.14.99.9	1.14.14.1	1.14.‐.‐	1.14.99.10
Common name	–	Aromatase	–	–
Inhibitors	abiraterone (pIC_50_ 7.1–8.4) [413, 417]	anastrozole (pIC_50_ 7.8) [367], aminoglutethimide [405]	–	(2S,4S)‐ketoconazole (pIC_50_ 5.3) [447] – Rat
Selective inhibitors	galeterone (pIC_50_ 6.5) [204]	letrozole (p*K*_i_ 10.7) [346], exemestane (pIC_50_ 7.3) [92], testolactone (p*K*_i_ 4.5) [102]	–	–
Comments	Converts pregnenolone and progesterone to 17*α*‐hydroxypregnenolone and 17*α*‐hydroxyprogesterone, respectively. Converts 17*α*‐hydroxypregnenolone and 17*α*‐hydroxyprogesterone to dehydroepiandrosterone and androstenedione, respectively. Converts corticosterone to cortisol.	Converts androstenedione and testosterone to estrone and 17*β*‐estradiol, respectively. Inhibited by anastrozole [415], and letrozole [35]	–	Converts progesterone and 17*α*‐hydroxyprogesterone to deoxycortisone and 11‐deoxycortisol, respectively

## CYP24, CYP26 and CYP27 families

**Table:** 

Nomenclature	CYP24A1	CYP26A1	CYP26B1	CYP27A1	CYP27B1
HGNC, UniProt	CYP24A1, Q07973	CYP26A1, O43174	CYP26B1, Q9NR63	CYP27A1, Q02318	CYP27B1, O15528
EC number	1.14.13.126	1.14.‐.‐	1.14.‐.‐	1.14.13.15	1.14.13.13
Common name	–	–	–	Sterol 27‐hydroxylase	–
Comments	Converts 1,25‐dihydroxyvitamin D3 (calcitriol) to 1*α*,24R,25‐trihydroxyvitamin D_3_.	Converts retinoic acid to 4‐hydroxyretinoic acid. Inhibited by liarozole	Converts retinoic acid to 4‐hydroxyretinoic acid.	Converts cholesterol to 27‐hydroxycholesterol.	Converts 25‐hydroxyvitamin D_3_ to 1,25‐dihydroxyvitamin D3 (calcitriol)

## CYP39, CYP46 and CYP51 families

**Table:** 

Nomenclature	CYP39A1	CYP46A1	CYP51A1
HGNC, UniProt	CYP39A1, Q9NYL5	CYP46A1, Q9Y6A2	CYP51A1, Q16850
EC number	1.14.13.99	1.14.13.98	–
Common name	–	Cholesterol 24‐hydroxylase	Lanosterol 14‐*α*‐demethylase
Inhibitors	–	–	azalanstat (p*K*_i_ 9.1) [549]
Comments	Converts 24‐hydroxycholesterol to 7*α*,24‐dihydroxycholesterol [302].	Converts cholesterol to 24(S)‐hydroxycholesterol.	Converts lanosterol to 4,4‐dimethylcholesta‐8.14.24‐trienol.

### Further reading on Cytochrome P450

Backman JT et al. (2016) Role of Cytochrome P450 2C8 in Drug Metabolism and Interactions. Pharmacol Rev 68: 168‐241 [PMID:26721703]

Davis CM et al. (2017) Cytochrome P450 eicosanoids in cerebrovascular function and disease. Pharmacol Ther [PMID:28527918]

Ghosh D et al. (2016) Recent Progress in the Discovery of Next Generation Inhibitors of Aromatase from the Structure‐Function Perspective. J Med Chem 59: 5131‐48 [PMID:26689671]

Go RE et al. (2015) Cytochrome P450 1 family and cancers. J Steroid Biochem Mol Biol 147: 24‐30 [PMID:25448748]

Guengerich FP et al. (2016) Recent Structural Insights into Cytochrome P450 Function. Trends Pharmacol Sci 37: 625‐40 [PMID:27267697]

Isvoran A et al. (2017) Pharmacogenomics of the cytochrome P450 2C family: impacts of amino acid variations on drug metabolism. Drug Discov Today 22: 366‐376 [PMID:27693711]

Jamieson KL et al. (2017) Cytochrome P450‐derived eicosanoids and heart function. Pharmacol Ther [PMID:28551025]

Mak PJ et al. (2017) Spectroscopic studies of the cytochrome P450 reaction mechanisms. Biochim Biophys Acta [PMID:28668640]

Moutinho M et al. (2016) Cholesterol 24‐hydroxylase: Brain cholesterol metabolism and beyond. Biochim Biophys Acta 1861 1911‐1920 [PMID:27663182]

Shalan H et al. (2017) Keeping the spotlight on cytochrome P450. Biochim Biophys Acta [PMID:28599858]

## Endocannabinoid turnover

### Overview

The principle endocannabinoids are 2‐acylglycerol esters, such as 2‐arachidonoylglycerol (2AG), and N‐acylethanolamines, such as anandamide(N‐arachidonoylethanolamine, AEA). The glycerol esters and ethanolamides are synthesised and hydrolysed by parallel, independent pathways. Mechanisms for release and re‐uptake of endocannabinoids (and related entities) are unclear, although candidates for intracellular transport have been suggested. For the generation of 2‐arachidonoylglycerol, the key enzyme involved is diacylglycerol lipase (DGL), whilst several routes for anandamide synthesis have been described, the best characterized of which involves N‐acylphosphatidylethanolamine‐phospholipase D (NAPE‐PLD, [476]). A transacylation enzyme which forms N‐acylphosphatidylethanolamines has recently been identified as a cytosolic enzyme, PLA2G4E(Q3MJ16) [383]. In vitro experiments indicate that the endocannabinoids are also substrates for oxidative metabolism via cyclooxygenase, lipoxygenase and cytochrome P450 enzyme activities [11, 154, 488].

## N‐Acylethanolamine turnover

**Table:** 

Nomenclature	N‐Acylphosphatidylethanolamine‐phospholipase D	Fatty acid amide hydrolase	Fatty acid amide hydrolase‐2	N‐Acylethanolamine acid amidase
HGNC, UniProt	NAPEPLD, Q6IQ20	FAAH, O00519	FAAH2, Q6GMR7	NAAA, Q02083
EC number	–	3.5.1.99: anandamide + H_2_O <=> arachidonic acid + ethanolamine oleamide + H_2_O <=> oleic acid + NH_3_ The enzyme is responsible for the catabolism of neuromodulatory fatty acid amides, including anandamide and oleamide: anandamide + H_2_O <=> arachidonic acid + ethanolamine oleamide + H_2_O <=> oleic acid + NH_3_	3.5.1.99: anandamide + H_2_O <=> arachidonic acid + ethanolamine oleamide + H_2_O <=> oleic acid + NH_3_ The enzyme is responsible for the catabolism of neuromodulatory fatty acid amides, including anandamide and oleamide: anandamide + H_2_O <=> arachidonic acid + ethanolamine oleamide + H_2_O <=> oleic acid + NH_3_	3.5.1.‐
Common abreviation	NAPE‐PLD	FAAH	FAAH2	NAAA
Rank order of affinity	–	anandamide>oleamide>N‐oleoylethanolamide>N‐palmitoylethanolamine [563]	oleamide>N‐oleoylethanolamide>anandamide>N‐palmitoylethanolamine [563]	N‐palmitoylethanolamine>MEA>SEA≥N‐oleoylethanolamide>anandamide [539]
Selective inhibitors	–	JNJ1661010 (pIC_50_ 7.8) [264], PF750 (pIC_50_ 6.3–7.8) [5], OL135 (pIC_50_ 7.4) [563], URB597 (pIC_50_ 6.3–7) [563], PF3845 (pIC_50_ 6.6) [6]	OL135 (pIC_50_ 7.9–8.4) [261, 563], URB597 (pIC_50_ 7.5–8.3) [261, 563]	S‐OOPP (pIC_50_ 6.4) [489] – Rat, CCP (pIC_50_ 5.3) [535]
Comments	NAPE‐PLD activity appears to be enhanced by polyamines in the physiological range [311], but fails to transphosphatidylate with alcohols [408] unlike phosphatidylcholine‐specific phospholipase D.	–	The FAAH2 gene is found in many primate genomes, marsupials, and other distantly related vertebrates, but not a variety of lower placental mammals, including mouse and rat [563].	–

### Comments

Routes for N‐acylethanolamine biosynthesis other than through NAPE‐PLD activity have been identified [536].

## 2‐Acylglycerol ester turnover

**Table:** 

Nomenclature	Diacylglycerol lipase *α*	Diacylglycerol lipase *β*	Monoacylglycerol lipase	*αβ*‐Hydrolase 6
HGNC, UniProt	DAGLA, Q9Y4D2	DAGLB, Q8NCG7	MGLL, Q99685	ABHD6, Q9BV23
EC number	3.1.1.‐	3.1.1.‐	3.1.1.23	3.1.1.23
Common abreviation	DAGL*α*	DAGL*β*	MAGL	ABHD6
Endogenous substrates	diacylglycerol	diacylglycerol	2‐oleoyl glycerol = 2‐arachidonoylglycerol≫anandamide [181]	1‐arachidonoylglycerol >2‐arachidonoylglycerol> 1‐oleoylglycerol >2‐oleoyl glycerol [375]
Selective inhibitors	orlistat (pIC_50_ 7.2) [40], RHC80267 (pIC_50_ 4.2) [255]	orlistat (pIC_50_ 7) [40], RHC80267	JJKK 048 (pIC_50_ 9.3) [1], KML29 (pIC_50_ 8.5) [77], JZL184 (pIC_50_ 8.1) [314]	WWL70 (pIC_50_ 7.2) [299], WWL123 (pIC_50_ 6.4) [21]
Comments	–	–	–	WWL70 has also been suggested to have activity at oxidative metabolic pathways independent of ABHD6 [513].

Comments on Endocannabinoid turnover: Many of the compounds described as inhibitors are irreversible and so potency estimates will vary with incubation time. FAAH2 is not found in rodents [563] and a few of the inhibitors described have been assessed at this enzyme activity. 2‐arachidonoylglycerol has been reported to be hydrolysed by multiple enzyme activities from neural preparations, including ABHD2(P08910) [356], ABHD12(Q8N2K0) [44], neuropathy target esterase (PNPLA6, Q8IY17 [338]) and carboxylesterase 1 (CES1, P23141 [581]). ABHD2(P08910) has also been described as a triacylglycerol lipase and ester hydrolase [329], while ABHD12(Q8N2K0) is also able to hydrolyse lysophosphatidylserine [531]. ABHD12(Q8N2K0) has been described to be inhibited selectively by triterpenoids, such as betulinic acid [401].

### Further reading on Endocannabinoid turnover

Blankman JL et al. (2013) Chemical probes of endocannabinoid metabolism. Pharmacol. Rev. 65: 849‐71 [PMID:23512546]

Janssen FJ et al. (2016) Inhibitors of diacylglycerol lipases in neurodegenerative and metabolic disorders. Bioorg. Med. Chem. Lett. 26: 3831‐7 [PMID:27394666]

Ueda N et al. (2013) Metabolism of endocannabinoids and related N‐acylethanolamines: canonical and alternative pathways. FEBS J. 280: 1874‐94 [PMID:23425575]

Wellner N et al. (2013) N‐acylation of phosphatidylethanolamine and its biological functions in mammals. Biochim. Biophys. Acta 1831: 652‐62 [PMID:23000428]

## Eicosanoid turnover

### Overview

Eicosanoids are 20‐carbon fatty acids, where the usual focus is the polyunsaturated analogue arachidonic acid and its metabolites. Arachidonic acid is thought primarily to derive from phospholipase A2 action on membrane phosphatidylcholine, and may be re‐cycled to form phospholipid through conjugation with coenzyme A and subsequently glycerol derivatives. Oxidative metabolism of arachidonic acid is conducted through three major enzymatic routes: cyclooxygenases; lipoxygenases and cytochrome P450‐like epoxygenases, particularly CYP2J2. Isoprostanes are structural analogues of the prostanoids (hence the nomenclature D‐, E‐, F‐isoprostanes and isothromboxanes), which are produced in the presence of elevated free radicals in a non‐enzymatic manner, leading to suggestions for their use as biomarkers of oxidative stress. Molecular targets for their action have yet to be defined.

## Cyclooxygenase

### Overview

Prostaglandin (PG) G/H synthase, most commonly referred to as cyclooxygenase (COX, (5Z,8Z,11Z,14Z)‐icosa‐5,8,11,14‐tetraenoate,hydrogen‐donor : oxygen oxidoreductase) activity, catalyses the formation of PGG_2_ from arachidonic acid. Hydroperoxidase activity inherent in the enzyme catalyses the formation of PGH_2_ from PGG_2_. COX‐1 and ‐2 can be nonselectively inhibited by ibuprofen, ketoprofen, naproxen, indomethacin and paracetamol (acetaminophen). PGH_2_ may then be metabolised to prostaglandins and thromboxanes by various prostaglandin synthases in an apparently tissue‐dependent manner.

**Table:** 

Nomenclature	COX‐1	COX‐2
HGNC, UniProt	PTGS1, P23219	PTGS2, P35354
EC number	1.14.99.1: Hydrogen donor + arachidonic acid + 2O_2_ = hydrogen acceptor + H_2_O + PGH_2_ arachidonic acid =>PGG_2_ =>PGH_2_ This enzyme is also associated with the following reaction:: docosahexaenoic acid => PGH_3_	1.14.99.1: Hydrogen donor + arachidonic acid + 2O_2_ = hydrogen acceptor + H_2_O + PGH_2_ arachidonic acid =>PGG_2_ =>PGH_2_ This enzyme is also associated with the following reaction:: docosahexaenoic acid => PGH_3_
Selective inhibitors	ketorolac (pIC_50_ 9.7) [557], FR122047 (pIC_50_ 7.5) [382]	celecoxib (pIC_50_ 8.7) [41], valdecoxib (pIC_50_ 8.3) [512], diclofenac (pIC_50_ 7.7) [45], rofecoxib (pIC_50_ 6.1–6.5) [557], lumiracoxib (p*K*_i_ 6.5) [46], meloxicam (pIC_50_ 6.3) [294], etoricoxib (pIC_50_ 6) [439]

## Prostaglandin synthases

### Overview

Subsequent to the formation of PGH_2_, the cytochrome P450 activities thromboxane synthase (CYP5A1, TBXAS1, P24557, EC 5.3.99.5) and prostacyclin synthase (CYP8A1, PTGIS, Q16647, EC 5.3.99.4) generate thromboxane A_2_ and prostacyclin (PGI_2_), respectively (see Cytochrome P450s). Additionally, multiple enzyme activities are able to generate prostaglandin E_2_(PGE_2_), prostaglandin D_2_(PGD_2_) and prostaglandin F_2*α*_(PGF_2*α*_). PGD_2_ can be metabolised to 9*α*,11*β*‐prostacyclin F_2*α*_ through the multifunctional enzyme activity of AKR1C3. PGE_2_ can be metabolised to 9*α*,11*β*‐prostaglandin F_2*α*_ through the 9‐ketoreductase activity of CBR1. Conversion of the 15‐hydroxyecosanoids, including prostaglandins, lipoxins and leukotrienes to their keto derivatives by the NAD‐dependent enzyme HPGD leads to a reduction in their biological activity.

**Table:** 

Nomenclature	mPGES1	mPGES2	cPGES	L‐PGDS
HGNC, UniProt	PTGES, O14684	PTGES2, Q9H7Z7	PTGES3, Q15185	PTGDS, P41222
EC number	5.3.99.3: PGH_2_ = PGE_2_	5.3.99.3: PGH_2_ = PGE_2_	5.3.99.3: PGH_2_ = PGE_2_	5.3.99.2: PGH_2_ = PGD_2_
Cofactors	glutathione	dihydrolipoic acid	–	–
Comments	–	–	Phosphorylated and activated by casein kinase 2 (CK2) [370]. Appears to regulate steroid hormone function by interaction with dimeric hsp90 [74, 253].	–

**Table:** 

Nomenclature	H‐PGDS	AKR1C3	CBR1	HPGD
HGNC, UniProt	HPGDS, O60760	AKR1C3, P42330	CBR1, P16152	HPGD, P15428
EC number	5.3.99.2: PGH_2_ = PGD_2_	1.3.1.20 1.1.1.188: PGD_2_ + NADP^+^ = PGF_2*α*_ + NADPH + H^+^ 1.1.1.64 1.1.1.239 1.1.1.213	1.1.1.184 1.1.1.189: PGE_2_ + NADP^+^ = PGF_2*α*_ + NADPH + H^+^ 1.1.1.197	1.1.1.141 15‐hydroxyprostaglandins => 15‐ketoprostaglandins LXA_4_ => 15‐keto‐lipoxin A_4_
Cofactors	–	NADP^+^	NADP^+^	–
Inhibitors	HQL‐79 (pIC_50_ 5.3–5.5) [16]	tolfenamic acid (p*K*_i_ 8.1) [421] flufenamic acid, indomethacin, flavonoids [344, 484]	wedelolactone (pIC_50_ 5.4) [604]	
Comments	–	Also acts as a hydroxysteroid dehydrogenase activity.	–	–

### Comments

YS121 has been reported to inhibit mPGES1 and 5‐LOX with a pIC_50_value of 5.5 [276].

## Lipoxygenases

### Overview

The lipoxygenases (LOXs) are a structurally related family of non‐heme iron dioxygenases that function in the production, and in some cases metabolism, of fatty acid hydroperoxides. For arachidonic acid as substrate, these products are hydroperoxyeicosatetraenoic acids (HPETEs). In humans there are five lipoxygenases, the 5S‐(arachidonate : oxygen 5‐oxidoreductase), 12R‐(arachidonate 12‐lipoxygenase, 12R‐type), 12S‐(arachidonate : oxygen 12‐oxidoreductase), and two distinct 15S‐(arachidonate : oxygen 15‐oxidoreductase) LOXs that oxygenate arachidonic acid in different positions along the carbon chain and form the corresponding 5S‐, 12S‐, 12R‐, or 15S‐hydroperoxides, respectively.

**Table:** 

Nomenclature	5‐LOX	12R‐LOX	12S‐LOX	15‐LOX‐1	15‐LOX‐2	E‐LOX
HGNC, UniProt	ALOX5, P09917	ALOX12B, O75342	ALOX12, P18054	ALOX15, P16050	ALOX15B, O15296	ALOXE3, Q9BYJ1
EC number	1.13.11.34: arachidonic acid + O_2_ = LTA_4_ + H_2_O	1.13.11.31 arachidonic acid + O_2_ =>12R‐HPETE	1.13.11.31 arachidonic acid + O_2_ =>12S‐HPETE	1.13.11.33: arachidonic acid + O_2_ = 15S‐HPETE linoleic acid + O_2_ => 13S‐HPODE	1.13.11.33: arachidonic acid + O_2_ = 15S‐HPETE	1.13.11.‐
Endogenous substrates	arachidonic acid	–	–	–	–	12R‐HPETE
Endogenous activators	5‐LOX activating protein (ALOX5AP, P20292)	–	–	–	–	–
Endogenous inhibitors	Protein kinase A‐mediated phosphorylation [324]	–	–	–	–	–
Selective inhibitors	CJ13610 (pIC_50_ 7.2) [144], PF‐04191834 (pIC_50_ 6.6) [342], zileuton	–	–	compound 34 (p*K*_i_>8) [425]	–	–
Comments	FLAP activity can be inhibited by MK‐886 [124] and BAY‐X1005 [210] leading to a selective inhibition of 5‐LOX activity	–	–	–	–	E‐LOX metabolises the product from the 12R‐lipoxygenase (12R‐HPETE) to a specific epoxyalcohol compound [592].

### Comments

An 8‐LOX (EC 1.13.11.40, arachidonate:oxygen 8‐oxidoreductase) may be the mouse orthologue of 15‐LOX‐2 [167]. Some general LOX inhibitors are nordihydroguiaretic acid and esculetin. Zileuton and caffeic acid are used as 5‐lipoxygenase inhibitors, while baicalein and CDC are 12‐lipoxygenase inhibitors. The specificity of these inhibitors has not been rigorously assessed with all LOX forms: baicalein, along with other flavonoids, such as fisetin and luteolin, also inhibits 15‐LOX‐1 [450].

## Leukotriene and lipoxin metabolism

### Overview

Leukotriene A_4_(LTA_4_), produced by 5‐LOX activity, and lipoxins may be subject to further oxidative metabolism; *ω*‐hydroxylation is mediated by CYP4F2 and CYP4F3, while *β*‐oxidation in mitochondria and peroxisomes proceeds in a manner dependent on coenzyme A conjugation. Conjugation of LTA_4_ at the 6 position with reduced glutathione to generate LTC_4_ occurs under the influence of leukotriene C_4_ synthase, with the subsequent formation of LTD_4_ and LTE_4_, all three of which are agonists at CysLT receptors. LTD_4_ formation is catalysed by *γ*‐glutamyltransferase, and subsequently dipeptidase 2 removes the terminal glycine from LTD_4_ to generate LTE_4_. Leukotriene A_4_ hydrolase converts the 5,6‐epoxide LTA_4_ to the 5‐hydroxylated LTB_4_, an agonist for BLT receptors. LTA_4_ is also acted upon by 12S‐LOX to produce the trihydroxyeicosatetraenoic acids lipoxins LXA_4_ and LXB_4_. Treatment with a LTA_4_ hydrolase inhibitor in a murine model of allergic airway inflammation increased LXA_4_ levels, in addition to reducing LTB_4_, in lung lavage fluid [429].

LTA_4_ hydrolase is also involved in biosynthesis of resolvin Es. Aspirin has been reported to increase endogenous formation of 18S‐hydroxyeicosapentaenoate (18S‐HEPE) compared with 18R‐HEPE, a resolvin precursor. Both enantiomers may be metabolised by human recombinant 5‐LOX; recombinant LTA_4_hydrolase converted chiral 5S(6)‐epoxide‐containing intermediates to resolvin E1 and 18S‐resolvin E1 [384].

**Table:** 

Nomenclature	Leukotriene C4 synthase	*γ*‐Glutamyltransferase	Dipeptidase 1	Dipeptidase 2
HGNC, UniProt	LTC4S, Q16873	GGCT, O75223	DPEP1, P16444	DPEP2, Q9H4A9
EC number	4.4.1.20: LTC_4_ = glutathione + LTA_4_	2.3.2.2: (5‐L‐glutamyl)‐peptide + an amino acid = a peptide + a 5‐L‐glutamyl amino acid LTC_4_ + H_2_O =>LTD_4_ + L‐glutamate	3.4.13.19: LTD_4_ + H_2_O = LTE_4_ + glycine	3.4.13.19: LTD_4_ + H_2_O = LTE_4_ + glycine
Inhibitors	–	–	cilastatin (p*K*_i_ 6) [189]	–

### Comments

LTA4H is a member of a family of arginyl aminopeptidases (ENSFM00250000001675), which also includes aminopeptidase B (RNPEP, 9H4A4) and aminopeptidase B‐like 1 (RNPEPL1, Q9HAU8). Dipeptidase 1 and 2 are members of a family of membrane dipeptidases, which also includes (DPEP3, Q9H4B8) for which LTD_4_ appears not to be a substrate.

### Further reading on Eicosanoid turnover

Ackermann JA et al. (2017) The double‐edged role of 12/15‐lipoxygenase during inflammation and immunity Biochim Biophys Acta 1862: 371‐381 [PMID:27480217]

Grosser T et al. (2017) The Cardiovascular Pharmacology of Nonsteroidal Anti‐Inflammatory Drugs. Trends Pharmacol Sci [PMID:28651847]

Horn T et al. (2015) Evolutionary aspects of lipoxygenases and genetic diversity of human leukotriene signaling. Prog Lipid Res 57: 13‐39 [PMID:25435097]

Joshi YB et al. (2015) The 12/15‐lipoxygenase as an emerging therapeutic target for Alzheimer's disease. Trends Pharmacol Sci 36: 181‐6 [PMID:25708815]

Koeberle A et al. (2015) Perspective of microsomal prostaglandin E2 synthase‐1 as drug target in inflammation‐related disorders. Biochem Pharmacol 98: 1‐15 [PMID:26123522]

Kuhn H et al. (2015) Mammalian lipoxygenases and their biological relevance. Biochim Biophys Acta 1851: 308‐30 [PMID:25316652]

Patrignani P et al. (2015) Cyclooxygenase inhibitors: From pharmacology to clinical read‐outs. Biochim Biophys Acta 1851: 422‐32 [PMID:25263946]

Radmark O et al. (2015) 5‐Lipoxygenase, a key enzyme for leukotriene biosynthesis in health and disease. Biochim Biophys Acta 1851: 331‐9 [PMID:25152163]

Sasaki Y et al. (2017) Role of prostacyclin synthase in carcinogenesis. Prostaglandins Other Lipid Mediat [PMID:28506876]

Seo MJ et al. (2017) Prostaglandin synthases: Molecular characterization and involvement in prostaglandin biosynthesis. Prog Lipid Res 66: 50‐68 [PMID:28392405]

Vitale P et al. (2016) COX‐1 Inhibitors: Beyond Structure Toward Therapy. Med Res Rev 36: 641‐71 [PMID:27111555]

## GABA turnover

### Overview

The inhibitory neurotransmitter *γ*‐aminobutyrate (GABA, 4‐aminobutyrate) is generated in neurones by glutamic acid decarboxylase. GAD1 and GAD2 are differentially expressed during development, where GAD2 is thought to subserve a trophic role in early life and is distributed throughout the cytoplasm. GAD1 is expressed in later life and is more associated with nerve terminals [136] where GABA is principally accumulated in vesicles through the action of the vesicular inhibitory amino acid transporter SLC32A1. The role of *γ*‐aminobutyraldehyde dehydrogenase (ALDH9A1) in neurotransmitter GABA synthesis is less clear. Following release from neurons, GABA may interact with either GABA_A_ or GABA_B_ receptors and may be accumulated in neurones and glia through the action of members of the SLC6 family of transporters. Successive metabolism through GABA transaminase and succinate semialdehyde dehydrogenase generates succinic acid, which may be further metabolized in the mitochondria in the tricarboxylic acid cycle.

**Table:** 

Nomenclature	Glutamic acid decarboxylase 1	Glutamic acid decarboxylase 2
HGNC, UniProt	GAD1, Q99259	GAD2, Q05329
EC number	4.1.1.15: L‐glutamic acid + H^+^ ‐>GABA + CO_2_	4.1.1.15: L‐glutamic acid + H^+^ ‐>GABA + CO_2_
Common abreviation	GAD1	GAD2
Endogenous substrates	L‐glutamic acid, L‐aspartic acid	L‐glutamic acid, L‐aspartic acid
Products	GABA	GABA
Cofactors	pyridoxal phosphate	pyridoxal phosphate
Selective inhibitors	s‐allylglycine	s‐allylglycine
Comments	L‐aspartic acid is a less rapidly metabolised substrate of mouse brain glutamic acid decarboxylase generating *β*‐alanine [577]. Autoantibodies against GAD1 and GAD2 are elevated in type 1 diabetes mellitus and neurological disorders (see Further reading).	

**Table:** 

Nomenclature	aldehyde dehydrogenase 9 family member A1	4‐aminobutyrate aminotransferase	aldehyde dehydrogenase 5 family member A1
HGNC, UniProt	ALDH9A1, P49189	ABAT, P80404	ALDH5A1, P51649
EC number	1.2.1.19: 4‐aminobutanal + NAD + H_2_O = GABA + NADH + H^+^ 1.2.1.47: 4‐trimethylammoniobutanal + NAD + H_2_O = 4‐trimethylammoniobutanoate + NADPH + 2H^+^ 1.2.1.3: an aldehyde + H_2_O + NAD = a carboxylate + 2H^+^ + NADH	2.6.1.19: GABA + *α*‐ketoglutaric acid = L‐glutamic acid + 4‐oxobutanoate 2.6.1.22: (S)‐3‐amino‐2‐methylpropanoate + *α*‐ketoglutaric acid = 2‐methyl‐3‐oxopropanoate + L‐glutamic acid	1.2.1.24: 4‐oxobutanoate + NAD + H_2_O = succinic acid + NADH + 2H^+^ 4‐hydroxy‐trans‐2‐nonenal + NAD + H_2_O = 4‐hydroxy‐trans‐2‐nonenoate + NADH + 2H^+^
Common abreviation	–	GABA‐T	SSADH
Cofactors	NAD	pyridoxal phosphate	NAD [469]
Inhibitors	–	vigabatrin (Irreversible inhibition) (p*K*_i_ 3.1) [306, 475]	4‐acryloylphenol (pIC_50_ 6.5) [519]

### Further reading on GABA turnover

Koenig MK et al. (2017) Phenotype of GABA‐transaminase deficiency. Neurology 88: 1919‐1924 [PMID:28411234]

Lee H et al. (2015) Ornithine aminotransferase versus GABA aminotransferase: implications for the design of new anticancer drugs. Med Res Rev 35: 286‐305 [PMID:25145640]

McQuail JA et al. (2015) Molecular aspects of age‐related cognitive decline: the role of GABA signaling. Trends Mol Med 21: 450‐60 [PMID:26070271]

## Glycerophospholipid turnover

### Overview

Phospholipids are the basic barrier components of membranes in eukaryotic cells divided into glycerophospholipids (phosphatidic acid, phosphatidylethanolamine, phosphatidylcholine, phosphatidylserine, phosphatidylinositol and its phosphorylated derivatives) and sphingolipids (ceramide phosphorylcholine and ceramide phosphorylethanolamine).

## Phosphoinositide‐specific phospholipase C

### Overview

Phosphoinositide‐specific phospholipase C (PLC, EC 3.1.4.11), catalyses the hydrolysis of PIP_2_ to IP_3_ and 1,2‐diacylglycerol, each of which have major second messenger functions. Two domains, X and Y, essential for catalytic activity, are conserved in the different forms of PLC. Isoforms of PLC‐*β* are activated primarily by G protein‐coupled receptors through members of the G_q/11_ family of G proteins. The receptor‐mediated activation of PLC‐*γ* involves their phosphorylation by receptor tyrosine kinases (RTK) in response to activation of a variety of growth factor receptors and immune system receptors. PLC‐*ϵ*1 may represent a point of convergence of signalling via both G protein‐coupled and catalytic receptors. Ca^2+^ ions are required for catalytic activity of PLC isoforms and have been suggested to be the major physiological form of regulation of PLC‐*δ* activity. PLC has been suggested to be activated non‐selectively by the small molecule m3M3FBS[23], although this mechanism of action has been questioned [284]. The aminosteroid U73122 has been described as an inhibitor of phosphoinositide‐specific PLC [485], although its selectivity among the isoforms is untested and it has been reported to occupy the H1 histamine receptor [235].

**Table:** 

Nomenclature	PLC*β*1	PLC*β*2	PLC*β*3	PLC*β*4	PLC*γ*1	PLC*γ*2
HGNC, UniProt	PLCB1, Q9NQ66	PLCB2, Q00722	PLCB3, Q01970	PLCB4, Q15147	PLCG1, P19174	PLCG2, P16885
Endogenous activators	G*α*q, G*α*11, G*β* *γ* [220, 399, 487]	G*α*16, G*β* *γ*, Rac2 (RAC2, P15153) [65, 236, 237, 297, 399]	G*α*q, G*β* *γ* [71, 295, 399]	G*α*q [196]	PIP_3_ [22]	PIP_3_, Rac1 (RAC1, P63000), Rac2 (RAC2, P15153), Rac3 (RAC3, P60763) [22, 411, 550]
Inhibitors	–	–	–	–	–	CCT129957 (pIC_50_ 5.5) [436]

**Table:** 

Nomenclature	PLC*δ*3	PLC*δ*4	PLC*ϵ*1	PLC*ζ*1	PLC*η*1	PLC*η*2	PLC*δ*1
HGNC, UniProt	PLCD1, P51178	PLCD3, Q8N3E9	PLCD4, Q9BRC7	PLCE1, Q9P212	PLCZ1, Q86YW0	PLCH1, Q4KWH8	PLCH2, O75038
Endogenous activators	Transglutaminase II, p122‐RhoGAP {Rat}, spermine, G*β* *γ* [199, 226, 368, 399]	–	–	Ras, rho [490, 571]	–	–	G*β* *γ* [600]
Endogenous inhibitors	Sphingomyelin [404]	–	–	–	–	–	–

### Comments

A series of PLC‐like proteins (PLCL1, Q15111; PLCL2, Q9UPR0 and PLCH1, Q4KWH8) form a family with PLC*δ* and PLC*ζ*1 isoforms, but appear to lack catalytic activity.

PLC‐*δ*2 has been cloned from bovine sources [351].

### Further reading on Phosphoinositide‐specific phospholipase C

Cocco L et al. (2015) Phosphoinositide‐specific phospholipase C in health and disease. J Lipid Res 56: 1853‐60 [PMID:25821234]

Cockcroft S et al. (2016) Topological organisation of the phosphatidylinositol 4,5‐bisphosphate‐phospholipase C resynthesis cycle: PITPs bridge the ER‐PM gap. Biochem J 473: 4289‐4310 [PMID:27888240]

Litosch I. (2015) Regulating G protein activity by lipase‐independent functions of phospholipase C. Life Sci 137: 116‐24 [PMID:26239437]

Nakamura Y et al. (2017) Regulation and physiological functions of mammalian phospholipase C. J Biochem 161: 315‐321 [PMID:28130414]

Swann K et al. (2016) The sperm phospholipase C‐zeta and Ca2+ signalling at fertilization in mammals. Biochem Soc Trans 44: 267‐72 [PMID:26862214]

## Phospholipase A_2_

### Overview

Phospholipase A_2_(PLA_2_, EC 3.1.1.4) cleaves the sn‐2 fatty acid of phospholipids, primarily phosphatidylcholine, to generate lysophosphatidylcholine and arachidonic acid. Most commonly‐used inhibitors (e.g. bromoenol lactone, arachidonyl trifluoromethyl ketone or methyl arachidonyl fluorophosphonate) are either non‐selective within the family of phospholipase A_2_ enzymes or have activity against other eicosanoid‐metabolising enzymes.

Secreted or extracellular forms: sPLA_2_‐1B, sPLA_2_‐2A, sPLA_2_‐2D, sPLA_2_‐2E, sPLA_2_‐2F, sPLA_2_‐3, sPLA_2_‐10 and sPLA_2_‐12A

Cytosolic, calcium‐dependent forms: cPLA_2_‐4A, cPLA_2_‐4B, cPLA_2_‐4C, cPLA_2_‐4D, cPLA_2_‐4E and cPLA_2_‐4F

Other forms: PLA_2_‐G5, iPLA_2_‐G6, PLA_2_‐G7 and PAFAH2 (platelet‐activating factor acetylhydrolase 2)

### Further reading on Phospholipase A2

Leslie CC. (2015) Cytosolic phospholipase A(2): physiological function and role in disease. J Lipid Res 56: 1386‐402 [PMID:25838312]

Ong WY et al. (2015) Synthetic and natural inhibitors of phospholipases A2: their importance for understanding and treatment of neurological disorders. ACS Chem Neurosci 6: 814‐31 [PMID:25891385]

Ramanadham S et al. (2015) Calcium‐independent phospholipases A2 and their roles in biological processes and diseases. J Lipid Res 56: 1643‐68 [PMID:26023050]

**Table:** 

Nomenclature	sPLA_2_‐1B	sPLA_2_‐2A	sPLA_2_‐2D	sPLA_2_‐2E	sPLA_2_‐2F	sPLA_2_‐3
HGNC, UniProt	PLA2G1B, P04054	PLA2G2A, P14555	PLA2G2D, Q9UNK4	PLA2G2E, Q9NZK7	PLA2G2F, Q9BZM2	PLA2G3, Q9NZ20

**Table:** 

Nomenclature	cPLA_2_‐4A	cPLA_2_‐4B	cPLA_2_‐4C	cPLA_2_‐4D	cPLA_2_‐4E	cPLA_2_‐4F
HGNC, UniProt	PLA2G4A, P47712	PLA2G4B, P0C869	PLA2G4C, Q9UP65	PLA2G4D, Q86XP0	PLA2G4E, Q3MJ16	PLA2G4F, Q68DD2
EC number	3.1.1.4	3.1.1.4	3.1.1.4	3.1.1.4	3.1.1.4	3.1.1.4
Inhibitors	compound 57 (pIC_50_ 8.4) [320]	–	–	–	–	–
Comments	cPLA_2_‐4A also expresses lysophospholipase (EC 3.1.1.5) activity [473].	–	–	–	–	–

**Table:** 

Nomenclature	PLA_2_‐G5	iPLA_2_‐G6	PLA_2_‐G7	sPLA_2_‐10	sPLA_2_‐12A	platelet activating factor acetylhydrolase 2
HGNC, UniProt	PLA2G5, P39877	PLA2G6, O60733	PLA2G7, Q13093	PLA2G10, O15496	PLA2G12A, Q9BZM1	PAFAH2, Q99487
EC number	3.1.1.4	3.1.1.4	3.1.1.4	3.1.1.4	3.1.1.4	3.1.1.47
Inhibitors		–	darapladib (pIC_50_ 10) [42]	–	–	–
Selective inhibitors			rilapladib (Competitive) (pIC_50_ 9.6) [568]			

### Comments

The sequence of PLA_2_‐2C suggests a lack of catalytic activity, while PLA_2_‐12B (GXIIB, GXIII sPLA_2_‐like) appears to be catalytically inactive [448]. A further fragment has been identified with sequence similarities to Group II PLA_2_ members. Otoconin 90 (OC90) shows sequence homology to PLA_2_‐G10.

A binding protein for secretory phospholipase A_2_ has been identified which shows modest selectivity for sPLA_2_‐1B over sPLA_2_‐2A, and also binds snake toxin phospholipase A_2_[13]. The binding protein appears to have clearance function for circulating secretory phospholipase A_2_, as well as signalling functions, and is a candidate antigen for idiopathic membraneous nephropathy [29].

PLA_2_‐G7 and PAFAH2 also express platelet‐activating factor acetylhydrolase activity (EC 3.1.1.47).

## Phosphatidylcholine‐specific phospholipase D

### Overview

Phosphatidylcholine‐specific phospholipase D (PLD, EC 3.1.4.4) catalyses the formation of phosphatidic acid from phosphatidylcholine. In addition, the enzyme can make use of alcohols, such as butanol in a transphosphatidylation reaction [428].

**Table:** 

Nomenclature	PLD1	PLD2
HGNC, UniProt	PLD1, Q13393	PLD2, O14939
EC number	3.1.4.4	3.1.4.4 A phosphatidylcholine + H_2_O <=> choline + a phosphatidate
Endogenous activators	ADP‐ribosylation factor 1 (ARF1, P84077), PIP_2_, RhoA, PKC evoked phosphorylation, RalA [201, 323]	ADP‐ribosylation factor 1 (ARF1, P84077), PIP_2_ [316], oleic acid [454]
Endogenous inhibitors	G*β* *γ* [418]	G*β* *γ* [418]
Inhibitors	FIPI (pIC_50_ 8) [463]	FIPI (pIC_50_ 7.8) [484]
Selective inhibitors	compound 69 (pIC_50_ 7.3) [463]	VU0364739 (pIC_50_ 7.7) [293]

### Comments

A lysophospholipase D activity (ENPP2, Q13822, also known as ectonucleotide pyrophosphatase/phosphodiesterase 2, phosphodiesterase I, nucleotide pyrophosphatase 2, autotaxin) has been described, which not only catalyses the production of lysophosphatidic acid (LPA) from lysophosphatidylcholine, but also cleaves ATP (see Goding et al., 2003 [185]). Additionally, an N‐acylethanolamine‐specific phospholipase D (NAPEPLD, Q6IQ20) has been characterized, which appears to have a role in the generation of endocannabinoids/endovanilloids, including anandamide[388]. This enzyme activity appears to be enhanced by polyamines in the physiological range [311] and fails to transphosphatidylate with alcohols [408].

Three further, less well‐characterised isoforms are PLD3 (PLD3, Q8IV08, other names Choline phosphatase 3, HindIII K4L homolog, Hu‐K4), PLD4 (PLD4, Q96BZ4, other names Choline phosphatase 4, Phosphatidylcholine‐hydrolyzing phospholipase, D4C14orf175 UNQ2488/PRO5775) and PLD5 (PLD5, Q8N7P1). PLD3 has been reported to be involved in myogenesis [391]. PLD4 is described not to have phospholipase D catalytic activity [588], but has been associated with inflammatory disorders [386, 507, 526]. Sequence analysis suggests that PLD5 is catalytically inactive.

### Further reading on Phospholipase D

Brown HA et al. (2017) Targeting phospholipase D in cancer, infection and neurodegenerative disorders. Nat Rev Drug Discov 16: 351‐367 [PMID:28209987]

Frohman MA. (2015) The phospholipase D superfamily as therapeutic targets. Trends Pharmacol Sci 36: 137‐44 [PMID:25661257]

Nelson RK et al. (2015) Physiological and pathophysiological roles for phospholipase D. J Lipid Res 56: 2229‐37 [PMID:25926691]

## Lipid phosphate phosphatases

### Overview

Lipid phosphate phosphatases, divided into phosphatidic acid phosphatases or lipins catalyse the dephosphorylation of phosphatidic acid (and other phosphorylated lipid derivatives) to generate inorganic phosphate and diacylglycerol. PTEN, a phosphatase and tensin homolog (BZS, MHAM, MMAC1, PTEN1, TEP1) is a phosphatidylinositol 3,4,5‐trisphosphate 3‐phosphatase which acts as a tumour suppressor by reducing cellular levels of PI 3,4,5‐P, thereby toning down activity of PDK1 and PKB. Loss‐of‐function mutations are frequently identified as somatic mutations in cancers.

**Table:** 

Nomenclature	Lipin1	Lipin2	Lipin3	PPA2A	PPA2B	PPA3A	phosphatase and tensin homolog
HGNC, UniProt	LPIN1, Q14693	LPIN2, Q92539	LPIN3, Q9BQK8	PLPP1, O14494	PLPP3, O14495	PLPP2, O43688	PTEN, P60484
EC number	3.1.3.4	3.1.3.4	3.1.3.4	3.1.3.4	3.1.3.4	3.1.3.4	3.1.3.67 3.1.3.48 3.1.3.16
Substrates	–	phosphatidic acid	–	–	phosphatidic acid	–	phosphatidylinositol (3,4,5)‐trisphosphate

## Phosphatidylinositol kinases

### Overview

Phosphatidylinositol may be phosphorylated at either 3‐ or 4‐ positions on the inositol ring by PI 3‐kinases or PI 4‐kinases, respectively.

### Phosphatidylinositol 3‐kinases

Phosphatidylinositol 3‐kinases (PI3K, provisional nomenclature) catalyse the introduction of a phosphate into the 3‐position of phosphatidylinositol (PI), phosphatidylinositol 4‐phosphate (PIP) or phosphatidylinositol 4,5‐bisphosphate (PIP_2_). There is evidence that PI3K can also phosphorylate serine/threonine residues on proteins. In addition to the classes described below, further serine/threonine protein kinases, including ATM (Q13315) and mTOR(P42345), have been described to phosphorylate phosphatidylinositol and have been termed PI3K‐related kinases. Structurally, PI3Ks have common motifs of at least one C2, calcium‐binding domain and helical domains, alongside structurally‐conserved catalytic domains. Wortmannin and LY 294002 are widely‐used inhibitors of PI3K activities. Wortmannin is irreversible and shows modest selectivity between Class I and Class II PI3K, while LY294002 is reversible and selective for Class I compared to Class II PI3K.

Class I PI3Ks (EC 2.7.1.153) phosphorylate phosphatidylinositol 4,5‐bisphosphate to generate phosphatidylinositol 3,4,5‐trisphosphate and are heterodimeric, matching catalytic and regulatory subunits. Class IA PI3Ks include p110*α*, p110*β* and p110*δ* catalytic subunits, with predominantly p85 and p55 regulatory subunits. The single catalytic subunit that forms Class IB PI3K is p110*γ*. Class IA PI3Ks are more associated with receptor tyrosine kinase pathways, while the Class IB PI3K is linked more with GPCR signalling.

Class II PI3Ks (EC 2.7.1.154) phosphorylate phosphatidylinositol to generate phosphatidylinositol 3‐phosphate (and possibly phosphatidylinositol 4‐phosphate to generate phosphatidylinositol 3,4‐bisphosphate). Three monomeric members exist, PI3K‐C2*α*, *β* and *β*, and include Ras‐binding, Phox homology and two C2domains.

The only class III PI3K isoform (EC 2.7.1.137) is a heterodimer formed of a catalytic subunit (VPS34) and regulatory subunit (VPS15).

### Phosphatidylinositol 4‐kinases

Phosphatidylinositol 4‐kinases (EC 2.7.1.67) generate phosphatidylinositol 4‐phosphate and may be divided into higher molecular weight type III and lower molecular weight type II forms.

## 1‐phosphatidylinositol 4‐kinase family

**Table:** 

Nomenclature	phosphatidylinositol 4‐kinase alpha	phosphatidylinositol 4‐kinase beta
HGNC, UniProt	PI4KA, P42356	PI4KB, Q9UBF8
EC number	2.7.1.67	2.7.1.67
Common abreviation	PI4KIII*α*/PIK4CA	PI4KIII*β*/PIK4CB
Endogenous activation	–	PKD‐mediated phosphorylation [212]
Sub/family‐selective inhibitors	wortmannin (pIC_50_ 6.7–6.8) [180, 352]	wortmannin (pIC_50_ 6.7–6.8) [180, 352]
Selective inhibitors	–	PIK‐93 (pIC_50_ 7.7) [26, 271]

## Phosphatidylinositol‐4‐phosphate 3‐kinase family

**Table:** 

Nomenclature	phosphatidylinositol‐4‐phosphate 3‐kinase catalytic subunit type 2 alpha	phosphatidylinositol‐4‐phosphate 3‐kinase catalytic subunit type 2 beta	phosphatidylinositol‐4‐phosphate 3‐kinase catalytic subunit type 2 gamma
HGNC, UniProt	PIK3C2A, O00443	PIK3C2B, O00750	PIK3C2G, O75747
EC number	2.7.1.154	2.7.1.154	2.7.1.154
Common abreviation	C2*α*/PIK3C2A	C2*β*/PIK3C2B	C2*γ*/PIK3C2G
Inhibitors	torin 2 (pIC_50_ 7.6) [312]	PI‐103 (pIC_50_ 8) [213]	–

### Overview

PIP_2_ is generated by phosphorylation of PI 4‐phosphate or PI 5‐phosphate by type I PI 4‐phosphate 5‐kinases or type II PI 5‐phosphate 4‐kinases.

## Phosphatidylinositol 3‐kinase family

**Table:** 

Nomenclature	phosphatidylinositol 3‐kinase catalytic subunit type 3
HGNC, UniProt	PIK3C3, Q8NEB9
EC number	2.7.1.137
Common abreviation	VPS34

## Phosphatidylinositol‐4,5‐bisphosphate 3‐kinase family

**Table:** 

Nomenclature	phosphatidylinositol‐4,5‐bisphosphate 3‐kinase catalytic subunit alpha	phosphatidylinositol‐4,5‐bisphosphate 3‐kinase catalytic subunit beta
HGNC, UniProt	PIK3CA, P42336	PIK3CB, P42338
EC number	2.7.1.153 2.7.11.1	2.7.1.153
Common abreviation	PI3K*α*	PI3K*β*
Inhibitors	PIK‐75 (pIC_50_ 9.5) [213], gedatolisib (pIC_50_ 9.4) [544], PF‐04691502 (p*K*_i_ 9.2) [309], PI‐103 (pIC_50_ 8.7) [435], BGT‐226 (pIC_50_ 8.4) [337], KU‐0060648 (pIC_50_ 8.4) [66], dactolisib (pIC_50_ 8.4) [332], apitolisib (pIC_50_ 8.3) [506]	KU‐0060648 (pIC_50_ 9.3) [66], PI‐103 (pIC_50_ 8.5) [435], AZD6482 (pIC_50_ 8) [380], ZSTK474 (pIC_50_ 7.4–7.8) [578, 583], apitolisib (pIC_50_ 7.6) [506], BGT‐226 (pIC_50_ 7.2) [337]
Sub/family‐selective inhibitors	pictilisib (pIC_50_ 8.5) [149]	pictilisib (pIC_50_ 7.5) [149]

**Table:** 

Nomenclature	phosphatidylinositol‐4,5‐bisphosphate 3‐kinase catalytic subunit gamma	phosphatidylinositol‐4,5‐bisphosphate 3‐kinase catalytic subunit delta
HGNC, UniProt	PIK3CG, P48736	PIK3CD, O00329
EC number	2.7.1.153	2.7.1.153
Common abreviation	PI3K*γ*	PI3K*δ*
Inhibitors	dactolisib (pIC_50_ 8.3) [332], apitolisib (pIC_50_ 7.8) [506], PI‐103 (pIC_50_ 7.8) [435], BGT‐226 (pIC_50_ 7.4) [337], ZSTK474 (pIC_50_ 7.3–7.3) [578, 583], TG‐100‐115 (pIC_50_ 7.1) [394], alpelisib (pIC_50_ 6.6) [164], KU‐0060648 (pIC_50_ 6.2) [66]	KU‐0060648 (pIC_50_>10) [66], idelalisib (in vitro activity against recombinant enzyme) (pIC_50_ 8.6) [290], PI‐103 (pIC_50_ 8.5) [435], ZSTK474 (pIC_50_ 8.2–8.3) [578, 583], apitolisib (pIC_50_ 8.2) [506], dactolisib (pIC_50_ 8.1) [332], alpelisib (pIC_50_ 6.5) [164]
Sub/family‐selective inhibitors	pictilisib (pIC_50_ 7.1) [149]	pictilisib (pIC_50_ 8.5) [149]
Selective inhibitors	CZC 24832 (p*K*_d_ 7.7) [32]	–

## 1‐phosphatidylinositol‐3‐phosphate 5‐kinase family

**Table:** 

Nomenclature	phosphoinositide kinase, FYVE‐type zinc finger containing
HGNC, UniProt	PIKFYVE, Q9Y2I7
EC number	2.7.1.150: ATP + 1‐phosphatidyl‐1D‐myo‐inositol 3‐phosphate = ADP + 1‐phosphatidyl‐1D‐myo‐inositol 3,5‐bisphosphate

## Type I PIP kinases (1‐phosphatidylinositol‐4‐phosphate 5‐kinase family)

### Overview

Type I PIP kinases are required for the production of the second messenger phosphatidylinositol 4,5‐bisphosphate (PtdIns(4,5)P2) by phosphorylating PtdIns(4)P [426]. This enzyme family is also known as type I PIP(5)Ks.

**Table:** 

Nomenclature	phosphatidylinositol‐4‐phosphate 5‐kinase type 1 alpha	phosphatidylinositol‐4‐phosphate 5‐kinase type 1 gamma
HGNC, UniProt	PIP5K1A, Q99755	PIP5K1C, O60331
EC number	2.7.1.68	2.7.1.68
Common abreviation	PIP5K1A	PIP5K1C
Inhibitors	ISA‐2011B [465]	–

## Type II PIP kinases (1‐phosphatidylinositol‐5‐phosphate 4‐kinase family)

### Overview

Type II PIP kinases are essential for the production of the second messenger phosphatidylinositol 4,5‐bisphosphate (PtdIns(4,5)P2) by phosphorylating PtdIns(5)P [426]. This enzyme family is also known as type II PIP(5)Ks.

**Table:** 

Nomenclature	phosphatidylinositol‐5‐phosphate 4‐kinase type 2 alpha	phosphatidylinositol‐5‐phosphate 4‐kinase type 2 beta	phosphatidylinositol‐5‐phosphate 4‐kinase type 2 gamma
HGNC, UniProt	PIP4K2A, P48426	PIP4K2B, P78356	PIP4K2C, Q8TBX8
EC number	2.7.1.149 ATP + 1‐phosphatidyl‐1D‐myo‐inositol 5‐phosphate <=> ADP + 1‐phosphatidyl‐1D‐myo‐inositol 4,5‐bisphosphate	2.7.1.149	2.7.1.149
Common abreviation	PIP4K2A	PIP4K2B	PIP4K2C

### Further reading on Phosphatidylinositol kinases

Bauer TM et al. (2015) Targeting PI3 kinase in cancer. Pharmacol Ther 146: 53‐60 [PMID:25240910]

Mayer IA et al. (2016) The PI3K/AKT Pathway as a Target for Cancer Treatment. Annu Rev Med 67: 11‐28 [PMID:26473415]

Singh P et al. (2016) p110alpha and p110beta isoforms of PI3K signaling: are they two sides of the same coin? FEBS Lett 590: 3071‐82 [PMID:27552098]

Zhu J et al. (2015) Discovery of selective phosphatidylinositol 3‐kinase inhibitors to treat hematological malignancies. Drug Discov Today 20: 988‐94 [PMID:25857437]

### Further reading on Glycerophospholipid turnover

Cauvin C et al. (2015) Phosphoinositides: Lipids with informative heads and mastermind functions in cell division. Biochim Biophys Acta 1851: 832‐43 [PMID:25449648]

Irvine RF. (2016) A short history of inositol lipids. J Lipid Res 57: 1987‐1994 [PMID:27623846]

Poli A et al. (2016) Nuclear Phosphatidylinositol Signaling: Focus on Phosphatidylinositol Phosphate Kinases and Phospholipases C. J Cell Physiol 231: 1645‐55 [PMID:26626942]

## Haem oxygenase

### Overview

Haem oxygenase (heme,hydrogen‐donor:oxygen oxidoreductase (*α*‐methene‐oxidizing, hydroxylating)), E.C. 1.14.99.3, converts heme into biliverdin and carbon monoxide, utilizing NADPH as cofactor.

**Table:** 

Nomenclature	Haem oxygenase 1	Haem oxygenase 2
HGNC, UniProt	HMOX1, P09601	HMOX2, P30519
EC number	1.14.14.18 Protoheme + 3 [reduced NADPH‐hemoprotein reductase] + 3 O(2) <=> biliverdin + Fe(2+) + CO + 3 [oxidized NADPH‐hemoprotein reductase] + 3 H(2)O	1.14.14.18 Protoheme + 3 [reduced NADPH–hemoprotein reductase] + 3 O(2) <=> biliverdin + Fe(2+) + CO + 3 [oxidized NADPH–hemoprotein reductase] + 3 H(2)O
Common abreviation	HO1	HO2

### Comments

The existence of a third non‐catalytic version of haem oxygenase, HO3, has been proposed, although this has been suggested to be a pseudogene [215]. The chemical tin protoporphyrin IX acts as a haem oxygenase inhibitor in rat liver with an IC_50_ value of 11 nM [128].

### Further reading on Haem oxygenase

Abraham NG et al. (2016) Translational Significance of Heme Oxygenase in Obesity and Metabolic Syndrome. Trends Pharmacol Sci 37: 17‐36 [PMID:26515032]

Naito Y et al. (2014) Heme oxygenase‐1 and anti‐inflammatory M2 macrophages. Arch Biochem Biophys 564: 83‐8 [PMID:25241054]

Otterbein LE et al. (2016) Heme Oxygenase‐1 and Carbon Monoxide in the Heart: The Balancing Act Between Danger Signaling and Pro‐Survival. Circ Res 118: 1940‐59 [PMID:27283533]

Poulos TL. (2014) Heme enzyme structure and function. Chem. Rev. 114: 3919‐62 [PMID:24400737]

## Hydrogen sulphide synthesis

### Overview

Hydrogen sulfide is a gasotransmitter, with similarities to nitric oxide and carbon monoxide. Although the enzymes indicated below have multiple enzymatic activities, the focus here is the generation of hydrogen sulphide (H_2_S) and the enzymatic characteristics are described accordingly. Cystathionine *β*‐synthase (CBS) and cystathionine *γ*‐lyase (CSE) are pyridoxal phosphate (PLP)‐dependent enzymes. 3‐mercaptopyruvate sulfurtransferase (3‐MPST) functions to generate H_2_S; only CAT is PLP‐dependent, while 3‐MPST is not. Thus, this third pathway is sometimes referred to as PLP‐independent. CBS and CSE are predominantly cytosolic enzymes, while 3‐MPST is found both in the cytosol and the mitochondria.

**Table:** 

Nomenclature	Cystathionine *β*‐synthase	Cystathionine *γ*‐lyase	L‐Cysteine:2‐oxoglutarate aminotransferase	3‐Mercaptopyruvate sulfurtransferase
HGNC, UniProt	CBS, P35520	CTH, P32929	KYAT1, Q16773	MPST, P25325
EC number	4.2.1.22	4.4.1.1	4.4.1.13	2.8.1.2
Common abreviation	CBS	CSE	CAT	MPST
Endogenous substrates	L‐cysteine (*K*_m_6×10^−3^M) [81], L‐homocysteine	L‐cysteine	L‐cysteine	3‐mercaptopyruvic acid (*K*_m_1.2×10^−3^M) [369]
Products	cystathionine	NH_3_, pyruvic acid	NH_3_, pyruvic acid	pyruvic acid
Cofactors	pyridoxal phosphate	pyridoxal phosphate	pyridoxal phosphate	Zn^2+^
Inhibitors	aminooxyacetic acid (pIC_50_ 5.1) [17]	aminoethoxyvinylglycine (pIC_50_ 6) [17], aminooxyacetic acid (pIC_50_ 6) [17], *β*‐Cyano‐L‐alanine (pIC_50_ 5.8) [17], propargylglycine (pIC_50_ 4.4) [17]	–	–

### Further reading on Hydrogen sulphide synthesis

Asimakopoulou A et al. (2013) Selectivity of commonly used pharmacological inhibitors for cystathionine á synthase (CBS) and cystathionine g lyase (CSE). British Journal of Pharmacology 169: 922‐932 [PM:23488457]

Kanagy NL et al. (2017) Vascular biology of hydrogen sulfide. Am J Physiol Cell Physiol 312: C537‐C549 [PMID:28148499]

Meng G et al. (2017) Protein S‐sulfhydration by hydrogen sulfide in cardiovascular system. Br J Pharmacol [PMID:28148499]

Wallace JL et al. (2015) Hydrogen sulfide‐based therapeutics: exploiting a unique but ubiquitous gasotransmitter. Nat Rev Drug Discov 14: 329‐45 [PMID:28148499]

## Hydrolases

### Overview

Listed in this section are hydrolases not accumulated in other parts of the Concise Guide, such as monoacylglycerol lipase and acetylcholinesterase. Pancreatic lipase is the predominant mechanism of fat digestion in the alimentary system; its inhibition is associated with decreased fat absorption. CES1 is present at lower levels in the gut than CES2 (P23141), but predominates in the liver, where it is responsible for the hydrolysis of many aliphatic, aromatic and steroid esters. Hormone‐sensitive lipase is also a relatively non‐selective esterase associated with steroid ester hydrolysis and triglyceride metabolism, particularly in adipose tissue. Endothelial lipase is secreted from endothelial cells and regulates circulating cholesterol in high density lipoproteins.

**Table:** 

Nomenclature	pancreatic lipase	lipase G, endothelial type	carboxylesterase 1	lipase E, hormone sensitive type
HGNC, UniProt	PNLIP, P16233	LIPG, Q9Y5X9	CES1, P23141	LIPE, Q05469
EC number	3.1.1.3	3.1.1.3	3.1.1.1	3.1.1.79
Common abreviation	PNLIP	LIPG	CES1	LIPE
Inhibitors	orlistat (pIC_50_ 8.9) [61]	–	–	–

**Table:** 

Nomenclature	ectonucleoside triphosphate diphosphohydrolase 1	ectonucleoside triphosphate diphosphohydrolase 2
Systematic nomenclature	CD39	CD39L1
HGNC, UniProt	ENTPD1, P49961	ENTPD2, Q9Y5L3
EC number	3.6.1.5 Hydrolyzes NTPs to nucleotide monophosphates (NMPs): A nucleoside 5'‐triphosphate + 2 H_2_O <=> a nucleoside 5'‐phosphate + 2 phosphate	3.6.1.‐ Hydrolyzes extracellular nucleotide 5'‐triphosphates: NTP>NMP + 2 phosphate
Common abreviation	NTPDase‐1	NTPDase‐2
Selective inhibitors	–	PSB‐6426 (p*K*_i_ 5.1) [53]
Comments	ENTPD1 sequentially converts extracellular purine nucleotides (ATP and ADP) to the monophosphate form. Adenosine is then generated by the action of Ecto‐5'‐Nucleotidase (CD73). ENTPD1 is the rate‐limiting step. Extracellular ATP acts as a damage‐associated molecular pattern (DAMP) that activates innate immune cells through adenosine‐induced activation of P2X and P2Y purinogenic receptors.	–

### Further reading on Hydrolases

Markey GM. (2011) Carboxylesterase 1 (Ces1): from monocyte marker to major player. J. Clin. Pathol. 64: 107‐9 [PMID:21177752]

Takenaka MC et al. (2016) Regulation of the T Cell Response by CD39. Trends Immunol 37: 427‐39 [PMID:27236363]

## Inositol phosphate turnover

### Overview

The sugar alcohol D‐myo‐inositol is a component of the phosphatidylinositol signalling cycle, where the principal second messenger is inositol 1,4,5‐trisphosphate, IP_3_, which acts at intracellular ligand‐gated ion channels, IP_3_ receptors to elevate intracellular calcium. IP_3_ is recycled to inositol by phosphatases or phosphorylated to form other active inositol polyphosphates. Inositol produced from dephosphorylation of IP_3_ is recycled into membrane phospholipid under the influence of phosphatidyinositol synthase activity (CDP‐diacylglycerol‐inositol 3‐phosphatidyltransferase [EC 2.7.8.11]).

## Inositol 1,4,5‐trisphosphate 3‐kinases

### Overview

Inositol 1,4,5‐trisphosphate 3‐kinases (E.C. 2.7.1.127, ENSFM00250000001260) catalyse the generation of inositol 1,3,4,5‐tetrakisphosphate (IP_4_) from IP_3_. IP_3_ kinase activity is enhanced in the presence of calcium/calmodulin(CALM1 CALM2 CALM3, P62158) [98].

Information on members of this family may be found in the online database.

## Inositol polyphosphate phosphatases

### Overview

Members of this family exhibit phosphatase activity towards IP_3_, as well as towards other inositol derivatives, including the phospholipids PIP_2_ and PIP_3_. With IP_3_ as substrate, 1‐phosphatase (EC 3.1.3.57) generates 4,5,‐IP_2_, 4‐phosphatases (EC 3.1.3.66, ENSFM00250000001432) generate 1,5,‐IP_2_ and 5‐phosphatases (E.C. 3.1.3.36 or 3.1.3.56) generate 1,4,‐IP_2_.

Information on members of this family may be found in the online database.

### Comments

In vitro analysis suggested IP_3_ and IP_4_ were poor substrates for SKIP, synaptojanin 1 and synaptojanin 2, but suggested that PIP_2_ and PIP_3_ were more efficiently hydrolysed [458].

## Inositol monophosphatase

### Overview

Inositol monophosphatase (E.C. 3.1.3.25, IMPase, myo‐inositol‐1(or 4)‐phosphate phosphohydrolase) is a magnesium‐dependent homodimer which hydrolyses myo‐inositol monophosphate to generate myo‐inositol and phosphate. Glycerol may be a physiological phosphate acceptor. Li^+^ is a nonselective un‐competitive inhibitor more potent at IMPase 1 (pK_i_ca. 3.5, [347]; pIC_50_ 3.2, [385]) than IMPase 2 (pIC_50_ 1.8‐2.1, [385]). IMPase activity may be inhibited competitively by L690330(pK_i_ 5.5, [347]), although the enzyme selectivity is not yet established.

**Table:** 

Nomenclature	IMPase 1	IMPase 2
HGNC, UniProt	IMPA1, P29218	IMPA2, O14732
EC number	3.1.3.25	3.1.3.25
Rank order of affinity	inositol 4‐phosphate>inositol 3‐phosphate>inositol 1‐phosphate [347]	–
Inhibitors	Li^+^ (p*K*_i_ 3.5) [347]	–

### Comments

Polymorphisms in either of the genes encoding these enzymes have been linked with bipolar disorder [481, 482, 589]. Disruption of the gene encoding IMPase 1, but not IMPase 2, appears to mimic the effects of Li^+^ in mice [104, 105].

### Further reading on Inositol phosphate turnover

Irvine R. (2016) A tale of two inositol trisphosphates. Biochem Soc Trans 44: 202‐11 [PMID:26862207]

Livermore TM et al. (2016) Phosphate, inositol and polyphosphates. Biochem Soc Trans 44: 253‐9 [PMID:26862212]

Miyamoto A et al. (2017) Probes for manipulating and monitoring IP3. Cell Calcium 64: 57‐64 [PMID:27887748]

Windhorst S et al. (2017) Inositol‐1,4,5‐trisphosphate 3‐kinase‐A (ITPKA) is frequently over‐expressed and functions as an oncogene in several tumor types. Biochem Pharmacol 137: 1‐9 [PMID:28377279]

## Lanosterol biosynthesis pathway

### Overview

Lanosterol is a precursor for cholesterol, which is synthesized primarily in the liver in a pathway often described as the mevalonate or HMG‐CoA reductase pathway. The first two steps (formation of acetoacetyl CoA and the mitochondrial generation of (S)‐3‐hydroxy‐3‐methylglutaryl‐CoA) are also associated with oxidation of fatty acids.

**Table:** 

Nomenclature	acetyl‐CoA acetyltransferase 1	acetyl‐CoA acetyltransferase 2	hydroxymethylglutaryl‐CoA synthase 1	hydroxymethylglutaryl‐CoA synthase 2
HGNC, UniProt	ACAT1, P24752	ACAT2, Q9BWD1	HMGCS1, Q01581	HMGCS2, P54868
EC number	2.3.1.9: 2acetyl CoA = acetoacetyl CoA + coenzyme A	2.3.1.9: 2acetyl CoA = acetoacetyl CoA + coenzyme A	2.3.3.10: acetyl CoA + H_2_O + acetoacetyl CoA ‐>(S)‐3‐hydroxy‐3‐methylglutaryl‐CoA + coenzyme A	2.3.3.10: acetyl CoA + H_2_O + acetoacetyl CoA ‐>(S)‐3‐hydroxy‐3‐methylglutaryl‐CoA + coenzyme A
Comments	–	–	HMGCoA synthase is found in cytosolic (HMGCoA synthase 1) and mitochondrial (HMGCoA synthase 2) versions; the former associated with (R)‐mevalonate synthesis and the latter with ketogenesis.	HMGCoA synthase is found in cytosolic (HMGCoA synthase 1) and mitochondrial (HMGCoA synthase 2) versions; the former associated with (R)‐mevalonate synthesis and the latter with ketogenesis.

**Table:** 

Nomenclature	hydroxymethylglutaryl‐CoA reductase	mevalonate kinase	phosphomevalonate kinase	diphosphomevalonate decarboxylase
HGNC, UniProt	HMGCR, P04035	MVK, Q03426	PMVK, Q15126	MVD, P53602
EC number	1.1.1.34: (S)‐3‐hydroxy‐3‐methylglutaryl‐CoA + NADPH ‐>(R)‐mevalonate + coenzyme A + NADP^+^ Reaction mechanism:: First step: (S)‐3‐hydroxy‐3‐methylglutaryl‐CoA + NADPH ‐> mevaldyl‐CoA + NADP^+^ Second step: mevaldyl‐CoA + H2O ‐> (R)‐mevalonate + NADP^+^	2.7.1.36: ATP + (R)‐mevalonate ‐>ADP + (R)‐5‐phosphomevalonate	2.7.4.2: ATP + (R)‐5‐phosphomevalonate = ADP + (R)‐5‐diphosphomevalonate	4.1.1.33: ATP + (R)‐5‐diphosphomevalonate ‐>ADP + isopentenyl diphosphate + CO_2_ + PO_3_^4‐^
Inhibitors	lovastatin (Competitive) (p*K*_i_ 9.2) [10], rosuvastatin (Competitive) (pIC_50_ 8.3) [241], cerivastatin (Competitive) (p*K*_i_ 8.2) [67], atorvastatin (Competitive) (pIC_50_ 8.1) [241], cerivastatin (Competitive) (pIC_50_ 8) [528], simvastatin (Competitive) (pIC_50_ 8) [241], fluvastatin (Competitive) (pIC_50_ 7.6) [241]	–	–	–
Comments	HMGCoA reductase is associated with intracellular membranes; enzymatic activity is inhibited by phosphorylation by AMP‐activated kinase. The enzymatic reaction is a three‐step reaction involving the intermediate generation of mevaldehyde‐CoA and mevaldehyde.	Mevalonate kinase activity is regulated by the downstream products farnesyl diphosphate and geranyl diphosphate as an example of feedback inhibition.	–	–

**Table:** 

Nomenclature	isopentenyl‐diphosphate Δ‐isomerase 1	isopentenyl‐diphosphate Δ‐isomerase 2	geranylgeranyl diphosphate synthase
HGNC, UniProt	IDI1, Q13907	IDI2, Q9BXS1	GGPS1, O95749
EC number	5.3.3.2: isopentenyl diphosphate = dimethylallyl diphosphate	5.3.3.2: isopentenyl diphosphate = dimethylallyl diphosphate	2.5.1.29: trans,trans‐farnesyl diphosphate + isopentenyl diphosphate ‐>geranylgeranyl diphosphate + diphosphate 2.5.1.10: geranyl diphosphate + isopentenyl diphosphate ‐>trans,trans‐farnesyl diphosphate + diphosphate 2.5.1.1: dimethylallyl diphosphate + isopentenyl diphosphate = geranyl diphosphate + diphosphate

**Table:** 

Nomenclature	farnesyl diphosphate synthase	squalene synthase	squalene monooxygenase	lanosterol synthase
HGNC, UniProt	FDPS, P14324	FDFT1, P37268	SQLE, Q14534	LSS, P48449
EC number	2.5.1.10: geranyl diphosphate + isopentenyl diphosphate ‐>trans,trans‐farnesyl diphosphate + diphosphate 2.5.1.1: dimethylallyl diphosphate + isopentenyl diphosphate = geranyl diphosphate + diphosphate	2.5.1.21: 2trans,trans‐farnesyl diphosphate ‐>presqualene diphosphate + diphosphate presqualene diphosphate + NAD(P)H + H^+^ ‐>squalene + diphosphate + NAD(P)^+^	1.14.13.132: H^+^ + NADPH + O_2_ + squalene = H_2_O + NADP^+^ + (S)‐2,3‐epoxysqualene	5.4.99.7: (S)‐2,3‐epoxysqualene = lanosterol
Cofactors	–	NADPH	–	–
Inhibitors	risedronate (pIC_50_ 8.4) [33], zoledronic acid (p*K*_i_ 7.1) [129], alendronate (pIC_50_ 6.3) [33]	zaragozic acid A (p*K*_i_ 10.1) [34] – Rat, zaragozic acid A (pIC_50_ 9.2) [530]		–
Selective inhibitors	ibandronic acid (p*K*_i_ 6.7) [129], pamidronic acid (pIC_50_ 6.7) [129]	–	–	–

### Further reading on Lanosterol biosynthesis pathway

Moutinho M et al. (2017) The mevalonate pathway in neurons: It's not just about cholesterol. Exp Cell Res [PMID:28232115]

Mullen PJ et al. (2016) The interplay between cell signalling and the mevalonate pathway in cancer. Nat Rev Cancer 16: 718‐731 [PMID:27562463]

Ness GC. (2015) Physiological feedback regulation of cholesterol biosynthesis: Role of translational control of hepatic HMG‐CoA reductase and possible involvement of oxylanosterols. Biochim Biophys Acta 1851: 667‐73 [PMID:25701719]

Porter TD. (2015) Electron Transfer Pathways in Cholesterol Synthesis. Lipids 50: 927‐36 [PMID:26344922]

Samaras K et al. (2016) Does statin use cause memory decline in the elderly? Trends Cardiovasc Med 26: 550‐65 [PMID:27177529]

## Nucleoside synthesis and metabolism

### Overview

The de novo synthesis and salvage of nucleosides have been targetted for therapeutic advantage in the treatment of particular cancers and gout. Dihydrofolate reductase produces tetrahydrofolate, a cofactor required for synthesis of purines, pyrimidines and amino acids. GART allows formylation of phosphoribosylglycinamide, an early step in purine biosynthesis. Dihydroorotate dehydrogenase produces orotate, a key intermediate in pyrimidine synthesis. IMP dehydrogenase generates xanthosine monophosphate, an intermediate in GTP synthesis.

**Table:** 

Nomenclature	dihydrofolate reductase	dihydroorotate dehydrogenase (quinone)	inosine monophosphate dehydrogenase 1	inosine monophosphate dehydrogenase 2	xanthine dehydrogenase
HGNC, UniProt	DHFR, P00374	DHODH, Q02127	IMPDH1, P20839	IMPDH2, P12268	XDH, P47989
EC number	1.5.1.3	1.3.5.2	1.1.1.205	1.1.1.205	1.17.1.4
Inhibitors	pemetrexed (p*K*_i_ 8.1) [171, 474], pralatrexate (p*K*_i_ 7.3) [244]	teriflunomide (p*K*_i_ 7.5) [214], leflunomide (p*K*_i_ 4.9) [397]	mycophenolic acid (pIC_50_ 7.7) [376], ribavirin (pIC_50_ 5.6–6) [572], thioguanine [132, 546]	mycophenolic acid (pIC_50_ 7.7) [376], ribavirin (pIC_50_ 5.6–6) [572], thioguanine [132, 546]	febuxostat (p*K*_i_ 9.9) [387] – Bovine, allopurinol (p*K*_i_ 5.2) [36]
Selective inhibitors	methotrexate (p*K*_i_ 8.9) [446]	–	–	–	–

**Table:** 

Nomenclature	ribonucleotide reductase catalytic subunit M1	ribonucleotide reductase regulatory subunit M2	ribonucleotide reductase regulatory TP53 inducible subunit M2B	thymidylate synthetase	phosphoribosylglycinamide formyltransferase, phosphoribosylglycinamide synthetase, phosphoribosylaminoimidazole synthetase	purine nucleoside phosphorylase
HGNC, UniProt	RRM1, P23921	RRM2, P31350	RRM2B, Q7LG56	TYMS, P04818	GART, P22102	PNP, P00491
EC number	1.17.14.1	1.17.4.1	1.17.1.4	2.1.1.45	2.1.2.2 6.3.3.1 6.3.4.13	–
Common abreviation	–	–	–	–	GART	–
Inhibitors	clofarabine(pIC_50_ 8.3) [400], fludarabine(pIC_50_ 6) [534], hydroxyurea(pIC_50_ 3.8) [471], gemcitabine[219]		–	pemetrexed (p*K*_i_ 7) [474], capecitabine [69, 398]	pemetrexed (p*K*_i_ 5) [474] – Mouse	–
Selective inhibitors	–	–	–	raltitrexed (pIC_50_ 6.5) [172]	–	–

### Comments

Thymidylate synthetase allows the interconversion of dUMP and dTMP, thereby acting as a crucial step in DNA synthesis. Purine nucleoside phosphorylase allows separation of a nucleoside into the nucleobase and ribose phosphate for nucleotide salvage. Xanthine dehydrogenase generates urate in the purine degradation pathway. Post‐translational modifications of xanthine dehydrogenase convert the enzymatic reaction to a xanthine oxidase, allowing the interconversion of hypoxanthine and xanthine, with the production (or consumption) of reactive oxygen species. Ribonucleotide reductases allow the production of deoxyribonucleotides from ribonucleotides.

### Further reading on Nucleoside synthesis and metabolism

Day RO et al. (2016) Xanthine oxidoreductase and its inhibitors: relevance for gout. Clin Sci (Lond) 130: 2167‐2180 [PMID:27798228]

Okafor ON et al. (2017) Allopurinol as a therapeutic option in cardiovascular disease. Pharmacol Ther 172: 139‐150 [PMID:27916655]

Sramek M et al. (2017) Much more than you expected: The non‐DHFR‐mediated effects of methotrexate. Biochim Biophys Acta 1861: 499‐503 [PMID:27993660]

## Sphingosine 1‐phosphate turnover

### Overview

S1P (sphingosine 1‐phosphate) is a pro‐survival signal, in contrast to ceramide. It is formed by the sphingosine kinase‐catalysed phosphorylation of sphingosine. S1P can be released from cells to act as an agonist at a family of five G protein‐coupled receptors (S1P_1‐5_) but also has intracellular targets. S1P can be dephosphorylated back to sphingosine or hydrolysed to form hexadecanal and phosphoethanolamine. Sphingosine choline phosphotransferase (EC 2.7.8.10) generates sphingosylphosphocholine from sphingosine and CDP‐choline. Sphingosine *β*‐galactosyltransferase (EC 2.4.1.23) generates psychosine from sphingosine in the presence of UDP‐*α*‐D‐galactose. The molecular identities of these enzymes have not been confirmed.

## Sphingosine kinase

**Table:** 

Nomenclature	sphingosine kinase 1	sphingosine kinase 2
HGNC, UniProt	SPHK1, Q9NYA1	SPHK2, Q9NRA0
EC number	2.7.1.91: sphingosine + ATP = sphingosine 1‐phosphate + ADP ATP + sphinganine = sphinganine 1‐phosphate + ADP	2.7.1.91: sphingosine + ATP = sphingosine 1‐phosphate + ADP ATP + sphinganine = sphinganine 1‐phosphate + ADP
Common abreviation	SPHK1	SPHK2
Cofactors	Mg^2+^ [469]	Mg^2+^
Sub/family‐selective inhibitors	SKI‐II (pIC_50_ 6.3) [156]	–
Selective inhibitors	PF‐543 (pIC8.7) [556],	ABC294640 (p*K*_i_ 5) [157], ROMe (pIC_50_ 4.6) [304]

### Further reading on Sphingosine kinases

Adams DR et al. (2016) Sphingosine Kinases: Emerging Structure‐Function Insights. Trends Biochem Sci 41: 395‐409 [PMID:27021309]

Marfe G et al. (2015) Sphingosine kinases signalling in carcinogenesis. Mini Rev Med Chem 15: 300‐14 [PMID:25723458]

Pyne NJ et al. (2017) Sphingosine Kinase 2 in Autoimmune/Inflammatory Disease and the Development of Sphingosine Kinase 2 Inhibitors. Trends Pharmacol Sci 38: 581‐591 [PMID:28606480]

Pyne S et al. (2016) Sphingosine 1‐phosphate and sphingosine kinases in health and disease: Recent advances. Prog Lipid Res 62: 93‐106 [PMID:26970273]

Santos WL et al. (2015) Drugging sphingosine kinases. ACS Chem Biol 10: 225‐33 [PMID:25384187]

## Sphingosine 1‐phosphate phosphatase

**Table:** 

Nomenclature	sphingosine‐1‐phosphate phosphatase 1	sphingosine‐1‐phosphate phosphatase 2
HGNC, UniProt	SGPP1, Q9BX95	SGPP2, Q8IWX5
EC number	3.1.3.‐: sphingosine 1‐phosphate ‐>sphingosine + inorganic phosphate	3.1.3.‐: sphingosine 1‐phosphate ‐>sphingosine + inorganic phosphate
Common abreviation	SGPP1	SGPP2
Comments	Depletion of S1P phosphohydrolase‐1 (SPP1), which degrades intracellular S1P, induces the unfolded protein response and endoplasmic reticulum stress‐induced autophagy [231].	–

## Sphingosine 1‐phosphate lyase

**Table:** 

Nomenclature	sphingosine‐1‐phosphate lyase 1
HGNC, UniProt	SGPL1, O95470
EC number	4.1.2.27: sphingosine 1‐phosphate ‐>phosphoethanolamine + hexadecanal
Cofactors	pyridoxal phosphate
Inhibitors	compound 31 (pIC_50_ 6.7) [564]
Comments	THI (2‐Acetyl‐5‐tetrahydroxybutyl imidazole) inhibits the enzyme activity in intact cell preparations [462].

### Further reading on Sphingosine 1‐phosphate lyase

Ebenezer DL et al. (2016) Targeting sphingosine‐1‐phosphate signaling in lung diseases. Pharmacol Ther 168: 143‐157 [PMID:27621206]

Sanllehi P et al. (2016) Inhibitors of sphingosine‐1‐phosphate metabolism (sphingosine kinases and sphingosine‐1‐phosphate lyase). Chem Phys Lipids 197: 69‐81 [PMID:26200919]

## Thyroid hormone turnover

### Overview

The thyroid hormones triiodothyronine and thyroxine, usually abbreviated as triiodothyronine and T_4_, respectively, are synthesized in the thyroid gland by sequential metabolism of tyrosine residues in the glycosylated homodimeric protein thyroglobulin (TG, P01266) under the influence of the haem‐containing protein iodide peroxidase. Iodide peroxidase/TPO is a haem‐containing enzyme, from the same structural family as eosinophil peroxidase (EPX, P11678), lactoperoxidase (LPO, P22079) and myeloperoxidase (MPO, P05164). Circulating thyroid hormone is bound to thyroxine‐binding globulin (SERPINA7, P05543).

**Table:** 

Nomenclature	thyroid peroxidase
HGNC, UniProt	TPO, P07202
EC number	1.11.1.8: [Thyroglobulin]‐L‐tyrosine + H_2_O_2_ + H^+^ + I^‐^ ‐> [Thyroglobulin]‐3,5,3'‐triiodo‐L‐thyronine + [thyroglobulin]‐aminoacrylate + H_2_O
Common abreviation	TPO
Cofactors	Ca^2+^
Inhibitors	methimazole [373], propylthiouracil [373]
Comments	Carbimazole is a pro‐drug for methimazole

### Tissue deiodinases

These are 1 TM selenoproteins that remove an iodine from T_4_(3,3',5,5'‐tetraiodothyronine) to generate triiodothyronine(3,3',5‐triiodothyronine, a more potent agonist at thyroid hormone receptors) or rT_3_(rT3, 3,3',5'‐triiodothyronine, a relatively inactive analogue). DIO1 is also able to deiodinate RT3 to form 3,3'‐diidothyronine (T_2_). Iodotyrosine deiodinase is a 1TM homodimeric enzyme.

**Table:** 

Nomenclature	iodothyronine deiodinase 1	iodothyronine deiodinase 2	iodothyronine deiodinase 3	iodotyrosine deiodinase
HGNC, UniProt	DIO1, P49895	DIO2, Q92813	DIO3, P55073	IYD, Q6PHW0
EC number	1.97.1.10: T_4_ ‐>triiodothyronine rT_3_ ‐>T_2_	1.97.1.10: T_4_ ‐>triiodothyronine rT_3_ ‐>T_2_	1.97.1.11: T_4_ ‐>triiodothyronine rT_3_ ‐>T_2_	1.22.1.1: 3‐iodotyrosine ‐>L‐tyrosine + I^‐^ 3,5‐diiodo‐L‐tyrosine ‐>3‐iodotyrosine + I^‐^
Common abreviation	DIO1	DIO2	DIO3	IYD
Cofactors	–	–	–	flavin adenine dinucleotide, NADPH

### Further reading on Thyroid hormone turnover

Darras VM et al. (2015) Intracellular thyroid hormone metabolism as a local regulator of nuclear thyroid hormone receptor‐mediated impact on vertebrate development. Biochim. Biophys. Acta 1849: 130‐41 [PMID:24844179]

Gereben B et al. (2015) Scope and limitations of iodothyronine deiodinases in hypothyroidism. Nat Rev Endocrinol 11: 642‐52 [PMID:26416219]

Mondal S et al. (2017) Novel thyroid hormone analogues, enzyme inhibitors and mimetics, and their action. Mol Cell Endocrinol [PMID:28408161]

Schweizer U et al. (2015) New insights into the structure and mechanism of iodothyronine deiodinases. J Mol Endocrinol 55: R37‐52 [PMID:26390881]

van der Spek AH et al. (2017) Thyroid hormone metabolism in innate immune cells. J Endocrinol 232: R67‐R81 [PMID:27852725]

## 1.14.11.29 2‐oxoglutarate oxygenases

### Overview

Hypoxia inducible factor (HIF) is a transcriptional complex that is involved in oxygen homeostasis [466]. At normal oxygen levels, the alpha subunit of HIF (HIF‐1*α*) is targeted for degradation by prolyl hydroxylation by the prolyl hydrolxyases PHD proteins 1‐3 (HIF‐PHs) whch are 2‐oxoglutarate oxygenases responsible for the post‐translational modification of a specific proline in each of the oxygen‐dependent degradation domains of HIF‐1*α*. Hydroxylated HIFs are then targeted for proteasomal degradation via the von Hippel‐Lindau ubiquitination complex [245]. Under hypoxic conditions, the hydroxylation reaction is blunted which results in decreased HIF degradation. The surviving HIFs are then available to translocate to the nucleus where they heterodimerize with HIF‐1*β*, effecting increased expression of hypoxia‐inducible genes.

HIF‐PH enzymes are being investigated as pharmacological targets as their inhibition mimics the hypoxic state and switches on transcription of genes associated with processes such as erythropoiesis and vasculogenesis [151]. Small molecule HIF‐PH inhibitors are in clinical trial as novel therapies for the amelioration of anemia associated with chronic kidney disease [50].

**Table:** 

Nomenclature	egl‐9 family hypoxia inducible factor 2	egl‐9 family hypoxia inducible factor 1	egl‐9 family hypoxia inducible factor 3
HGNC, UniProt	EGLN2, Q96KS0	EGLN1, Q9GZT9	EGLN3, Q9H6Z9
EC number	–	1.14.11.29	1.14.11.29
Common abreviation	PHD1	PHD2	PHD3
Inhibitors		IOX2 (pIC_50_ 7.7) [91]	

### Further reading on 2‐oxoglutarate oxygenases

Ivan M et al. (2017) The EGLN‐HIF O2‐Sensing System: Multiple Inputs and Feedbacks. Mol Cell 66: 772‐779 [PMID:28622522]

Markolovic S et al. (2015) Protein Hydroxylation Catalyzed by 2‐Oxoglutarate‐dependent Oxygenases. J Biol Chem 290: 20712‐22 [PMID:26152730]

Salminen A et al. (2015) 2‐Oxoglutarate‐dependent dioxygenases are sensors of energy metabolism, oxygen availability, and iron homeostasis: potential role in the regulation of aging process. Cell Mol Life Sci 72: 3897‐914 [PMID:26118662]

Wu Y et al. (2017) Application of in‐vitro screening methods on hypoxia inducible factor prolyl hydroxylase inhibitors. Bioorg Med Chem 25: 3891‐3899 [PMID:28625716]

Zurlo G et al. (2016) New Insights into Protein Hydroxylation and Its Important Role in Human Diseases. Biochim Biophys Acta 1866: 208‐220 [PMID:27663420]

## 1.14.13.9 kynurenine 3‐monooxygenase

**Table:** 

Nomenclature	Kynurenine 3‐monooxygenase
HGNC, UniProt	KMO, O15229
EC number	1.14.13.9 L‐kynurenine + NADPH + O_2_<=> 3‐hydroxy‐L‐kynurenine + NADP(+) + H_2_O
Comments	Kynurenine 3‐monooxygenase participates in metabolism of the essential amino acid tryptophan.

### Further reading on Kynurenine 3‐monooxygenases

Dounay AB et al. (2015) Challenges and Opportunities in the Discovery of New Therapeutics Targeting the Kynurenine Pathway. J Med Chem 58: 8762‐82 [PMID:26207924]

Erhardt S et al. (2017) The kynurenine pathway in schizophrenia and bipolar disorder. Neuropharmacology 112: 297‐306 [PMID:27245499]

Fujigaki H et al. (2017) L‐Tryptophan‐kynurenine pathway enzymes are therapeutic target for neuropsychiatric diseases: Focus on cell type differences. Neuropharmacology 112: 264‐274 [PMID:26767951]

Smith JR et al. (2016) Kynurenine‐3‐monooxygenase: a review of structure, mechanism, and inhibitors. Drug Discov Today 21: 315‐24 [PMID:26589832]

Song P et al. (2017) Abnormal kynurenine pathway of tryptophan catabolism in cardiovascular diseases. Cell Mol Life Sci 74: 2899‐2916 [PMID:28314892]

## 2.4.2.30 poly(ADP‐ribose)polymerases

### Overview

The Poly ADP‐ribose polymerase family is a series of enzymes, where the best characterised members are nuclear proteins which are thought to function by binding to single strand breaks in DNA, allowing the recruitment of repair enzymes by the synthesis of NAD‐derived ADP‐ribose polymers, which are subsequently degraded by a glycohydrolase (PARG, Q86W56).

**Table:** 

Nomenclature	poly(ADP‐ribose) polymerase 1	poly(ADP‐ribose) polymerase 2	poly (ADP‐ribose) polymerase 3
HGNC, UniProt	PARP1, P09874	PARP2, Q9UGN5	PARP3, Q9Y6F1
EC number	2.4.2.30	2.4.2.30	–
Common abreviation	PARP1	PARP2	PARP3
Selective inhibitors	AG14361 (p*K*_i_ 8.2) [483]	–	–

### Further reading on Poly(ADP‐ribose)polymerases

Bai P. (2015) Biology of Poly(ADP‐Ribose) Polymerases: The Factotums of Cell Maintenance. Mol Cell 58: 947‐58 [PMID:26091343]

Bai P et al. (2015) Poly(ADP‐ribose) polymerases as modulators of mitochondrial activity. Trends Endocrinol Metab 26: 75‐83 [PMID:25497347]

Bock FJ et al. (2016) New directions in poly(ADP‐ribose) polymerase biology. FEBS J 283: 4017‐4031 [PMID:27087568]

Bock FJ et al. (2015) RNA Regulation by Poly(ADP‐Ribose) Polymerases. Mol Cell 58: 959‐69 [PMID:26091344]

Ryu KW et al. (2015) New facets in the regulation of gene expression by ADP‐ribosylation and poly(ADP‐ribose) polymerases. Chem Rev 115: 2453‐81 [PMID:25575290]

## 2.5.1.58 Protein farnesyltransferase

### Overview

Farnesyltransferase is a member of the prenyltransferases family which also includes geranylgeranyltransferase types I (EC 2.5.1.59) and II (EC 2.5.1.60) [72]. Protein farnesyltransferase catalyses the post‐translational formation of a thioether linkage between the C‐1 of an isoprenyl group and a cysteine residue fourth from the C‐terminus of a protein (ie to the CaaX motif, where 'a' is an aliphatic amino acid and 'X' is usually serine, methionine, alanine or glutamine; leucine for EC 2.5.1.59) [165]. Farnesyltransferase is a dimer, composed of an alpha and beta subunit and requires Mg^2+^ and Zn^2+^ ions as cofactors. The active site is located between the subunits. Prenylation creates a hydrophobic domain on protein tails which acts as a membrane anchor.

Substrates of the prenyltransferases include Ras, Rho, Rab, other Ras‐related small GTP‐binding proteins, G‐protein *γ*‐subunits, nuclear lamins, centromeric proteins and many proteins involved in visual signal transduction.

In relation to the causative association between oncogenic Ras proteins and cancer, farnesyltransferase has become an important mechanistic drug discovery target.

Information on members of this family may be found in the online database.

### Further reading on Protein farnesyltransferase

Gao S et al. (2016) The Role of Geranylgeranyltransferase I‐Mediated Protein Prenylation in the Brain. Mol Neurobiol 53: 6925‐6937 [PMID:26666664]

Shen M et al. (2015) Farnesyltransferase and geranylgeranyltransferase I: structures, mechanism, inhibitors and molecular modeling. Drug Discov Today 20: 267‐76 https://www.ncbi.nlm.nih.gov/pubmed/25450772[PMID:25450772]

Shen Y et al. (2015) The Recent Development of Farnesyltransferase Inhibitors as Anticancer and Antimalarial Agents. Mini Rev Med Chem 15: 837‐57 [PMID:25963569]

Wang M et al. (2016) Protein prenylation: unique fats make their mark on biology. Nat Rev Mol Cell Biol 17: 110‐22 [PMID:26790532]

## 3.5.1.‐ Histone deacetylases (HDACs)

### Overview

Histone deacetylases act as erasers of epigenetic acetylation marks on lysine residues in histones. Removal of the acetyl groups facilitates tighter packing of chromatin (heterochromatin formation) leading to transcriptional repression.

The histone deacetylase family has been classified in to five subfamilies based on phylogenetic comparison with yeast homologues:

Class I contains HDACs 1, 2, 3 and 8

Class IIa contains HDACs 4, 5, 7 and 9

Class IIb contains HDACs 6 and 10

Class III contains the sirtuins (SIRT1‐7)

Class IV contains only HDAC11.

Classes I, II and IV use Zn^+^ as a co‐factor, whereas catalysis by Class III enzymes requires NAD^+^ as a co‐factor, and members of this subfamily have ADP‐ribosylase activity in addition to protein deacetylase function [456].

HDACs have more general protein deacetylase activity, being able to deacetylate lysine residues in non‐histone proteins [91] such as microtubules [233], the hsp90 chaperone [281] and the tumour suppressor p53 [322].

Dysregulated HDAC activity has been identified in cancer cells and tumour tissues [305, 444], making HDACs attractive molecular targets in the search for novel mechanisms to treat cancer [567]. Several small molecule HDAC inhibitors are already approved for clinical use: romidepsin, belinostat, vorinostat, panobinostat, belinostat, valproic acid and tucidinostat. HDACs and HDAC inhibitors currently in development as potential anti‐cancer therapeutics are reviewed by Simó‐Riudalbas and Esteller (2015) [478].

Information on members of this family may be found in the online database.

### Further reading on Histone deacetylases

Maolanon AR et al. (2017) Natural and Synthetic Macrocyclic Inhibitors of the Histone Deacetylase Enzymes. Chembiochem 18: 5‐49 [PMID:27748555]

Micelli C et al. (2015) Histone deacetylases: structural determinants of inhibitor selectivity. Drug Discov Today 20: 718‐35 [PMID:25687212]

Millard CJ et al. (2017) Targeting Class I Histone Deacetylases in a “Complex” Environment. Trends Pharmacol Sci 38: 363‐377 [PMID:28139258]

Roche J et al. (2016) Inside HDACs with more selective HDAC inhibitors. Eur J Med Chem 121: 451‐83 [PMID:27318122]

Zagni C et al. (2017) The Search for Potent, Small‐Molecule HDACIs in Cancer Treatment: A Decade After Vorinostat. Med Res Rev [PMID:28181261]

## 3.5.3.15 Peptidyl arginine deiminases (PADI)

### Overview

In humans, the peptidyl arginine deiminases (PADIs; HGNC family link) are a family of five enzymes, PADI1‐4 and PADI6. PADIs catalyze the deimination of protein L‐arginine residues to L‐citrulline and ammonia, generating peptidyl‐citrulline on histones, fibrinogen, and other biologically relevant proteins. The human isozymes exhibit tissue‐specific expression patterns [256]. Overexpression and/or increased PADI activity is observed in several diseases, including rheumatoid arthritis, Alzheimer's disease, multiple sclerosis, lupus, Parkinson's disease, and cancer [37]. Pharmacological PADI inhibition reverses protein‐hypercitrullination and disease in mouse models of multiple sclerosis [366].

Information on members of this family may be found in the online database.

### Further reading on Peptidyl arginine deiminases

Koushik S et al. (2017) PAD4: pathophysiology, current therapeutics and future perspective in rheumatoid arthritis. Expert Opin Ther Targets 21: 433‐447 [PMID:28281906]

Tu R et al. (2016) Peptidyl Arginine Deiminases and Neurodegenerative Diseases. Curr Med Chem 23: 104‐14 [PMID:26577926]

Whiteley CG. (2014) Arginine metabolising enzymes as targets against Alzheimers' disease. Neurochem Int 67: 23‐31 [PMID:24508404]

## RAS subfamily

### Overview

The RAS proteins (HRAS, NRAS and KRAS) are small membrane‐localised G protein‐like molecules of 21 kd. They act as an on/off switch linking receptor and non‐receptor tyrosine kinase activation to downstream cytoplasmic or nuclear events. Binding of GTP activates the switch, and hydrolysis of the GTP to GDP inactivates the switch.

The RAS proto‐oncogenes are the most frequently mutated class of proteins in human cancers. Common mutations compromise the GTP‐hydrolysing ability of the proteins causing constitutive activation [495], which leads to increased cell proliferation and decreased apoptosis [598]. Because of their importance in oncogenic transformation these proteins have become the targets of intense drug discovery effort [25].

Information on members of this family may be found in the online database.

### Further reading on RAS subfamily

Dorard C et al. (2017) Deciphering the RAS/ERK pathway in vivo Biochem Soc Trans 45: 27‐36 [PMID:28202657]

Keeton AB et al. (2017) The RAS‐Effector Interaction as a Drug Target. Cancer Res 77: 221‐226 [PMID:28062402]

Lu S et al. (2016) Ras Conformational Ensembles, Allostery, and Signaling. Chem Rev 116: 6607‐65 [PMID:26815308]

Ostrem JM et al. (2016) Direct small‐molecule inhibitors of KRAS: from structural insights to mechanism‐based design. Nat Rev Drug Discov 15: 771‐785 [PMID:27469033]

Papke B et al. (2017) Drugging RAS: Know the enemy. Science 355: 1158‐1163 [PMID:28302824]

Quah SY et al. (2016) Pharmacological modulation of oncogenic Ras by natural products and their derivatives: Renewed hope in the discovery of novel anti‐Ras drugs. Pharmacol Ther 162: 35‐57 [PMID:27016467]

Simanshu DK et al. (2017) RAS Proteins and Their Regulators in Human Disease. Cell 170: 17‐33 [PMID:28666118]

## 4.2.1.1 Carbonate dehydratases

### Overview

Carbonic anhydrases facilitate the interconversion of water and carbon dioxide with bicarbonate ions and protons (EC 4.2.1.1), with over a dozen gene products identified in man. The enzymes function in acid‐base balance and the movement of carbon dioxide and water. They are targetted for therapeutic gain by particular antiglaucoma agents and diuretics.

**Table:** 

Nomenclature	carbonic anhydrase 1	carbonic anhydrase 7	carbonic anhydrase 12
HGNC, UniProt	CA1, P00915	CA7, P43166	CA12, O43570
EC number	4.2.1.1	4.2.1.1	4.2.1.1
Inhibitors	chlorthalidone (p*K*_i_ 6.5)	methazolamide (p*K*_i_ 8.7) [467], acetazolamide (p*K*_i_ 8.6) [19], brinzolamide (p*K*_i_ 8.6) [467], chlorthalidone (p*K*_i_ 8.6) [524]	chlorthalidone (p*K*_i_ 8.4) [524], diclofenamide (p*K*_i_ 7.3) [547]

### Further reading on 4.2.1.1 Carbonic anhydrases

Frost SC. (2014) Physiological functions of the alpha class of carbonic anhydrases. Subcell Biochem 75: 9‐30 [PMID:24146372]

Supuran CT. (2017) Advances in structure‐based drug discovery of carbonic anhydrase inhibitors. Expert Opin Drug Discov 12: 61‐88 [PMID:27783541]

Supuran CT. (2016) Structure and function of carbonic anhydrases. Biochem J 473: 2023‐32 [PMID:27407171]

## 5.99.1.2 DNA Topoisomerases

### Overview

DNA topoisomerases regulate the supercoiling of nuclear DNA to influence the capacity for replication or transcription. The enzymatic function of this series of enzymes involves cutting the DNA to allow unwinding, followed by re‐attachment to reseal the backbone. Members of the family are targetted in anti‐cancer chemotherapy.

**Table:** 

Nomenclature	topoisomerase (DNA) I	topoisomerase (DNA) II alpha
HGNC, UniProt	TOP1, P11387	TOP2A, P11388
EC number	5.99.1.2	5.99.1.2
Inhibitors	irinotecan [125, 518] – Bovine	etoposide (pIC_50_ 7.3), teniposide [127] – Mouse

### Further reading on DNA topoisomerases

Bansal S et al. (2017) Topoisomerases: Resistance versus Sensitivity, How Far We Can Go? Med Res Rev 37: 404‐438 [PMID:27687257]

Capranico G et al. (2017) Type I DNA Topoisomerases. J Med Chem 60: 2169‐2192 [PMID:28072526]

Nagaraja V et al. (2017) DNA topoisomerase I and DNA gyrase as targets for TB therapy. Drug Discov Today 22: 510‐518 [PMID:27856347]

Pommier Y et al. (2016) Roles of eukaryotic topoisomerases in transcription, replication and genomic stability. Nat Rev Mol Cell Biol 17: 703‐721 [PMID:27649880]

Seol Y et al. (2016) The dynamic interplay between DNA topoisomerases and DNA topology. Biophys Rev 8: 101‐111 [PMID:28510219]
